# T Cells of Infants Are Mature, but Hyporeactive Due to Limited Ca^2+^ Influx

**DOI:** 10.1371/journal.pone.0166633

**Published:** 2016-11-28

**Authors:** Kristin Schmiedeberg, Hardy Krause, Friedrich-Wilhelm Röhl, Roland Hartig, Gerhard Jorch, Monika C. Brunner-Weinzierl

**Affiliations:** 1 Department of Experimental Pediatrics, University Hospital, Otto-von-Guericke University, Magdeburg, Germany; 2 Clinic of Pediatric Surgery University Hospital, Otto-von-Guericke University, Magdeburg, Germany; 3 Institute of Biometry and Medical Informatics University Hospital, Otto-von-Guericke University, Magdeburg, Germany; 4 Institute for Molecular and Clinical Immunology, University Hospital, Otto-von-Guericke University, Magdeburg, Germany; Monash University, AUSTRALIA

## Abstract

CD4 T cells in human infants and adults differ in the initiation and strength of their responses. The molecular basis for these differences is not yet understood. To address this the principle key molecular events of TCR- and CD28-induced signaling in naive CD4 T cells, such as Ca^2+^ influx, NFAT expression, phosphorylation and translocation into the nucleus, ERK activation and IL-2 response, were analyzed over at least the first 3 years of life. We report dramatically reduced IL-2 and TNFα responses in naive CD31^+^ T cells during infancy. Looking at the obligatory Ca^2+^ influx required to induce T cell activation and proliferation, we demonstrate characteristic patterns of impairment for each stage of infancy that are partly due to the differential usage of Ca^2+^ stores. Consistent with those findings, translocation of NFATc2 is limited, but still dependent on Ca^2+^ influx as demonstrated by sensitivity to cyclosporin A (CsA) treatment. Thus weak Ca^2+^ influx functions as a catalyst for the implementation of restricted IL-2 response in T cells during infancy. Our studies also define limited mobilization of Ca^2+^ ions as a characteristic property of T cells during infancy. This work adds to our understanding of infants’ poor T cell responsiveness against pathogens.

## Introduction

The mammalian adaptive immune system provides specific and long-lasting protection against pathogens. Beginning with the first day of life, this system must effectively battle the acute threat of microbial invasion without harming endogenous systems. At the same time, the adaptive immune system must learn to tolerate innocuous antigens from the environment. In this challenging time period for the immune system, epidemiological studies have shown that neonates and infants are especially susceptible to infections; this period of life is also decisive for directing immune responses and pathologies later in life [1]. T cell functions, such as cytokine production, are downregulated in response to antigens of acute infections during the neonatal and early infancy periods [2,3]. This has been attributed to reduced numbers of neonatal lymphocytes [4,5] and to those cells’ attenuated ability to be fully activated because of limited expression of activation-associated molecules such as CD40L and NFAT [6–9].

Naive T cells can be divided into two subpopulations: recent thymic emigrants (RTE) and T cells that have already proliferated homeostatically in the periphery. RTEs are likely the predominant population of T cells in infants. They can be identified among CD45RA^+^CD4^+^ T cells by T cell receptor (TCR) excision circles, and the surrogate markers CD31 for RTEs or CCR7 and CD62L for naive T-cells [10,11]. T cell activation has been extensively analyzed in adults where optimal activation requires two signals: one is transmitted through the TCR and the second through costimulatory molecules [12], whereby the primary costimulatory molecule is CD28 [13–15].

For T cell activation, three main signal transduction pathways are initiated by TCR/CD28 signaling which leads to expression and translocation of the transcription factors NF-κB (nuclear factor kappa-light-chain enhancer of activated B cells), AP-1 (activator protein 1), and NFATc (nuclear factor of activated T cells cytoplasmic) into the nucleus (see below) [16]. Their binding to the IL-2 promoter is an obligatory prerequisite for IL-2 transcription. Although the pathways for their activation/translocation interconnect, it is clear that distinct signaling events are essential for full T cell activation such as Ca^2+^-dependent dephosphorylation of NFAT and ERK1/2 activation for AP-1 translocation.

T cell activation requires the coupling of TCR to several signal transduction cascades, via kinases and adaptor proteins such as Fyn, Lck, ZAP-70 to LAT phosphorylation. One of these cascades is triggered when TCR ligation results in Vav and Sos activation running into MAP-Kinase Raf-MEK-ERK axis leading to formation and translocation of AP-1 transcription factors. Two further cascades are initiated when TCR ligation recruits LAT, which then interacts with PLCγ (phospholipase C gamma) generating IP_3_ (inositol-1,4,5-trisphosphate) and DAG (diacylglycerol). This splits into two different signaling pathways. First, DAG involves the protein kinase C theta (PKCθ) and this leads to activation of NF-κB, which can be regulated by PI3K activation via CD28 stimulation. In addition, CD28 engagement can also influence the Raf-MEK-ERK module via Grb2 and Vav interaction. Of special interest in early T-cell activation is the NFATc pathway where IP_3_ generated by PLCγ binds to the IP_3_ receptor and causes the release of calcium (Ca^2+^) from the endoplasmatic reticulum (ER). This Ca^2+^ depletion is sensed by Stromal Interacting Molecule1 (STIM1), which relocates by forming “puncta” and couples directly to the ORAI CRAC channels in the plasma membrane. This results in an influx of extracellular Ca^2+^ into the cytosol and activates calcineurin (CaN), a Ca^2+^ and calmodulin dependent phosphatase, leading to the dephosphorylation and nuclear translocation of NFATc [7,17]. To date, only Ca^2+^ dependent CaN is known to dephosphorylate NFATc. All 3 pathways are important since all transcription factors cooperate and interact with each other and leads to an increase or inhibition of transcription of the gene coding for IL-2 [18].

Ca^2+^ influx acts early in the activation cascade, before the subsequent formation of a membrane complex composed of the actin cytoskeleton, membrane rafts, TCR, and TCR proximal proteins is needed. This complex formation is obligatory for full T cell activation [19]. Ca^2+^ influx is a check point of T cell activation since the increase of the intracellular Ca^2+^ ion concentration plays an essential role in lymphocyte activation and maturation by control of a diverse range of cell functions, including adhesion, motility, gene expression and proliferation [7]. Ca^2+^ signaling patterns can occur as single transients, as repetitive oscillations or as a sustained plateau. The amplitude and duration of Ca^2+^ signals matters, as it has been shown to differentially control the pro-inflammatory transcriptional regulators NF-κB, c-Jun and NFAT [20] making its tight control obligatory to avoid misdirected responses. How the TCR signaling and CD28 costimulation, which have been studied extensively in adults, contribute to the molecular processes of T cell activation in neonates and infants is not yet understood. As Ca^2+^ influx is one of the earliest key events during T cell activation, we hypothesize that its altered usage in human neonates and infant CD4 T cells could be a reason for their signatory cytokine production.

Here, we investigated key molecular events in the main signaling pathways of CD4^+^CD45RA^+^CD31^+^ naive T cells during infancy to evaluate their immune competence and ability to fight infections. We used the available Ca^2+^ as a measure of a central regulatory event, and show that TCR cross-linking with a soluble anti-CD3 antibody (Ab) in naive T cells of infants 3–5 months (mo) resulted in a dramatically low Ca^2+^ influx. Furthermore, we found little or no CD28 costimulatory dependence for the Ca^2+^ influx in the infants’ CD4^+^CD45RA^+^CD31^+^ T cells. Indeed, this remarkably ineffective costimulation extended to the degree of NFAT expression and, ultimately, to the expression of “early” cytokines such as TNFα and IL-2. This insight into the special characteristics of T cell activation during the first year of life could explain the poor responsiveness of these cells to acute provocation by pathogens and help explain infants’ susceptibility to infections.

## Materials and Methods

### Ethics statement

The study was approved by the Clinical Research Ethics Board of the Otto-von-Guericke University Magdeburg (certificate 79/07), and all of the donors or their parents provided written informed consent in accordance with the declaration of Helsinki.

### Samples

A total of 114 peripheral blood samples were collected from healthy white adults and from infants/children between 0.1 and 66 months. Adult blood donors, mostly white (81%) males, ranged from 18 to 50 years in age. Peripheral blood mononuclear cells (PBMCs) were obtained from leukocyte reduction filters (Sepacell RZ-2000, Asahi Kasei Medical Co., Chiyoda-ku, Tokyo, Japan) at the Institute of Transfusion Medicine and Immunohematology and at the blood bank at the University Clinic of Magdeburg. Human cord blood (CB) samples were obtained from umbilical cord veins immediately after placental delivery at the Women’s Clinic of the University Hospital of Magdeburg.

### T cell preparation and culture

PBMCs from adult leukocyte reduction filters [21,22], CB samples, and peripheral blood were isolated by Ficoll^™^ gradient centrifugation (Biochrom, Berlin, Germany). CD4^+^CD45RA^+^CD31^+^ naive T cells were enriched, using a CD4^+^ T cell Isolation Kit II and AutoMACS (magnetic-assisted cell sorting) (Miltenyi Biotec, Bergisch-Gladbach, Germany) followed by negative selection by CD45RO Micro Beads and positive selection by anti-CD31 Micro Beads (Miltenyi Biotec) The purified CD45RA^+^ fractions contained 95–97% CD45RA^+^ cells, and the purified CD31^+^ fractions contained 87–99% CD31^+^ T cells. The cells were cultivated at 37°C in RPMI 1640 medium (Biochrom, Berlin, Germany), supplemented with penicillin-streptomycin and with 10% human AB-plasma. A total of 2×10^6^ T cells/ml were loaded with anti-CD3 Ab (0.5 μg/ml) plus anti-CD28 Ab (0.5 μg/ml) for two minutes, then stimulation was performed by cross-linking the Abs with 10 μg/ml goat anti-mouse IgG (GAMIg) (Invitrogen, Darmstadt, Germany).

### Cytokine quantification

Cytokines in culture supernatants were quantified using multiplex immunoassays like the Bio-Plex cytokine assay (IFNγ, IL-2, TNFα; Bio-Rad, München, Germany) after 24 h stimulation for IL-2 and TNFα [23,24] or, as a control for T cell functionality, IFNγ after 48 h stimulation [25]. Briefly, cytokine standards or samples were incubated with anti-cytokine conjugated beads, followed by detection Ab and streptavidin-phycoerythrin. Samples were analyzed on a Bio-Rad 96-well plate reader using the Bio-Plex Suspension Array System and Bio-Plex Manager Software (Bio-Rad, München, Germany).

### Flow cytometric analysis and Ca^2+^ influx measurements

Freshly isolated PBMCs (1–2 ×10^6^ cells/ml) were loaded with 3 μM Indo-1-AM (4-(6-carboxy-2-indolyl)-4′-methyl-2,2′-(ethylenedioxy)dianiline-N,N,N′,N′-tetraacetic acid tetrakis (acetoxymethyl)ester) in RPMI1640 medium (phenol-red free) containing 10% fetal calf serum at 37°C for 45 min (Invitrogen, Darmstadt, Germany). Samples were washed, incubated for another 45 min at 37°C in RPMI 1640 containing 10% FCS and labeled with anti-CD4-allophycocyanin (-APC), anti-CD31-PE, and anti-CD45RA-FITC (all from BD, Bioscience, Heidelberg, Germany). After washing the samples were analyzed using a flow cytometer (LSR I BD Bioscience, Heidelberg, Germany). Kinetics of the Ca^2+^ influx were analyzed using FlowJo software (Treestar, Palo Alto, CA, US). Ca^2+^ influx in CD4^+^ T cells were performed in response with varying soluble anti-CD3 Ab concentrations plus 0.5 μg/ml soluble anti-CD28 Ab or anti-CD3 Ab in combination with GAMIg. GAMIg was added to cross-link the antibodies. Under these conditions, no signals were obtained from the T cells treated only with GAMIg or anti-CD28 Ab plus GAMIg. At the end of the measurement, ionomycin was added to guarantee the vitality of the cells, to determine the maximal Ca^2+^ release response and to normalize the responsiveness of the cells of individual samples. The equation was for calculation the max. Ca^2+^ response (normalized to ionomycin): normalization to max. Ca^2+^ influx signal of ionomycin = (max. GAMIg Ca^2+^ influx signal peak—baseline) / (max. ionomycin Ca^2+^ influx signal peak—baseline).

### Immunoblotting and protein quantification

**Fig 1:**
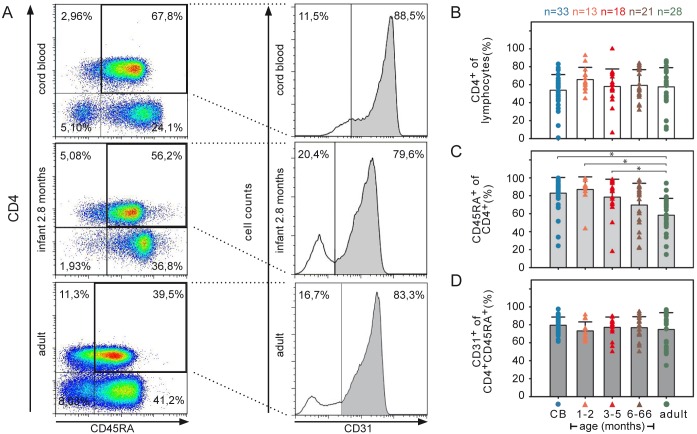
Frequencies of naive CD31^+^ T cells within the CD4^+^CD45RA^+^ compartment remain constant over ages. (A) T cell subsets of CB or PBMCs analyzed by flow cytometry. Representative flow cytometry dot plots and histograms are shown with the percentage of CD4^+^CD45RA^+^ T cell subsets for CB (top), infant (middle; 2.8 months), and adult (bottom) samples. (B) Frequencies of CD4^+^ among lymphocytes, (C) of CD45RA^+^ among CD4^+^ T cells, and (D) CD31^+^ among peripheral CD4^+^CD45^+^ cells are shown (* *P<*0.05; two-sided ANOVA Tukey-Kramer post-hoc test). The mean value and standard deviations (SD) are indicated. Age of infants and children is indicated in months. n = number of individuals.

Naive T cells were immediately enriched age-dependently to achieve a similar high degree of enrichment of naive T cells with the lowest cell loss (see also Fig 1). From adults, proteins were extracted from CD4^+^CD45RA^+^CD31^+^ adult T cells and from CB and infants from CD4^+^ sorted PBMCs. A total volume of 200–500 μl blood from infants was obtained and at least 0.1–1.0x10^6^ CD4^+^ T cells were acquired. At the end of each stimulation, for SDS-PAGE and Western blot analysis, individual samples (of 3–5 donors) were pooled after snap freezing to provide comparable cell numbers. Whole cell lysates were prepared in hypotonic buffer (pH 7.5, 20 mM HEPES, 20 mM NaF, 5 mM EDTA, 1% NP-40, 0.1 mM PMSF, 40 mM β-glycerophosphate, 2 mM Na_3_VO_4_, complete protease inhibitors cocktail tablets (Roche, Mannheim, Germany)).

Nuclear extracts were prepared from PBMC according to commonly used protocols. Briefly, 1–2 x 10^7^ cells were washed with PBS/4°C and resuspended in cell lysis buffer (10 mM HEPES (pH 7.9), 10 mM KCl, 0.1 mM EDTA, 0.1 mM EGTA, 1 mM PMSF, 1 mM DTT, complete protease inhibitors cocktail tablets (Roche, Mannheim, Germany)), swelled on ice for 10 min, followed by vortex mixing for 30 s. The homogenate was aspirated with an 18 guage syringe needle and centrifuged at 10.000 x g (15 min, 4°C). The supernatant (cytoplasmic protein) was snap frozen at -196°C. The nuclear pellet was resuspended in nuclear extraction buffer (20 mM HEPES (pH 7.9), 0.4 M NaCl, 1 mM EDTA, 1 mM EGTA, 1 mM PMSF, 1 mM DTT, protease inhibitors) and mixed for 1 h. The nuclear extract was obtained by centrifuging at 10.000 x g for 15 min at 4°C.

Extracted proteins were separated on 10% SDS-PAGE and transferred to a nitrocellulose membrane (Bio-Rad, München, Germany). For Western blot analysis the membrane was probed with Ab against anti-NFATc2, anti-ERK1 (all from Santa Cruz, Biotechnology Inc., Heidelberg, Germany) or anti-STIM1, anti-pERK1/2 (Thr202/Tyr204) and anti-αTubulin (DM1A) (all from Cell Signaling, NEB, Frankfurt, Germany). Quantitation was performed using TINA-software 2.09f (Raytest, Straubenhardt, Germany).

### Statistical analysis

With ANOVA assessment of relative frequencies of naive CD4^+^ T cells, cytokine concentrations in supernatants and Ca^2+^ influx, the main effect was part of the model. To test whether T cell activation was dependent on CD28 costimulation, age, stimulation, and cross effects between age and stimulation were assessed. A mixed-model ANOVA was performed with individual as random effect followed by Tukey-Kramer post-hoc test for pairwise group comparisons. All statistical tests were two-sided and p values of 0.05 or less were considered significant. With the exception of a post-hoc test to verify the age effect, all tests were performed without α adjustment. For that reason the results should be interpreted as being exploratory in nature. Statistics were performed using SAS^®^ software (V.9.2, SAS, Institute Inc., Cary, NC, USA).

## Results

### Cord blood (CB) and infants have high relative frequencies of naive CD4^+^ T cell subpopulations

The frequency of naive lymphocyte subsets changes over the course of life [26–28], but whether the functionality of these naive T cells remains constant throughout life has not been investigated. To define the composition of the CD4 T cell population in 33 CB, 52 pediatric (infant/child), and 28 adult blood samples were analyzed using flow cytometry (Fig 1A).

Whereas all samples showed similar frequencies of CD4^+^ T cells (Fig 1B), flow cytometry analysis of CD45RA expression showed decreasing frequencies of CD4^+^CD45RA^+^ lymphocytes with increasing age (ANOVA *P<*0.0001, S1 and S2 Tables). The frequency of CD4^+^CD45RA^+^ T cells in the CD4^+^ T cell pool was the highest for infants 1–2 months and CB (Fig 1C) and decreased by 30% by adulthood (ANOVA *P<*0.05, S3 Table). The frequency of CD4^+^CD45RA^+^CD31^+^ thymic naive T cells (RTEs) [10] among the CD4^+^CD45RA^+^ T cells (Fig 1D) remained similar over the analyzed subgroups. However, the individual variation was greater among adult donors. The naive T cell subpopulations (CD3^+^CD4^+^CD45RA^+^) of all donors analyzed expressed CD28 at similar frequencies of >96% (data not shown). Thus, only the CD4^+^CD45RA^+^ T cells decreased by adulthood but T cells of the CD31^+^ naive subtype (RTE) are—relative to CD4^+^CD45RA^+^ T cells—abundantly present throughout life.

These results showed us also, how to get the most naive T cells efficiently enriched from the small samples available from CB and infants. In CB, since almost all T cells are naive, we were able to enrich naive T cells of CB by CD4^+^ selection only. In infants, as the CD45RA^+^ compartments looks the same as from CB, RA^+^ T cell enrichment accounted for naive T cells up to 5 month of age. In adults, we routinely isolated CD4^+^CD45RA^+^CD31^+^ T cells to unambiguously work with naive T cells only. Thus, enrichment of CD4^+^ cells from CB, CD4^+^CD45RA^+^ cells from infants, and CD4^+^RA^+^CD31^+^ T cells all show high accumulation of CD4^+^CD45RA^+^CD31^+^ T cells and, thus, are considered to be naive T cells.

### Recent thymic emigrants (RTE) are efficient naive T cells

**Fig 2:**
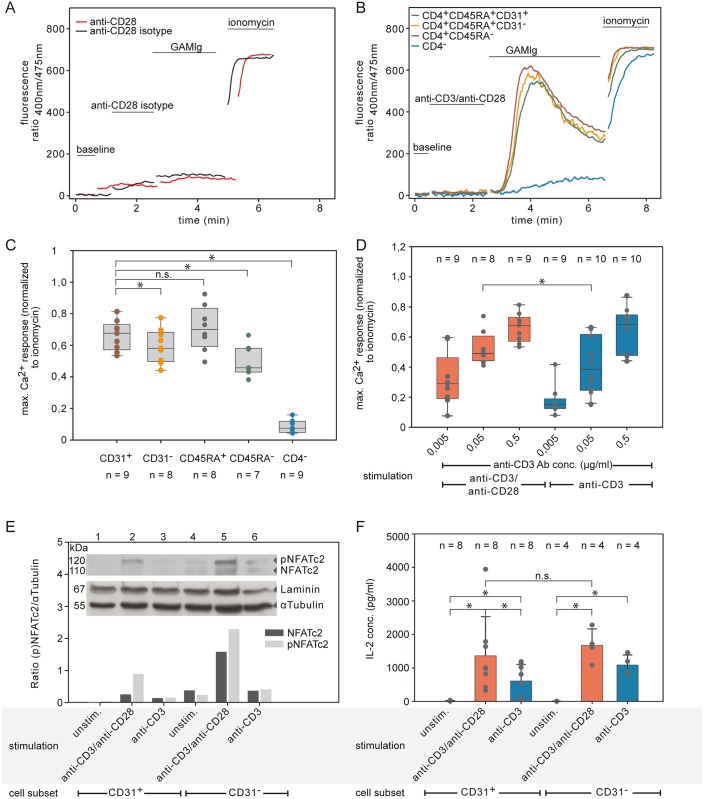
Ca^2+^ influx is a key event for IL-2 in adult CD31^+^ naive T cells. (A) No Ca^2+^ influx signal with GAMIg alone (black curve; anti-CD28 isotype Ab were substituted for anti-CD28 Ab) or anti-CD28 Ab plus GAMIg alone (red curve) in T cells stained for anti-CD4, anti-CD45, and anti-CD31. Results representative of at least 3 experiments are shown. (B-C) Ca^2+^ influx into adult CD31^+^ naive T cells stimulated using anti CD3 Ab and anti CD28 Ab. (B) A representative Ca^2+^ influx experiment and (C) box plots for the different T cell subsets showing scatter plots of mean value and SD of Ca^2+^ influx experiments by normalizing maximal Ca^2+^ signals to the maximal Ca^2+^ influx ionomycin are shown (* *P<*0.05; two-tailed ANOVA of differences). (D) Increased Ca^2+^ influx in adult CD31^+^ naive T cells compared with anti-CD3/anti-CD28 Ab (red) in response to anti-CD3 Ab alone (blue, with anti-CD28 isotype Ab) (* *P<*0.05; two-tailed ANOVA of differences) (E) Increased levels of NFATc2 protein expression in response to anti-CD3/anti-CD28 Ab stimulation in naive T cells. The immunoblot detection of the relative protein expression level by ratio of NFATc2 or pNFATc2 to αTubulin is shown for naive CD31^+^ or CD31^-^ T cells of adults stimulated with anti-CD3 Ab in combination with soluble anti-CD28 Ab (dark gray) or anti-CD28 Ab isotype (light gray). Laminin and αTubulin were used as loading controls. Results are representative of at least two experiments. unstim. = unstimulated. (F) IL-2 cytokine in supernatants does not differ in cultures under TCR/CD3 stimulation between subtypes of naive CD4 T cells (* *P<*0.05; two-tailed ANOVA of differences). ns = not significant.

The rise of the basal Ca^2+^ level is a key signaling event in T cells that leads to NFAT translocation into the nucleus and, ultimately, to increased cytokine production [29]. To analyze the efficiency of the Ca^2+^ influx during T cell stimulation, the cells were treated with varying concentrations of anti-CD3 Ab with anti-CD28 Ab or with anti-CD28 Ab isotype. The antibodies used for characterizing T cells (anti-CD4, anti-CD45, and anti-CD31) show no interference with the anti-CD3 Ab triggered Ca^2+^ influx, because no signals where obtained by treating T cells only with the goat anti-mouse IgG (GAMIg) used to cross-link the antibodies or with the anti-CD28 Ab plus GAMIg alone (Fig 2A).

The magnitude of the Ca^2+^ response has been shown to correlate with reactivity in a number of lymphocyte subpopulations from adults [30–32]. Here, the subset of CD31^+^ naive T cells (RTE) and the CD4^+^CD45RA^+^ T cells were the most responsive populations in adults and both showed similar large Ca^2+^ influx responses (Fig 2B and 2C, two-tailed ANOVA of differences *P<*0.05, S4 and S5 Tables). Furthermore, as has been described in studies before [13] the addition of costimulation in the form of anti-CD28 Ab significantly increased Ca^2+^ influx in adult CD31^+^ naive T cells compared with anti-CD3 Ab alone (Fig 2D, ANOVA test of difference *P<*0.05, S6 Table).

To investigate the NFAT-axis of signal transduction, we measured expression and phosphorylation status of the main available member NFATc2, (NFAT1) [33] in response to stimulation of naive T cells. NFATc2 protein expression was clearly observed in adult naive T cells (CD4^+^CD45RA^+^CD31^+^ and CD4^+^CD45RA^+^CD31^-^) (Fig 2E, lanes 2 and 5) after anti-CD3 Ab plus anti-CD28 Ab engagement, and was higher than in cells treated only with anti-CD3 Ab (Fig 2E, lanes 3 and 6). Upon activation, there was an increase in phosphorylated NFATc2 mainly in CD31^-^ naive T cells. However, for the early cytokine IL-2, no significant differences between subtypes of naive T cells were detected (Fig 2F, two-tailed ANOVA of differences *P* = 0,629, S17 Table) under the same stimulation. Thus, even though differences at the molecular level exist between subtypes of naive T cells, the outcome—namely IL-2 concentrations in the supernatants—is similar.

### Signaling pathways obligatory for IL-2 transcription are already available in CB T cells

**Fig 3:**
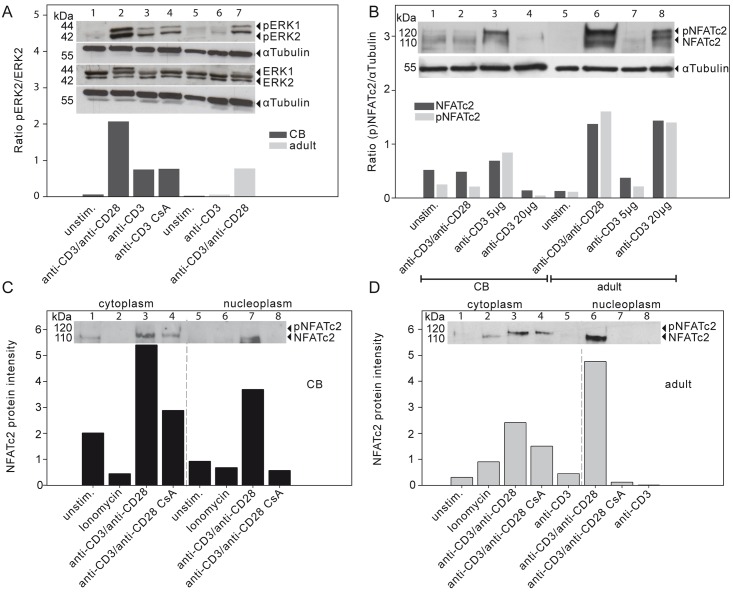
Key signaling pathways for IL-2 transcription are activated in TCR/CD3 stimulated naive CD4^+^ T cells of CB. (A) Protein expression by Western blot of ERK1/2 and phosphorylated ERK1/2Tyr202/ Tyr204 (pERK1/2) in naive CD4^+^ T cells of CB as well as for naive CD31^+^ adult T cells. The ratios of relative protein expression levels are indicated below the respective bands. Data are representative of two independent experiments. (B) Whole cell protein extract of NFATc2 and phosphorylated NFATc2 (pNFATc2) in CB naive CD4^+^ T cells and adult naive CD31^+^ T cells under different stimulation conditions. The densitometric analyses of the immunoblot detection for the relative protein expression level are shown as ratio of NFATc2 or pNFATc2 to αTubulin. Lysates from three different donors were pooled. Data are representative of at least three independent experiments. αTubulin was used as a loading control. (C-D). The NFATc2 protein expression in cytoplasm or nucleoplasm (separated through a dashed line) in (C) naive CD4^+^ T cells of CB or (D) CD31^+^ naive T cells of adult were detected and the phosphorylated (pNFATc2) and dephosphorylated (NFATc2) forms quantified. Cells were stimulated as indicated in the presence or absence of cyclosporin A (CsA). One representative experiment out of two comparable experiments is shown. unstim. = unstimulated.

To analyze the activation of the MAPK-ERK pathway, which leads to AP-1 translocation into the nucleus, we used CB as an example for the “youngest” T cells, which produced only low amounts of IL-2 [2,3], in comparison to adult CD31^+^ naive T cells. Strikingly, the MAPK-ERK axis of naive CD4^+^ T cells responded robustly by phosphorylation of ERK. The strongest response was observed using anti-CD3 Ab TCR engagement plus anti-CD28 Ab costimulation in CB (Fig 3A, lanes 2).

The ratio of pERK2/ERK2 was increased by a factor of two to three in naive CB compared to adult CD31^+^ naive T cells under the same condition (Fig 3A, lanes 2 and 7). Interestingly, stimulation of CB only with anti-CD3 Ab TCR engagement without costimulation had the same amount of the pERK2/ERK2 ratio as did adult CD31^+^ naive T cells receiving anti-CD3 Ab plus anti-CD28 Ab (Fig 3A, lanes 3 and 7). When adult CD31^+^ naive T cells were activated by anti-CD3 Ab TCR engagement without costimulation, only low amounts of phosphorylated ERK2 appeared. Thus, already at birth there is a strong expression and activation of ERK upon activation of naive CD4^+^ T cells.

In concordance with the literature, CB CD4^+^ T cells expressed low constitutive levels of NFATc2 [6,8]. However, NFATc2 expression after anti-CD3 Ab and anti-CD28 Ab stimulation was reduced in CB compared to adult CD31^+^ naive T cells (Fig 3B, lane 2 compared to lane 6). Surprisingly low concentrations of anti-CD3 Ab (Fig 3B, lanes 3 and 4) were sufficient to increase the expression of NFATc2 in CB CD4^+^ T cells. This stands in contrast to adult CD31^+^ naive T cells, which required either anti-CD3 Ab plus anti-CD28 Ab engagement or a high concentration of anti-CD3 Ab to result in increased NFATc2 expression (Fig 3B, lanes 6 and 8).

Subcellular fractionation of CB T cells demonstrated that pNFATc2 was already present in the cytoplasm under resting conditions (Fig 3C, CB lanes 1). After anti-CD3/anti-CD28 Ab cross-linking, the dephosphorylation of NFATc2 and its subsequent nuclear localization were lower in CB CD4^+^ T cells than in CD31^+^ naive T cells from adults (Fig 3C, CB lanes 7 compared to Fig 3D, adult lanes 6). As is known for adult T cells, CsA [34–36] prevents the activation of the Ca^2+^ dependent phosphatase calcineurin (CaN), which dephosphorylates NFAT in the cytoplasm. The dephosphorylation of the transcription factor NFAT is obligatory for its nuclear translocation. Binding of Ca^2+^ ions and calmodulin to CaN leads to a change of conformation and a subsequent unmasking of the active center. Thereby, CaN activity is coupled to cytosolic Ca^2+^ levels. CsA binds to the cytosolic protein cyclophilin and this complex inhibits the Ca^2+^ dependent activation of CaN, which unmasks Ca^2+^ independent events of NFAT translocation. The existence of NFATc2 in the nucleoplasm is decreased by CsA treatment in CB as well as in adult naive CD4^+^ T cells (Fig 3C lanes 4 and 8; Fig 3D lanes 4 and 7) and it seems that, NFATc2 expression remains higher after CsA treatment in CB T cells compared to the expression level in adult T cells. However, CsA treatment does abolish most of the NFATc2 expression in the nucleoplasm of CB T cells. Consistent with those findings, the expression and localization of NFATc2 is limited, but still dependent on Ca^2+^ activated CaN as demonstrated by sensitivity to CsA treatment.

### Induced Ca^2+^ influx is higher in CB than in adult CD31^+^ naive T cells

**Fig 4:**
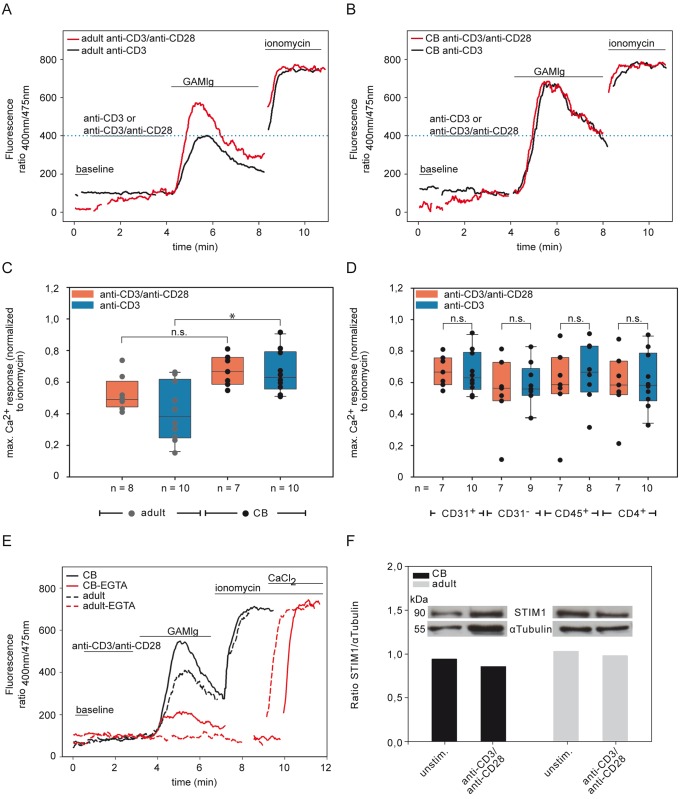
Different Ca^2+^ responses of CB and adult CD31^+^ naive T cells. (A-B) Ca^2+^ mobilization in CD4^+^CD45RA^+^CD31^+^ T cells of one healthy donor (representative of at least eight healthy individuals) of PBMCs (A, adult) or CB (B) were performed in response to 0.05 μg/ml anti-CD3 Ab plus 0.5 μg/ml soluble anti-CD28 Ab (red curve) or only 0.05 μg/ml of anti-CD3 Ab alone (black curve, with anti-CD28 isotype Ab) in combination with GAMIg. The blue dotted line displays the maximum Ca^2+^ response of adult CD31^+^ naive T cells for anti-CD3 Ab stimulation alone. (C) Box plot with scatter plots representing means and SD of Ca^2+^ influx response normalized by maximal Ca^2+^ influx response to ionomycin of adult (gray circle) and CB (black circle) and their dependency on anti-CD28 Ab costimulation (anti-CD3 Ab plus anti-CD28 Ab (red box) or anti-CD3 Ab with anti-CD28 Ab isotype (blue box)). Statistical significance between groups * *P<*0.05 was determined by two-tailed ANOVA with Tukey-Kramer post-hoc test. n = number of individuals. (D) Comparison of different CD4^+^ T cell subset stimulated with anti-CD3 Ab plus anti-CD28 Ab (red box) or with anti-CD3 Ab alone (blue box). Box plot with scatter plots representing means and standard deviations of Ca^2+^ influx response normalized by maximal Ca^2+^ influx response to ionomycin. Statistical significance of differences between anti-CD3/anti-CD28 Ab or anti-CD3 Ab stimulation at concentration 0.05 μg/ml of anti-CD3 Ab CD31^+^ between groups of different T cell subsets was determined by two-tailed ANOVA *P* = 0.7745, CD45RA^+^ *P* = 0.8195, CD4^+^ *P* = 0.9926. ns = not significant. n = number of individuals. (E) CD4^+^CD45RA^+^CD31^+^ T cells of CB (filled line), and adults (dashed line) treated with 0.5 μg/ml soluble anti-CD3 Ab and anti-CD28 Ab cross-linked with GAMIg in the presence (red) or absence (black) of 2 mM EGTA. One representative experiment out of three comparable experiments is shown. (F) STIM1 protein expression in CD4^+^ T cells of CB (black) and in naive CD31^+^ T cells from adults (gray) after stimulation using anti-CD3/anti-CD28 Ab. The densitometric analyses of the ratio of STIM1/α Tubulin are shown. Results are representative of at least two experiments.

Significant increase was found in the Ca^2+^ influx in response to 0.05 μg/ml anti-CD3 Ab without CD28 costimulation for CB compared with adult CD31^+^ naive T cells (Fig 4A adult black curve compared to Fig 4B CB black curve and Fig 4C, ANOVA Tukey-Kramer post-hoc test *P<*0.05, S7–S9 Tables).

In contrast however, in CB naive CD4^+^ T cells no significant difference was seen in any of the tested CD4 T cell subset between stimulation with soluble anti-CD3 Ab plus anti-CD28 Ab as compared to stimulation with any tested concentration of anti-CD3 Ab alone (Fig 4D, two-tailed ANOVA of difference; CD31^+^ *P* = 0.7745, CD45RA^+^ *P* = 0.8195, CD4^+^ *P* = 0.9926, S6 Table). Thus, in contrast to adult T cells [13], CB demonstrated no significant rise of the Ca^2+^ influx response after CD28 costimulation (Fig 2D).

### Ca^2+^ influx is not completely prevented by EGTA in CB naive CD31^+^ T cells

To analyze the dependency of Ca^2+^ on the availability of Ca^2+^ influx from intracellular Ca^2+^ stores, T cells were stimulated in the presence of extracellular Ca^2+^ or in the presence of the Ca^2+^ chelator ethylene glycol tetra acetic acid (EGTA) (Fig 4E). While the Ca^2+^ influx in adults was completely blocked by EGTA, it was only reduced by about 75% in CB indicating that CB T cells release more available intracellular Ca^2+^ than do adults.

One reason for this could be the availability of Ca^2+^ influx from intracellular Ca^2+^ stores or the use of ORAI CRAC channels of the plasma membrane, which leads to an influx of extracellular Ca^2+^ into the cytosol and activates CaN. The link between these intra- and extracellular stores is STIM1, which senses Ca^2+^ depletion in the ER (intracellular Ca^2+^ release) and then interacts with the ORAI CRAC channels to enhance the influx of extracellular Ca^2+^ into the cell. To evaluate whether the ER Ca^2+^ sensor STIM1 [37,38] was expressed in CB CD31^+^ naive T cells we examined STIM1 expression by Western blot analysis. Upon activation of adult and CB CD4^+^CD45RA^+^CD31^+^ T cells, STIM1 was strongly expressed in both types of T cells, independently of the stimulation (Fig 4D).

### Decreased Ca^2+^ influx response of CD4^+^ naive T cells in infants aged 3–5 months

**Fig 5:**
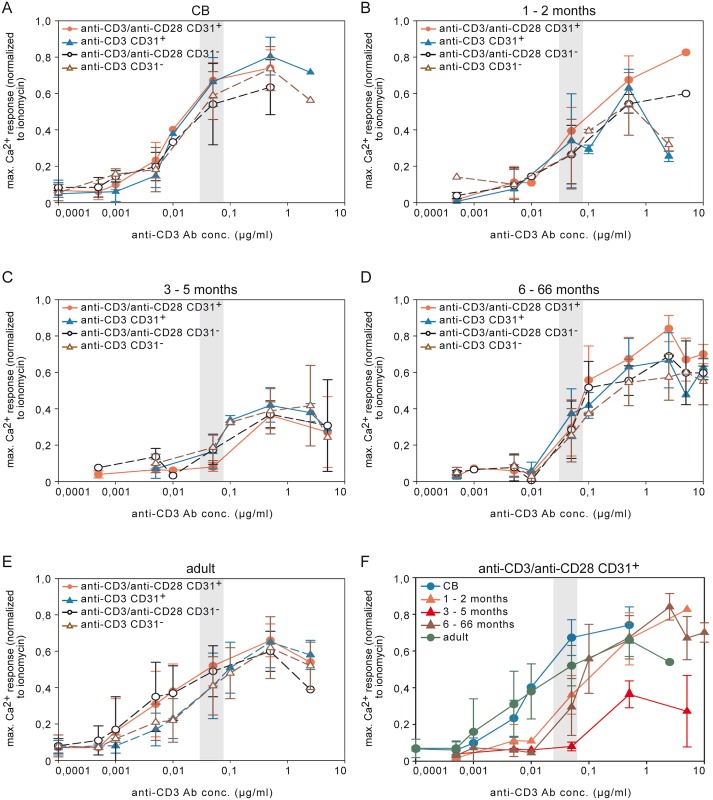
Dose response curves of the Ca^2+^ influx after TCR ligation in CB, infant/children, and adult for CD31^+^ and CD31^-^ naive T cells. Ca^2+^ mobilization in response to different anti-CD3 Ab concentrations plus 0.5 μg/ml soluble anti-CD28 Ab or with anti-CD3 alone (anti-CD28 Ab isotype) in combination with GAMIg measured using Indo-1AM staining and flow cytometry. Maximal Ca^2+^ influx response was normalized to the maximal Ca^2+^ influx of ionomycin treated samples and displayed as dose response curves for CD4^+^CD45RA^+^CD31^+^ and CD4^+^CD45RA^+^CD31^-^ naive T cells in (A) CB, (B) infant aged 1–2 months, (C) infant aged 3–5 months, (D) infant and children aged 6–66 months, and (E) adult. (F) Dose response curves by maximal Ca^2+^ influx normalized to the maximal Ca^2+^ influx ionomycin after anti-CD3 Ab TCR ligation and with anti-CD28 Ab stimulation displayed for CB, infant/children, and adult. The anti-CD3 Ab concentration of 0.05 μg/ml is marked with a gray bar. CD31^+^ = CD4^+^CD45RA^+^CD31^+^; CD31^-^ = CD4^+^CD45RA^+^CD31^-^.

To find the missing link between the modified functions in CB compared to adults we analyzed pediatric blood samples. These were classified into 3 groups: Based on the “hygiene hypothesis” [39,40] or rather “microbial deprivation hypothesis” [41,42] in correlation to the SIDS [43–45] we grouped 1–2 months (protection by maternal antibodies via passive maternal protection and breast-feeding, and constitution of the gut flora), 3–5 months (SIDS) and 6–66 months. In a first step we measured the Ca^2+^ influx responses of freshly prepared pediatric blood samples and generated Ca^2+^ influx dose-response curves in correlation to anti-CD3 Ab concentrations. Responses were normalized to maximal Ca^2+^ influx of ionomycin induced Ca^2+^ influx for a better comparison between the different groups (Fig 5A–5E).

**Fig 6:**
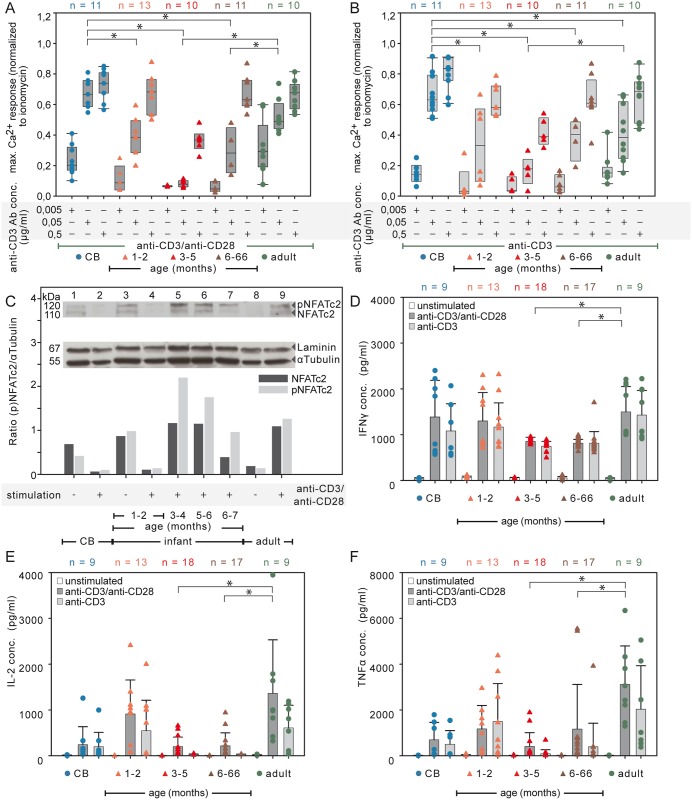
Age-dependent signatory Ca^2+^ influx and cytokine concentrations in supernatants of naive CD4^+^ T cells. (A-B) CD4^+^CD45RA^+^CD31^+^ T cells stimulated with anti-CD3 Ab as indicated either with costimulation by 0.5 μg/ml soluble anti-CD28 Ab (A, dark gray) or with the CD28 isotype control (B, light gray). Compiled data of box plots with scatter plots represent the Ca^2+^ influx response normalized to the maximal Ca^2+^ influx ionomycin response. Statistical significance between groups was determined by two tailed ANOVA Tukey-Kramer post-hoc test * *P<*0.05. n = number of individuals. (C) NFATc2 expression after anti-CD3 Ab plus anti-CD28 Ab engagement. The densitometric analyses of the immunoblots for the relative protein expression levels are shown as ratios of NFATc2 or pNFATc2 to αTubulin. Lysates from three different donors were pooled. Results are representative of at least two independent experiments. IFNγ (D), IL-2 (E), and TNFα (F) concentrations in the supernatants of unstimulated (white), of soluble anti-CD3 Ab plus anti-CD28 Ab stimulated (dark gray), and of TCR/CD3 stimulated alone (light gray, with anti-CD28 Ab isotype) of naive T cells using a Bio-Plex cytokine assay (Bio-Rad). The mean value and SD are indicated for five independent experiments (two tailed ANOVA Tukey-Kramer post-hoc test * *P<*0.05). n = number of individuals.

The Ca^2+^ influx of RTE T cells of CB compared with those of infants was already significantly different in response to anti-CD3 Ab cross-linking at a concentration of 0.05 μg/ml (Fig 5F gray bar; ANOVA followed by Tukey-Kramer post-hoc test *P<*0.05, S7–S9 Tables). Surprisingly, comparing the infants at the age of 3–5 months to the other age groups we uncovered a dramatic and significant reduction of the Ca^2+^ influx response to TCR engagement in all of the CD4^+^ T cell subpopulations examined (Fig 6A and 6B; S1 and S2 Figs; ANOVA followed by Tukey-Kramer post-hoc test *P<*0.05, S7–S9 Tables).

No CD28 enhanced Ca^2+^ influx comparable to that seen in adults was detected in CB and infants, including the youngest ones of 1–2 and 3–5 months (Fig 6A and 6B; ANOVA test of difference *P<*0.05, S6 Table). Furthermore there was a clear difference in the use of intracellular Ca^2+^ stores versus extracellular Ca^2+^ as shown by the addition EGTA to the medium. Ca^2+^ influx in stimulated adult T cells was abolished by EGTA but in infants 25% of the influx was insensitive to EGTA (data not shown). Thus, infant T cells aged 3–5 months are characterized by hyporeactivity due to limited Ca^2+^ influx with incomplete abolishment by EGTA for the Ca^2+^ influx level and with reduced T cell response to secondary CD28 stimulation as compared to adult T cells.

### Age-dependent NFAT expression of infants

CB CD4^+^ T cells expressed low constitutive levels of NFATc2 (Figs 3B and 3C and 6C, lane 1), as did unstimulated pooled peripheral blood CD4^+^ T cells of infants aged 1–2 months (Fig 6C, lane 3). However, after anti-CD3 Ab plus anti-CD28 Ab stimulation the NFATc2 expression was hardly detectable in pooled 1–2 months old infants (Fig 6C, lane 4). Of note, pooled T cells from infants older than two months, which were stimulated with anti-CD3 Ab plus anti-CD28 Ab showed a NFATc2 protein expression comparable or even higher than that of adults (Fig 6C, lane 5–7 and 9). In pooled CD4^+^ T cells of infants and of children there is a clear NFATc2 protein expression after TCR/CD28 stimulation. The only exception is for CD4^+^ T cells of pooled infants aged 1–2 months, which show a very low NFATc2 expression.

### Reduced cytokine response from T cells of infants 3–66 months old

The capacity of CD31^+^ naive T cells to induce the expression of cytokines such as IL-2 and TNFα was analyzed after 24 h stimulation [23,24] and, as a control for T cell functionality, secreted IFNγ was measured after 48 h stimulation [25]. IFNγ was induced in the naive T cells of all age groups (Fig 6D). As a control for CD28 costimulatory effects, the adult CD31^+^ naive T cells significantly increased their IL-2 and TNFα amounts in supernatants after anti-CD3 Ab plus anti-CD28 Ab cross-linking compared with anti-CD3 Ab stimulation alone (Fig 6E and 6F; ANOVA test of difference *P<*0.05, S10–S12 Tables). Infants and children aged 3–66 months showed significantly less IFNγ in the supernatants compared with adults (Fig 6D, two tailed ANOVA Tukey-Kramer post-hoc test * *P<*0.05, S13–S16 Tables). Substantially stronger differential age effects were observed for IL-2 and TNFα. The concentrations of these cytokines were dramatically lower in infants and children aged 3–66 months compared to adults (Fig 6E and 6F, two tailed ANOVA Tukey-Kramer post-hoc test * *P<*0.05, S13–S16 Tables). Unexpectedly, there was a marked increase in the concentration of IL-2 in supernatants of activated naive T cells of infants aged 1–2 months, which was almost as high as the levels seen in adults and substantially higher than in either the CB or in the infants of 3–66 months.

In addition, CD28 costimulatory effects did not reach significance in the RTE T cells from CB and infants aged 1–2 months in contrast to adults (Fig 6D–6F). Thus, a significant age-related cytokine response of naive T cells over the first 3 years of life was demonstrated.

## Discussion

Our findings represent the first observations of distinct TCR and/or CD28 engagement effects on the activation of early signaling pathways in naive T cells during the first 66 months of life compared to the effects seen in adult RTE T cells. Naive CD4^+^ T cells in CB and infants up to the age of 2 months showed a reduced anti-CD28 Ab triggered TCR response compared to that seen in adults. Using key molecular events of TCR- and CD28-induced signaling such as Ca^2+^ influx, NFAT expression, phosphorylation and translocation into the nucleus, activation of ERK and IL-2, we show that some of the known main pathways are available for T cell responses but that they are utilized in a strictly age-dependent manner. These findings demonstrate that age-related differences in Ca^2+^ influx are a key mechanism that can limit NFAT translocation and IL-2 production in naive CD4^+^ T cells.

The most reliable way to evaluate the signaling capacity of T cells is to compare RTEs between age groups because thymic output in humans decreases with age in some individuals dramatically (see SD of adults in Fig 1). Our results and previously published data [2] describes a well-defined RTE subpopulation of naive T cells. Although effector memory cells may re-express CD45RA [46], this fraction is only about 5% and CD45RACD31^+^ and CD45RACD31^-^ behave similarly in terms of IL-2 production [47]. In addition, analysis of the CD31^+^ cell frequencies supports the notion that the largest fraction are naive T cells in infants, since the frequency of CD31^+^CD45RA^+^ cells does not significantly change during childhood (Fig 1).

**Fig 7:**
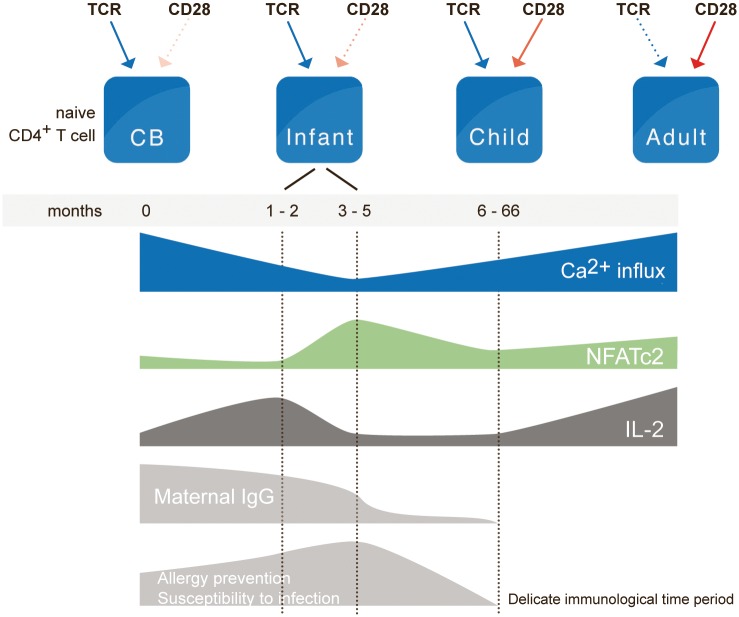
Visualizing the occurrence of age-dependent characteristics of T cell activation. The requirement of activation by the TCR/CD3 complex is less dependent on costimulation by CD28 in CB naive CD4^+^ T cells compared to that seen in adult cells. The intensity of Ca^2+^ influx, NFATc2 expression and IL-2 response are all age-dependent. A dramatic shift is seen in the naive T cell response at the age of 2 months. The cells’ capacity to produce high amounts of IL-2 is suddenly abrogated. Ca^2+^ influx declines to the lowest values observed in life. At 6 months of age, the IL-2 response starts to improve slowly. This “reprogramming” of T cells takes place as the passively transferred maternal Abs in the infant are beginning to decline. Limited T-cell responses likely contribute to the high risk of infants to suffer from infections and infection-related pathologies such as Sepsis and SIDS [43,44] during the first months of life.

Our data demonstrate age-dependent differences in the magnitude of Ca^2+^ influx in RTE T cells in response to TCR stimulation. An increased basal Ca^2+^ level increases cytokine production capacity of T cells [29], and the amplitude and duration of the intracellular Ca^2+^ ion signal in B and T cells controls differential activation of the pro-inflammatory transcriptional regulators [20,48,49]. Ca^2+^ influx is thus likely to be the key event that controls the age-dependent IL-2 response. Whereas NF-κB and JNK (AP1) are selectively activated by a large transient intracellular Ca^2+^ rise, NFAT in contrast is activated by a low sustained Ca^2+^ plateau [20]. Using defined RTE populations, our main findings are that the Ca^2+^ influx signal is characteristic for particular ages and that it correlates partially with IL-2. Thus, an intrinsic, signatory Ca^2+^ influx likely exists at all ages and directs the strength of the T cell response. Only CB RTEs differed in that they showed high Ca^2+^ influx similar to RTEs but with little IL-2 in their supernatants (Fig 7).

We found differences at the Ca^2+^ influx signal not only in terms of the extent of the signal but also in terms of the utilization of intra- or extracellular Ca^2+^ stores. While the Ca^2+^ influx in adults was completely blocked by EGTA, it was only reduced in CB (Fig 4E) and infants aged 1–2 months. As described before [50] Ca^2+^ influx will be generated from the three major cellular compartments: cytosol, ER, mitochondria as well as the extracellular space. We suggest that in CB and in infants there are different basal Ca^2+^ dynamics. That is to say that the T cells sustain elevated cytoplasmic Ca^2+^ levels for gene transcription, by balancing store-operated Ca^2+^ entry (SOCE) through the plasma membrane and Ca^2+^ buffering by the mitochondria. We suggest that because of this “higher profligate” Ca^2+^ response in CB there is also inadequate feedback inhibition by STIM1, which results in inadequate balancing of SOCE and Ca^2+^ clearance. The recently described inhibition of Ca^2+^ clearance due to STIM1 association with PMCA4 [38,51], might explain why extracellular Ca^2+^ influx is not required. We found equally strong expression of STIM1 [37,52] in CB and adult naive CD4^+^ T cells independent of the stimulation (Fig 4F) showing that STIM1 protein is not limiting. Nevertheless, its interaction with PMCA4 could well make the difference, and provide an explanation for the lack of inhibition of Ca^2+^ clearance and the low NFATc2 in CB.

**Fig 8:**
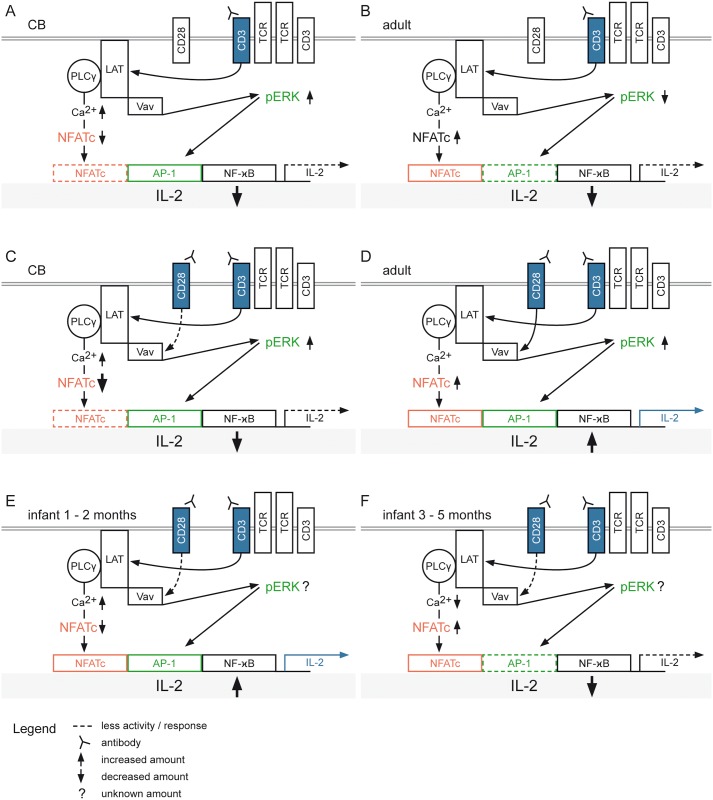
Schematic model for the molecular basis of age-dependent T cell activation. Engagement of T cell receptors (TCR) and costimulatory CD28 receptors (blue) by soluble anti-CD3 Ab and/or anti-CD28 Ab stimulation promotes signaling cascades of adaptor proteins (LAT) and kinases (ERK). These trigger signal transduction pathways resulting in the activation of the transcription factors NFATc (red), NF-κB (white) and the association of AP-1 with cFos/cJun (green). These transcription factors cooperate with each other during the activation of the IL-2 promoter. Shown are the schematic molecular responses of (A) CB and (B) adult with soluble anti-CD3 Ab stimulation alone or together with costimulation by anti-CD28 Ab (blue) for (C) CB and (D) adult. A hypothetical molecular mechanism for infants aged 1–2 months (E) or 3–5 months (F) after TCR/CD3 engagement plus CD28 costimulation with unknown pErk response.

Based on our results, we highlight the following key observations that refine our understanding of molecular mechanisms that distinguish T cell activation in CB vs. adults: 1) The Ca^2+^ influx in CB samples was less dependent on extracellular stores than in adults 2) NFAT is expressed to a lesser extent in CB compared to adult under the same conditions 3) The pERK level is higher in CB than in adults 4) IL-2 secretion is lower in CB than in adults and is less dependent on CD28 mediated costimulation. On a molecular basis we propose the following scheme (Fig 8):

The reduced NFAT expression in CB is based on the incomplete EGTA inhibited Ca^2+^ influx, which is due to increased Ca^2+^ clearance over with the plasma membrane PMCA4 (Ca^2+^/ATPase 4 Ca^2+^ pump) as described elsewhere [38,51,53]. In adult cells, EGTA completely inhibits the Ca^2+^ influx so that neither STIM1 mediated Ca^2+^ clearance nor NFAT activity are profoundly attenuated In CB, NFAT expression is the limiting factor for IL-2 promoter activation (Fig 8A). In adult cells, the limiting factor is the Raf-MEK-ERK pathway, because anti-CD3 Ab leads to a low degree of pERK and IL-2 production, but after anti-CD3 Ab and anti-CD28 Ab stimulation there is increased pERK, which results in increased IL-2 production (Fig 8B and 8D). A higher concentration of anti-CD3 Ab stimulation can compensate for CD28 (Fig 8D). For CB, these differences do not occur. In terms of the NFAT- and Raf-MEK-ERK-pathways the infants represent intermediate states between CB and adult profiles. In infants 1–2 months, NFAT expression is clearly lower compared to that seen in infants aged 3–5 months. The Ca^2+^ influx signal is still not completely inhibited by EGTA, but not quite as strongly as in CB and consequently we found that reduced NFAT expression was still sufficient to produce more IL-2 than in CB (Fig 8E). For infants aged 3–5 months we have a decreased Ca^2+^ influx signal, which leads to a strong NFATc2 expression but the IL-2 expression is decreased. By applying our model to the balance of the NFAT- and Raf-MEK-ERK-pathways of infants, we propose that the activation/expression of pERK is likely decreased since the IL-2 response is increased by anti-CD28 Ab stimulatory function (Fig 8F).

The decreased IL-2 production in CB might be explained by internalization of the TCR proportional to the strength (high Ca^2+^ influx) of the initial antigen recognition [54]. Our finding showed that with high anti-CD3 Ab concentrations the NFATc2 expression was increased in adult RTEs, but in CB cells NFATc2 expression was only initiated at low anti-CD3 Ab concentrations of 5 μg/l (Fig 3B and S3 Fig). NFATc2 expression was unambiguously detected in activated CB T cells in contrast to previously published data [6]. However, the phosphorylated and unphosphorylated NFATc2 status differed from CB to adult, whereby strong NFATc2 expression was found when TCR engagement was supplemented with CD28 engagement in adults. Our analyses, using different signaling strengths and costimulation, suggest that previous failures to detect NFATc2 in CB naive T cells were likely due to the use of higher concentrations of anti-CD3 Ab and hence to a signaling strength that was inappropriate for this particular type of cell. The demonstrated inefficient translocation of NFATc2, on the other hand, was not due to suboptimal activation of the cells because pERK was strongly induced at the same time as has been described in earlier studies [32].

It has been proposed [55], that Foxp3^+^ regulatory T cells express NFAT constitutively in the nucleus independent of CaN activity, though in human it has been reported that there is no CaN independent nuclear localization of NFAT [56]. Our data suggest that in human CB there is no nuclear localization of NFAT independent of CaN. There is, however, probably constitutive cytoplasmic localization of NFAT in CB. Therefore, NFATc2 expression in the nucleoplasm of CB T cells is almost abolished, but it remains higher after CsA treatment in the cytoplasm compared to what is seen in adult cells [36]. In resting T cells NFATc is expressed highly phosphorylated and localized in the cytosol. Upon stimulation and subsequent calcium mobilization activated CaN dephosphorylates NFATc in the regulatory region [36], leading to its nuclear translocation.

However, divergent stimulation scenarios are possible, so that TCR and CD28 may activate the transcription factor NF-κB in T cells via distinct adaptor signaling complexes [57]. Strikingly, CB CD31^+^ naive T cells induced phosphorylation of ERK to an even higher extent than did adult RTE T cells. By extension, ERK dependent events may be promoted in CB CD31^+^ naive T cells, whereas NFAT dependent events are kept to a minimum.

At different time periods of infancy, RTE show varying responses to CD28 costimulation. The anti-CD28 Ab response was reduced up to two months of age and this likely leads to the weak and transient Ca^2+^ influx. This influx may be insufficient to achieve a threshold for NFAT activation, as observed in adult cells at low CD3 concentrations in absence of CD28 costimulation [13]. Our findings demonstrate a correlation between the sensitivity to CD28 costimulation and increasing infant age. This observation provides insight into the appropriate age to start costimulation-based immunotherapy [58]. From an evolutionary perspective, we hypothesize that the non-adult-like Ca^2+^ response might be a relic; using the CD28 Superagonist Ab in monkeys, almost no Ca^2+^ influx was shown for T cells of rhesus and cynomolgus monkeys, but a dramatically prolonged Ca^2+^ influx was observed in adult human T cells [59–62]. It is possible that the human CB and infant immunological equipment is configured more like ontogenetically related species, such as rhesus and cynomolgus monkeys, within the first two months of life and does not switch on the CD28 response observed in adult humans until after this period. We will be interested to learn whether age-dependency is related to the time of antigen exposure or simply to the age of the organism, as shown for the B-cell receptor (BCR) repertoire in infants [63].

Our data demonstrate that infants’ T cells are unambiguously immunologically competent. In particular, their IFNγ response is similar to that of adult naive T cells, although at 3–66 months it is somewhat lower. One reason for keeping IFNγ up might be the need for an immediate defense against bacterial and viral infections. Alternatively, an isolated IFNγ response in CB and infants could be an innate-like function of T cells as has been described recently under other circumstances [64]. Though the IFNγ response of the infant T cells is similar to adult cells, the IL-2 response is dramatically different, as the CB cells respond much less vigorously than do adult cells.

Only a few weeks after birth, the cytokine responses of naive infant T cells (1–2 months) are as strong as those of adults and correlate well with their Ca^2+^ influx. Thus, CB and infant T cells are similar in their signal transduction events proximal to the TCR signal, however, their IL-2 response is different. One explanation proposed was that human CB naive CD4^+^ T cells have enhanced activation-dependent signaling which is regulated by the microRNA miR-181a [32]. Alternatively, the preferential development of Treg cells by the fetus, leading to a “layered” immune system, stops abruptly after birth [65] and this might explain the disparity in the IL-2 responses. In addition immunosuppressive factors such as TGFβ, IL-10, or IDO are produced by the placenta during birth or even during the fetal period and these may dampen the effector functions of CB T cells [66,67]. This difference between CB T cells on the day of birth and T cells only few days and weeks later might be important to consider for many studies using CB T cells.

For the first two months of life, high IL-2 responses would be expected to lead to the generation of T effector cells, though these rapidly proliferating cells might progress to terminally differentiated T cells, which eventually undergo apoptosis [68–71]. Because these infant T cells do not respond to CD28 costimulation, they are not likely to sustain their responses for long, as has been described for adult murine CD28 deficient T cells [72,73]. Just 2 months later, T cells respond to activation with very limited amounts of IL-2. This strongly reduced IL-2 production in CD4^+^ T cells would not only limit their own expansion but would also reduce help provided to CD8^+^ T cells—or at least prevent them from developing their full killing potential [74]. Reduced T cell numbers and help would also lead to reduced memory formation. Many yet unknown antigens from dietary compounds, commensal bacteria, and environmental sources flood the immune system in the first month of life, so the prevention of immune memory formation in this period would appear to be a prudent strategy. Our work thus may help us understand the paradoxical observation that T cell responses in infants are strong but are difficult to induce [3,6,8,9,22–25,75–78].

Our data show a dramatic shift in the naive T cell response at the age of 2–3 months. The cells’ capacity to produce high amounts of IL-2 and TNFα is suddenly abrogated. Ca^2+^ influx declines to the lowest values observed in life while NFAT phosphorylation rises. This “reprogramming” of T cells takes place as the passively transferred maternal Abs in the infant are still present but are beginning to decline; furthermore, in this window of time, sudden infant death syndrome (SIDS) peaks [43,44]. Given that SIDS is related to infections [79,80], the reprogramming of T cells may well be one—if not indeed the decisive factor—for the causes of SIDS. Though factors such as hyperthermia, genetic factors, infections and probably others are doubtless also involved, an immune challenge in this particular age window is bound to be more life threatening than at other ages. Finally, we propose that the monitored age-related signatory Ca^2+^ influx dictates the decision-making of the adaptive immune system in favor of preventing responses. This finding demands a re-evaluation of the optimal time for pediatric vaccination to achieve lifelong protection.

## Supporting Information

S1 Fig Dose response curves of the Ca^2+^ influx after TCR ligation in CB, infant/children, and adult for CD45RA^+^ and CD45RA^-^ naive T cells. Ca^2+^ mobilization in response to different anti-CD3 Ab concentrations plus 0.5 μg/ml soluble anti-CD28 Ab or with anti-CD3 alone (anti-CD28 Ab isotype) in combination with GAMIg measured using Indo-1AM staining and flow cytometry. Maximal Ca^2+^ influx response was normalized to the maximal Ca^2+^ influx of ionomycin treated samples and displayed as dose response curves for CD4CD45RA^+^ and CD4CD45RA^-^ naive T cells in (A) CB, (B) infant aged 1–2 months, (C) infant aged 3–5 months, (D) infant and children aged 6–66 months, and (E) adult. (F) Dose response curves by maximal Ca^2+^ influx normalized to the maximal Ca^2+^ influx ionomycin after anti-CD3 Ab TCR ligation and with anti-CD28 Ab stimulation displayed for CB, infant/children, and adult. The anti-CD3 Ab concentration of 0.05 μg/ml is marked with a gray bar. CDRA^+^ = CD45RA^+^ = CD4^+^CD45RA^+^; CDRA^-^ = CD45RA^-^ = CD4^+^CD45RA^-^.
(TIF)

S2 Fig Dose response curves of the Ca^2+^ influx after TCR ligation in CB, infant/children, and adult for CD4^+^ and CD4^-^ naive T cells. Ca^2+^ mobilization in response to different anti-CD3 Ab concentrations plus 0.5 μg/ml soluble anti-CD28 Ab or with anti-CD3 alone (anti-CD28 Ab isotype) in combination with GAMIg measured using Indo-1AM staining and flow cytometry. Maximal Ca^2+^ influx response was normalized to the maximal Ca^2+^ influx of ionomycin treated samples and displayed as dose response curves for CD4^+^ and CD4^-^ naive T cells in (A) CB, (B) infant aged 1–2 months, (C) infant aged 3–5 months, (D) infant and children aged 6–66 months, and (E) adult. (F) Dose response curves by maximal Ca^2+^ influx normalized to the maximal Ca^2+^ influx ionomycin after anti-CD3 Ab TCR ligation and with anti-CD28 Ab stimulation displayed for CB, infant/children, and adult. The anti-CD3 Ab concentration of 0.05 μg/ml is marked with a gray bar.
(TIF)

S3 Fig Original immunoblot of the experiment shown in Fig 2E. Increased levels of NFATc2 protein expression in response to anti-CD3/anti-CD28 Ab stimulation in naive T cells (to the right). The immunoblot detection of NFATc2 or pNFATc2 to αTubulin is shown for naive CD31^+^ or CD31^-^ T cells of adults stimulated with anti-CD3 Ab in combination with soluble anti-CD28 Ab or anti-CD28 Ab isotype. Laminin and αTubulin were used as loading controls (to the left). Results are representative of at least two experiments. unstim. = unstimulated.
(TIF)

S4 Fig Original immunoblot of the experiment shown in Fig 3A. Protein expression by Western blot of ERK1/2 (left top) and phosphorylated ERK1/2Tyr202/ Tyr204 (pERK1/2, right top) in naive CD4^+^ T cells of CB as well as for naive CD31^+^ adult T cells. Data are representative of two independent experiments. αTubulin was used as a loading control of ERK1/2 (left bottom) and phosphorylated ERK1/ (pERK1/2, right bottom).
(TIF)

S5 Fig Original immunoblot of the experiment shown in Fig 3B. Whole cell protein extract of NFATc2 and phosphorylated NFATc2 (pNFATc2) in CB naive CD4^+^ T cells and adult naive CD31^+^ T cells under different stimulation conditions (to the right). Lysates from three different donors were pooled. Data are representative of at least three independent experiments. αTubulin was used as a loading control (to the left).
(TIF)

S6 Fig Original immunoblot of the experiment shown in Fig 3C. The NFATc2 protein expression in cytoplasm or nucleoplasm in naive CD4^+^ T cells of CB was detected and the phosphorylated (pNFATc2) and dephosphorylated (NFATc2) forms quantified. Cells were stimulated as indicated in the presence or absence of cyclosporin A (CsA). One representative experiment out of two comparable experiments is shown. unstim. = unstimulated.
(TIF)

S7 Fig Original immunoblot of the experiment shown in Fig 3D. The NFATc2 protein expression in cytoplasm or nucleoplasm in CD31^+^ naive T cells of adult was detected and the phosphorylated (pNFATc2) and dephosphorylated (NFATc2) forms quantified. Cells were stimulated as indicated in the presence or absence of cyclosporin A (CsA). One representative experiment out of two comparable experiments is shown. unstim. = unstimulated.
(TIF)

S8 Fig Original immunoblot of the experiment shown in Fig 4F. STIM1 protein expression in CD4^+^ T cells in naive CD31^+^ T cells from adults and CB after stimulation using anti-CD3/anti-CD28 Ab (to the right). Results are representative of at least two experiments. αTubulin was used as a loading control (to the left).
(TIF)

S9 Fig Original immunoblot of the experiment shown in Fig 6C. NFATc2 expression after anti-CD3 Ab plus anti-CD28 Ab engagement. The immunoblots are shown of NFATc2 and phosphorylated (pNFATc2) (to the right) and of αTubulin. Laminin and αTubulin was used as a loading control (to the left). Lysates from three different donors were pooled. Results are representative of at least two independent experiments.
(TIF)

S1 Table Relative frequencies of lymphocyte subpopulation in dependent on age. (DOCX)

S2 Table Summarized Analysis of variance (ANOVA) assessment for frequencies. (DOCX)

S3 Table Concrete single analysis of ANOVA for frequencies CD45RA^+^ among CD4^+^ T cells data for 5 groups of infants (CB, infants 1–2 mo, infants 3–5 mo, infants 6–66 mo, adult). (DOCX)

S4 Table Normalized Ca^2+^ influx data with means and SD. (DOCX)

S5 Table Summary of significant differences of two-tailed ANOVA of differences in the Ca^2+^ influx responses for the difference of subset of T cell for 3 different anti-CD3 Ab concentration (0.005 μg/ml, 0.05 μg/ml and 0.5 μg/ml) with or without anti-CD28 Ab stimulation of adult. (DOCX)

S6 Table Summary of two-tailed ANOVA of difference the Ca^2+^ influx responses for the difference of anti-CD3/CD28 Ab with anti-CD3 Ab. (DOCX)

S7 Table Summary of Analysis of variance (ANOVA) assessment for Ca^2+^ influx data for the different subset of T cell type for 3 different anti-CD3 Ab concentrations (0.005 μg/ml, 0.05 μg/ml and 0.5 μg/ml) with (+) or without (-) anti-CD28 Ab stimulation of adult. (DOCX)

S8 Table Summary of significant differences of the Tukey test in the Ca^2+^ influx responses (*) of infants (ages in months: 1–2, 3–5 or 6–66) compared to CB and adults of the CD4^+^ T cells subgroups depend on anti-CD28 Ab stimulation. (DOCX)

S9 Table Concrete single analysis of Analysis of variance (ANOVA) assessment for Ca^2+^ influx data for the different subset of T cell type for 5 groups of individuals (CB, infants 1–2 months, infants 3–5 months, infants 6–66 months, adult) and for 3 different Anti-CD3 Ab concentrations (0.005 μg/ml, 0.05 μg/ml and 0.5 μg/ml). (DOCX)

S10 Table Cytokine productions of naive T cells after TCR-ligation. (DOCX)

S11 Table Summary of ANOVA assessment for cytokine production for differences of stimulation (unstimulated, anti-CD3/CD28 and anti-CD3) for 5 groups of individual. (DOCX)

S12 Table Summary of significant differences of the two-tailed ANOVA of differences for cytokine production of stimulation (unstimulated, anti-CD3/CD28 and anti-CD3) for 5 groups with infants compared to CB and adults of the CD4^+^ CD45RA^+^ CD31^+^ T cells. (DOCX)

S13 Table Concrete single analysis of ANOVA assessment for cytokine for 5 groups of individual (CB, infants 1–2 mo, infants 3–5 mo, infants 6–66 mo, adult) differences between stimulation of anti-CD3/CD28 Ab group with Anti-CD3 Ab group. (DOCX)

S14 Table Summary of ANOVA assessment for cytokine production for 5 groups of individual (CB, infants 1–2 mo, infants 3–5 mo, infants 6–66 mo, adult). (DOCX)

S15 Table Summary of significant differences of the Tukey test in the cytokine production (*) of infants (ages in months: 1–2, 3–5 or 6–66) compared to CB and adults of the CD4^+^CD45RA^+^CD31^+^ T cells on stimulation (unstimulated, anti-CD3/CD28 Ab, anti-CD3 Ab). (DOCX)

S16 Table Concrete single analysis of ANOVA assessment for cytokine between 5 groups of individual (CB, infants 1–2 mo, infants 3–5 mo, infants 6–66 mo, adult) under three different stimulation (unstimulated, anti-CD3/CD28 Ab, Anti-CD3 Ab group). (DOCX)

S17 Table Summary of significant differences of the two-tailed ANOVA of differences for cytokine production of stimulation for adults of the CD4^+^CD45RA^+^CD31^+^ T cells (CD31^+^) subgroups compared to adult the CD4^+^CD45RA^+^CD31^-^ T cells (CD31^-^). (DOCX)
