# Supplementary figures and images for: T Cells of Infants Are Mature, but Hyporeactive Due to Limited Ca2+ Influx
Source: PLoS One. 2016 Nov 28;11(11):e0166633. doi: 10.1371/journal.pone.0166633 (PMC5125607; doi:10.1371/journal.pone.0166633)

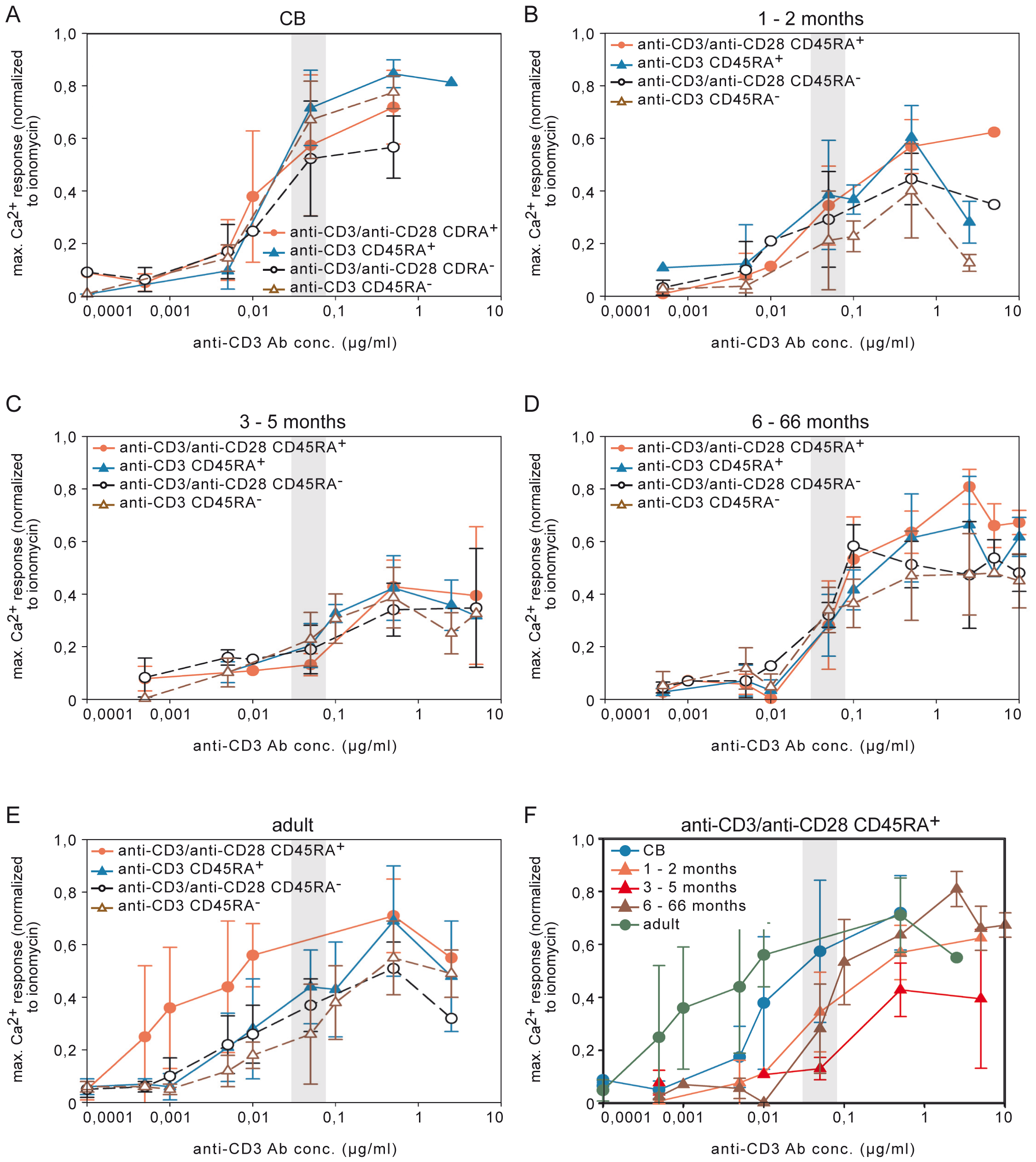

Supplement: S1 Fig — Ca2+ mobilization in response to different anti-CD3 Ab concentrations plus 0.5 μg/ml soluble anti-CD28 Ab or with anti-CD3 alone (anti-CD28 Ab isotype) in combination with GAMIg measured using Indo-1AM staining and flow cytometry. Maximal Ca2+ influx response was normalized to the maximal Ca2+ influx of ionomycin treated samples and displayed as dose response curves for CD4CD45RA+ and CD4CD45RA- naive T cells in (A) CB, (B) infant aged 1–2 months, (C) infant aged 3–5 months, (D) infant and children aged 6–66 months, and (E) adult. (F) Dose response curves by maximal Ca2+ influx normalized to the maximal Ca2+ influx ionomycin after anti-CD3 Ab TCR ligation and with anti-CD28 Ab stimulation displayed for CB, infant/children, and adult. The anti-CD3 Ab concentration of 0.05 μg/ml is marked with a gray bar. CDRA+ = CD45RA+ = CD4+CD45RA+; CDRA- = CD45RA- = CD4+CD45RA-. (TIF) [file pone.0166633.s001.tif]

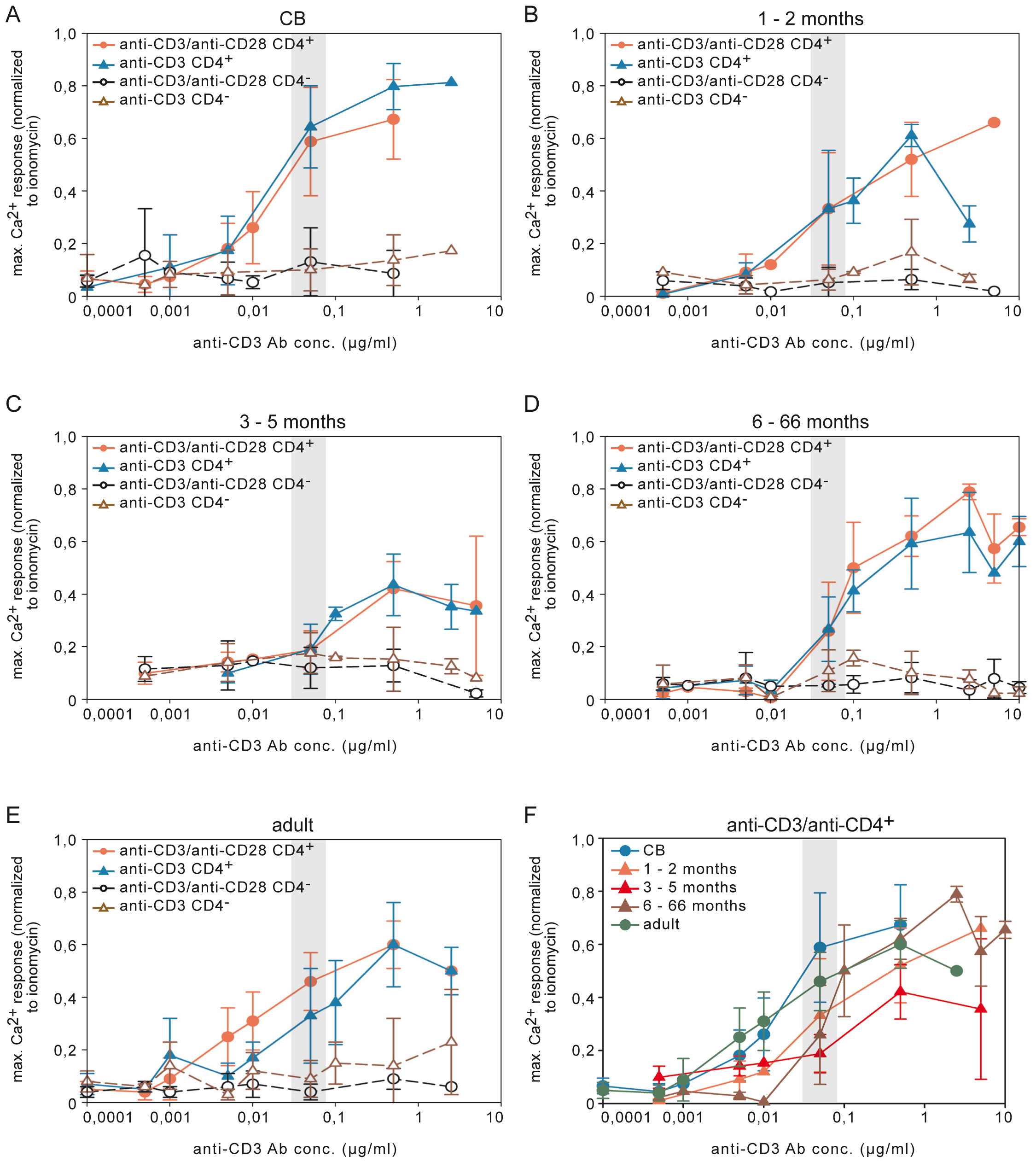

Supplement: S2 Fig — Ca2+ mobilization in response to different anti-CD3 Ab concentrations plus 0.5 μg/ml soluble anti-CD28 Ab or with anti-CD3 alone (anti-CD28 Ab isotype) in combination with GAMIg measured using Indo-1AM staining and flow cytometry. Maximal Ca2+ influx response was normalized to the maximal Ca2+ influx of ionomycin treated samples and displayed as dose response curves for CD4+ and CD4- naive T cells in (A) CB, (B) infant aged 1–2 months, (C) infant aged 3–5 months, (D) infant and children aged 6–66 months, and (E) adult. (F) Dose response curves by maximal Ca2+ influx normalized to the maximal Ca2+ influx ionomycin after anti-CD3 Ab TCR ligation and with anti-CD28 Ab stimulation displayed for CB, infant/children, and adult. The anti-CD3 Ab concentration of 0.05 μg/ml is marked with a gray bar. (TIF) [file pone.0166633.s002.tif]

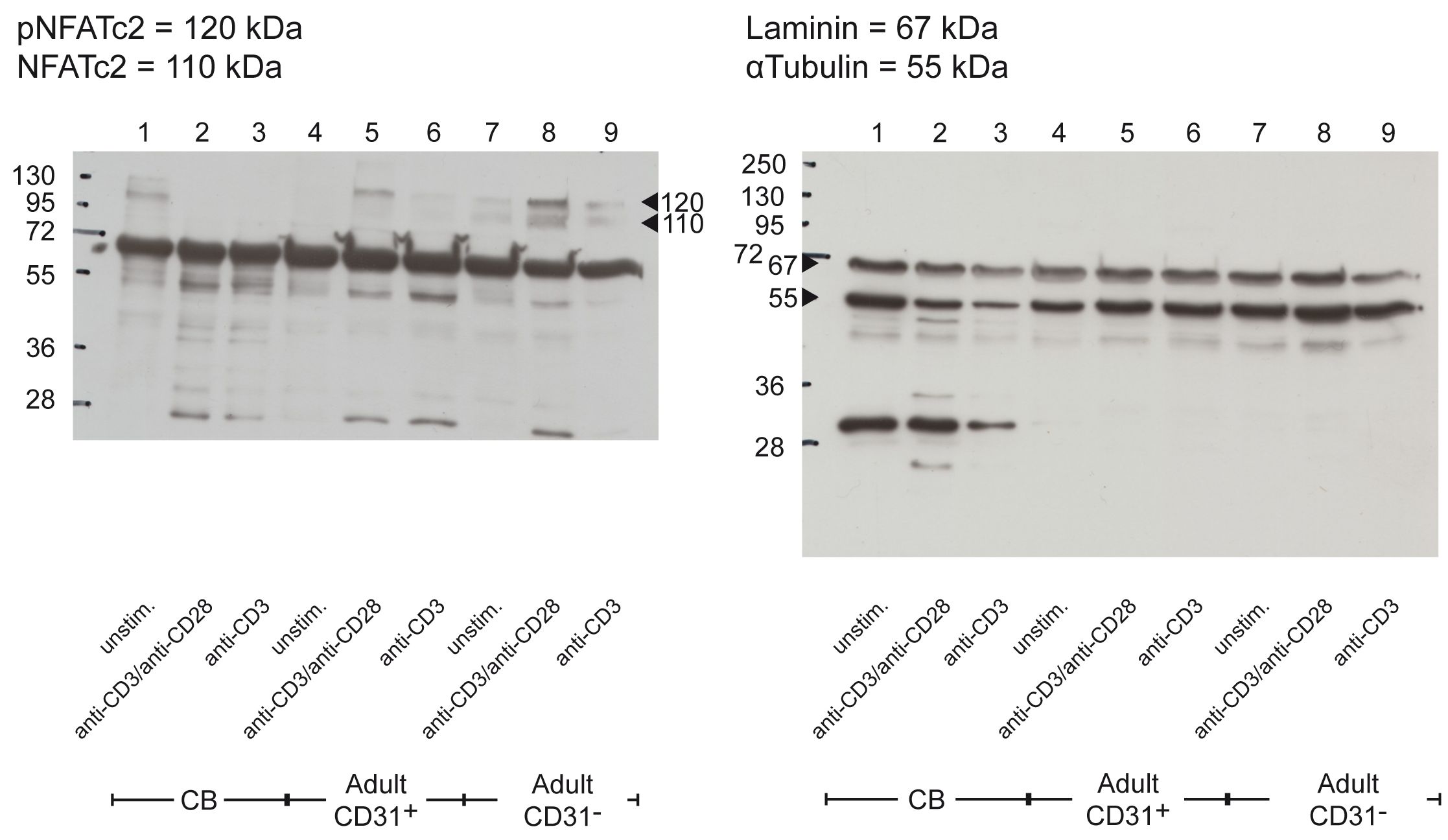

Supplement: S3 Fig — Increased levels of NFATc2 protein expression in response to anti-CD3/anti-CD28 Ab stimulation in naive T cells (to the right). The immunoblot detection of NFATc2 or pNFATc2 to αTubulin is shown for naive CD31+ or CD31- T cells of adults stimulated with anti-CD3 Ab in combination with soluble anti-CD28 Ab or anti-CD28 Ab isotype. Laminin and αTubulin were used as loading controls (to the left). Results are representative of at least two experiments. unstim. = unstimulated. (TIF) [file pone.0166633.s003.tif]

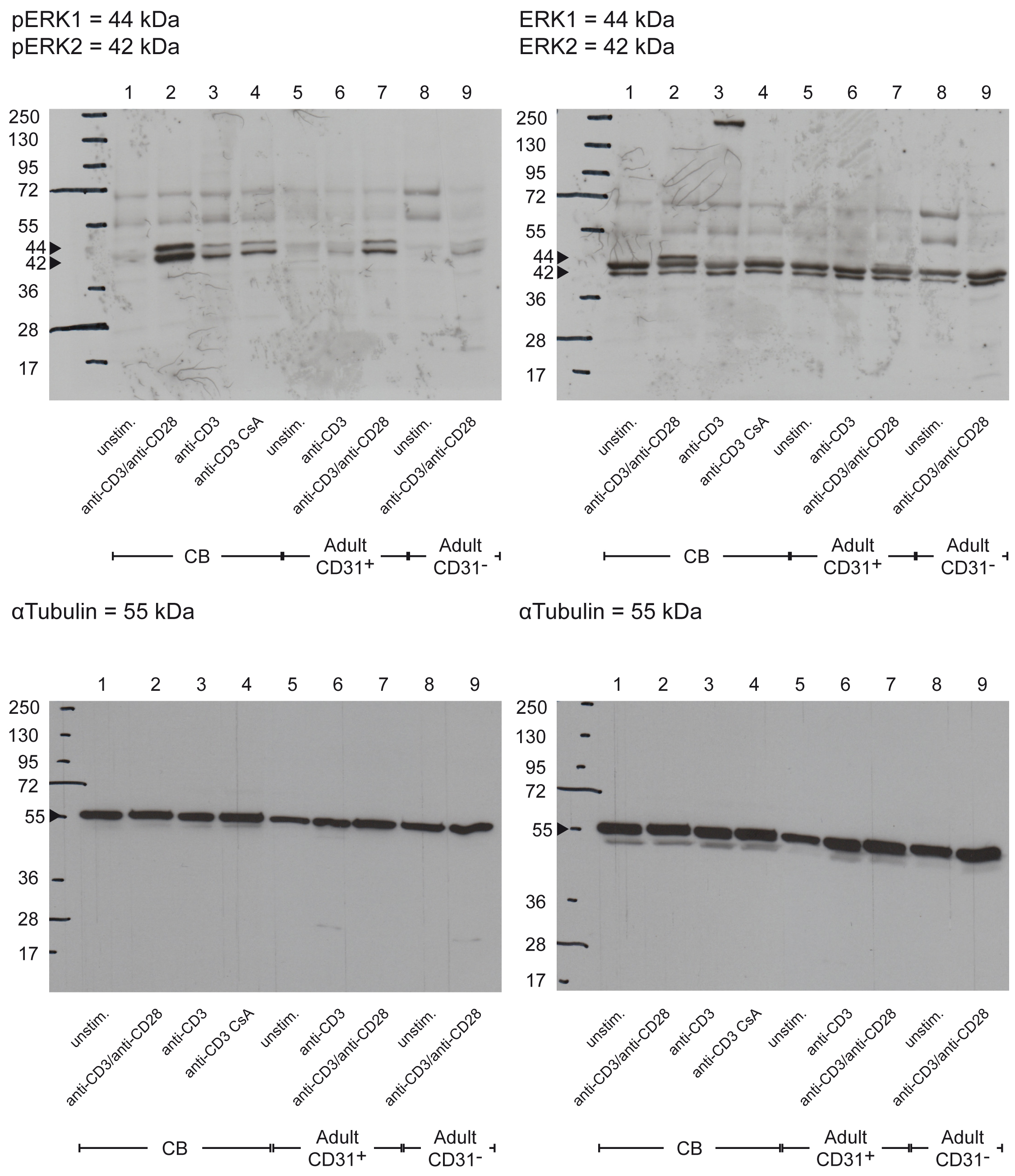

Supplement: S4 Fig — Protein expression by Western blot of ERK1/2 (left top) and phosphorylated ERK1/2Tyr202/ Tyr204 (pERK1/2, right top) in naive CD4+ T cells of CB as well as for naive CD31+ adult T cells. Data are representative of two independent experiments. αTubulin was used as a loading control of ERK1/2 (left bottom) and phosphorylated ERK1/ (pERK1/2, right bottom). (TIF) [file pone.0166633.s004.tif]

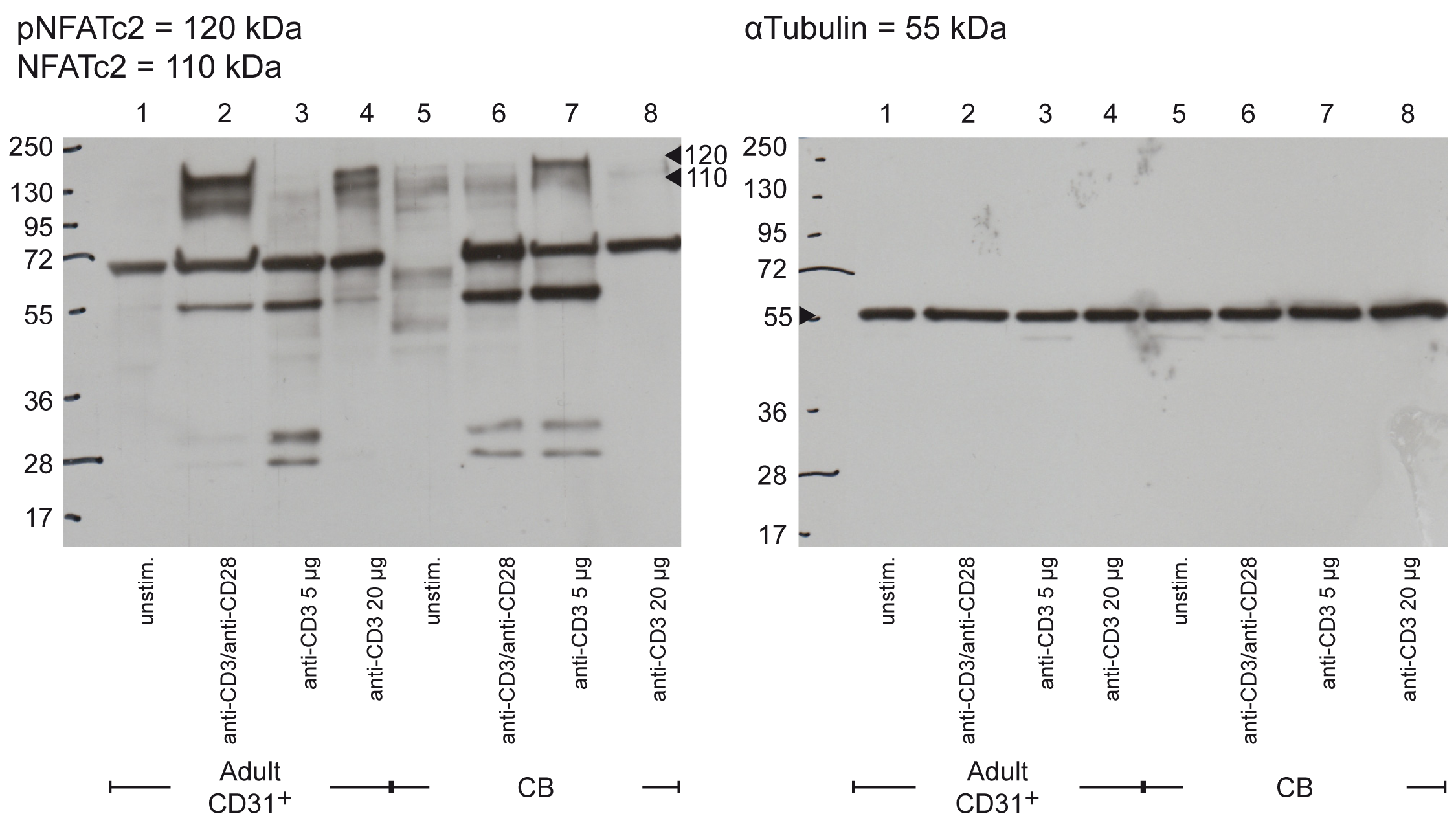

Supplement: S5 Fig — Whole cell protein extract of NFATc2 and phosphorylated NFATc2 (pNFATc2) in CB naive CD4+ T cells and adult naive CD31+ T cells under different stimulation conditions (to the right). Lysates from three different donors were pooled. Data are representative of at least three independent experiments. αTubulin was used as a loading control (to the left). (TIF) [file pone.0166633.s005.tif]

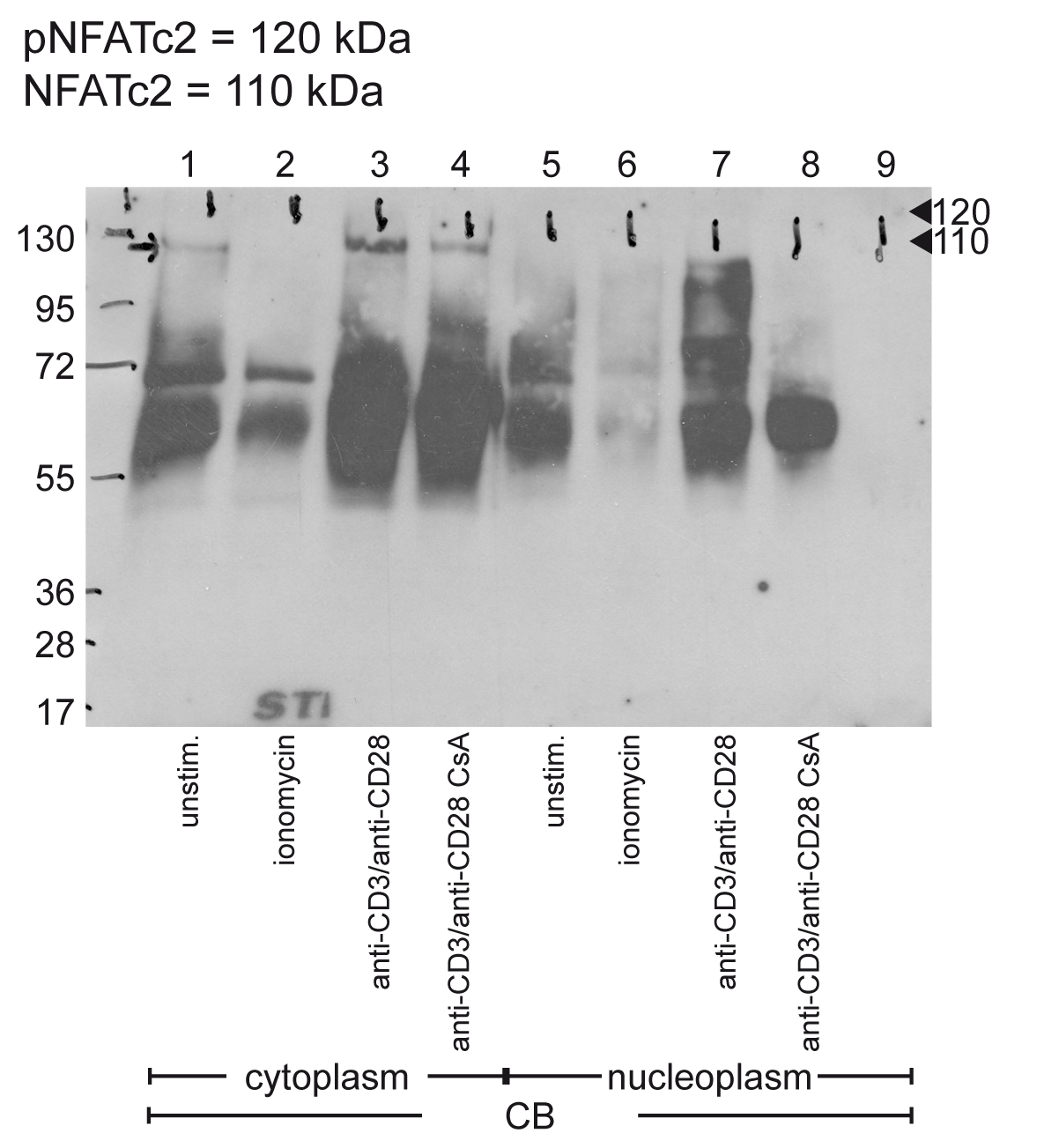

Supplement: S6 Fig — The NFATc2 protein expression in cytoplasm or nucleoplasm in naive CD4+ T cells of CB was detected and the phosphorylated (pNFATc2) and dephosphorylated (NFATc2) forms quantified. Cells were stimulated as indicated in the presence or absence of cyclosporin A (CsA). One representative experiment out of two comparable experiments is shown. unstim. = unstimulated. (TIF) [file pone.0166633.s006.tif]

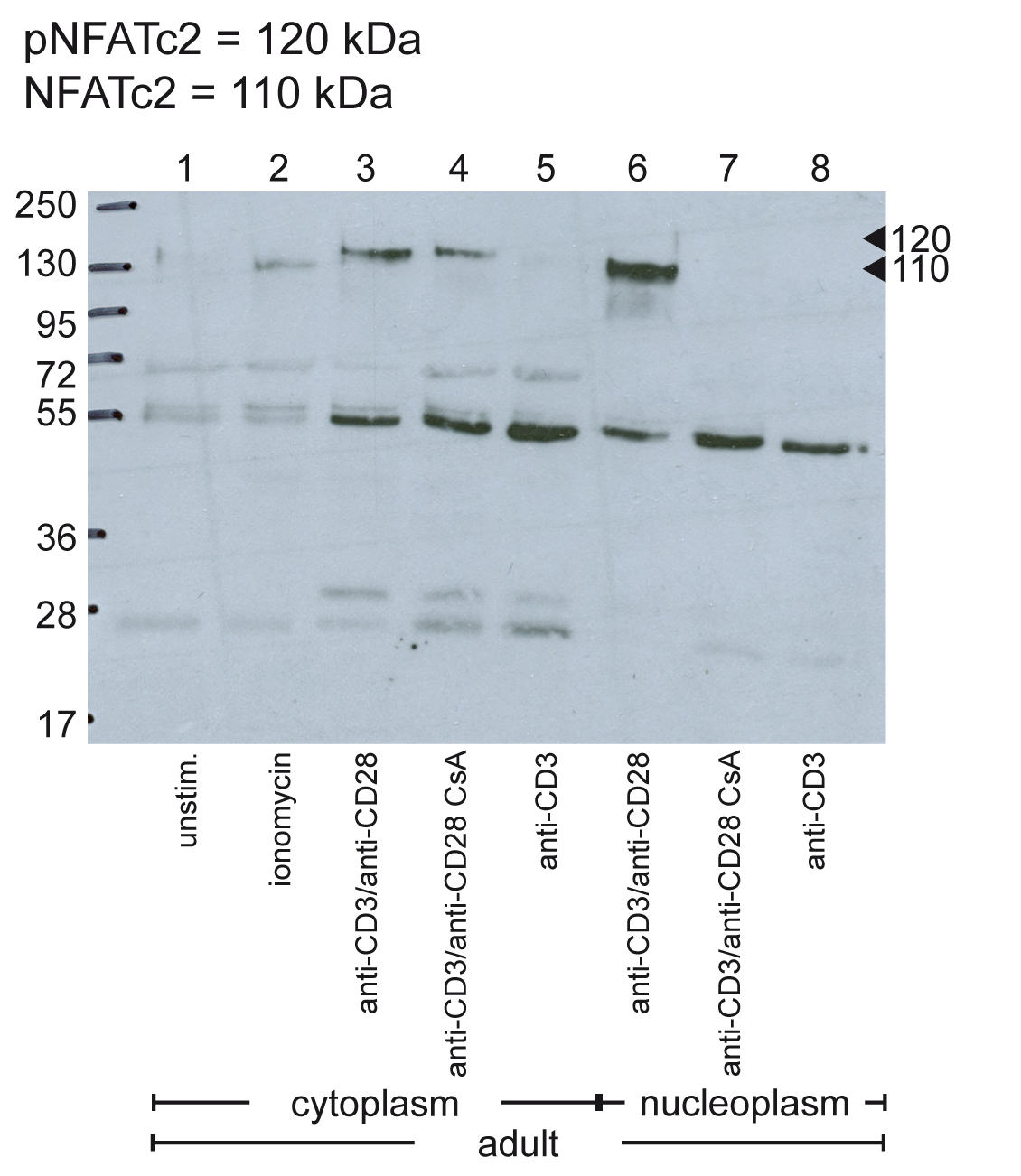

Supplement: S7 Fig — The NFATc2 protein expression in cytoplasm or nucleoplasm in CD31+ naive T cells of adult was detected and the phosphorylated (pNFATc2) and dephosphorylated (NFATc2) forms quantified. Cells were stimulated as indicated in the presence or absence of cyclosporin A (CsA). One representative experiment out of two comparable experiments is shown. unstim. = unstimulated. (TIF) [file pone.0166633.s007.tif]

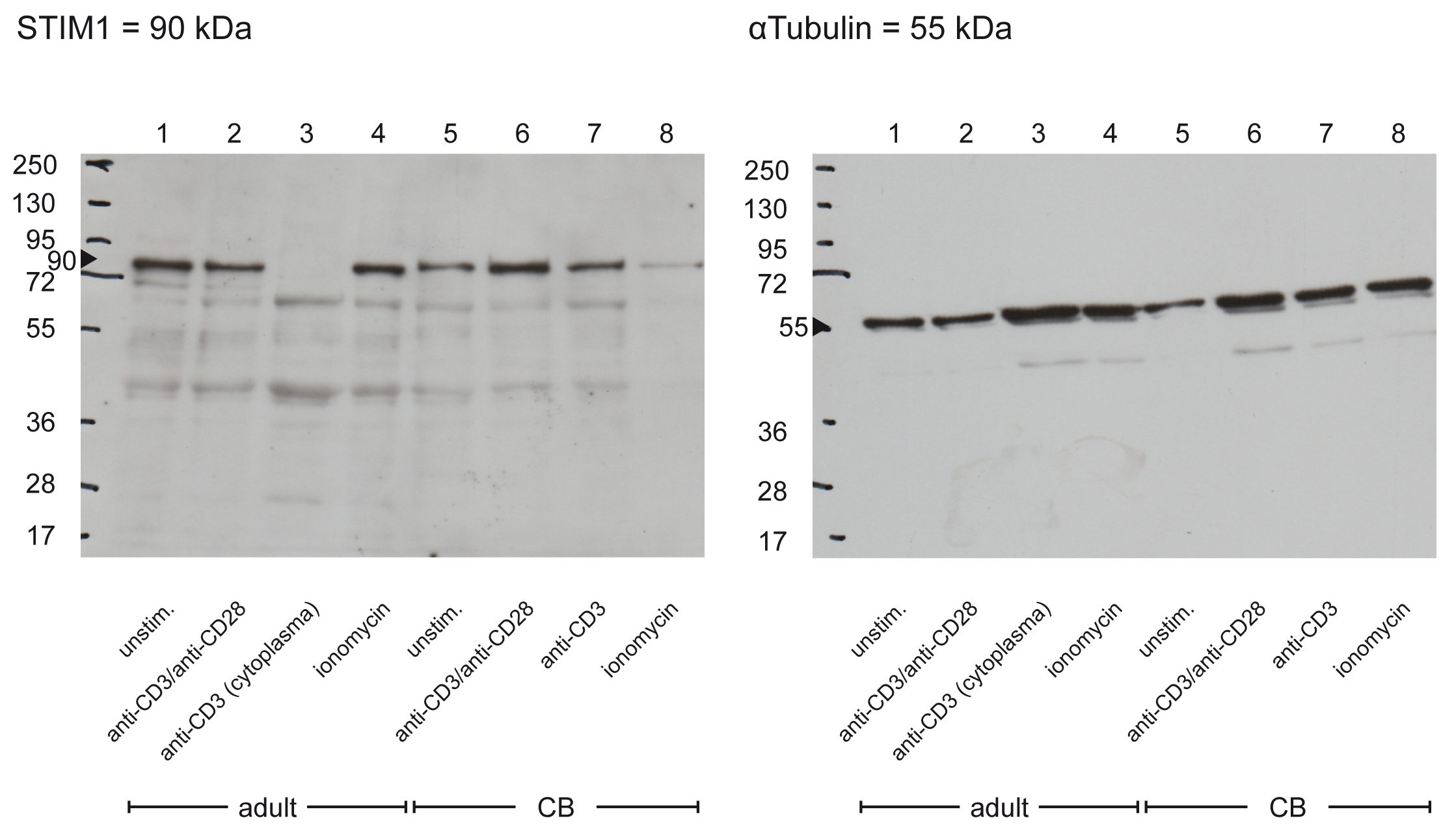

Supplement: S8 Fig — STIM1 protein expression in CD4+ T cells in naive CD31+ T cells from adults and CB after stimulation using anti-CD3/anti-CD28 Ab (to the right). Results are representative of at least two experiments. αTubulin was used as a loading control (to the left). (TIF) [file pone.0166633.s008.tif]

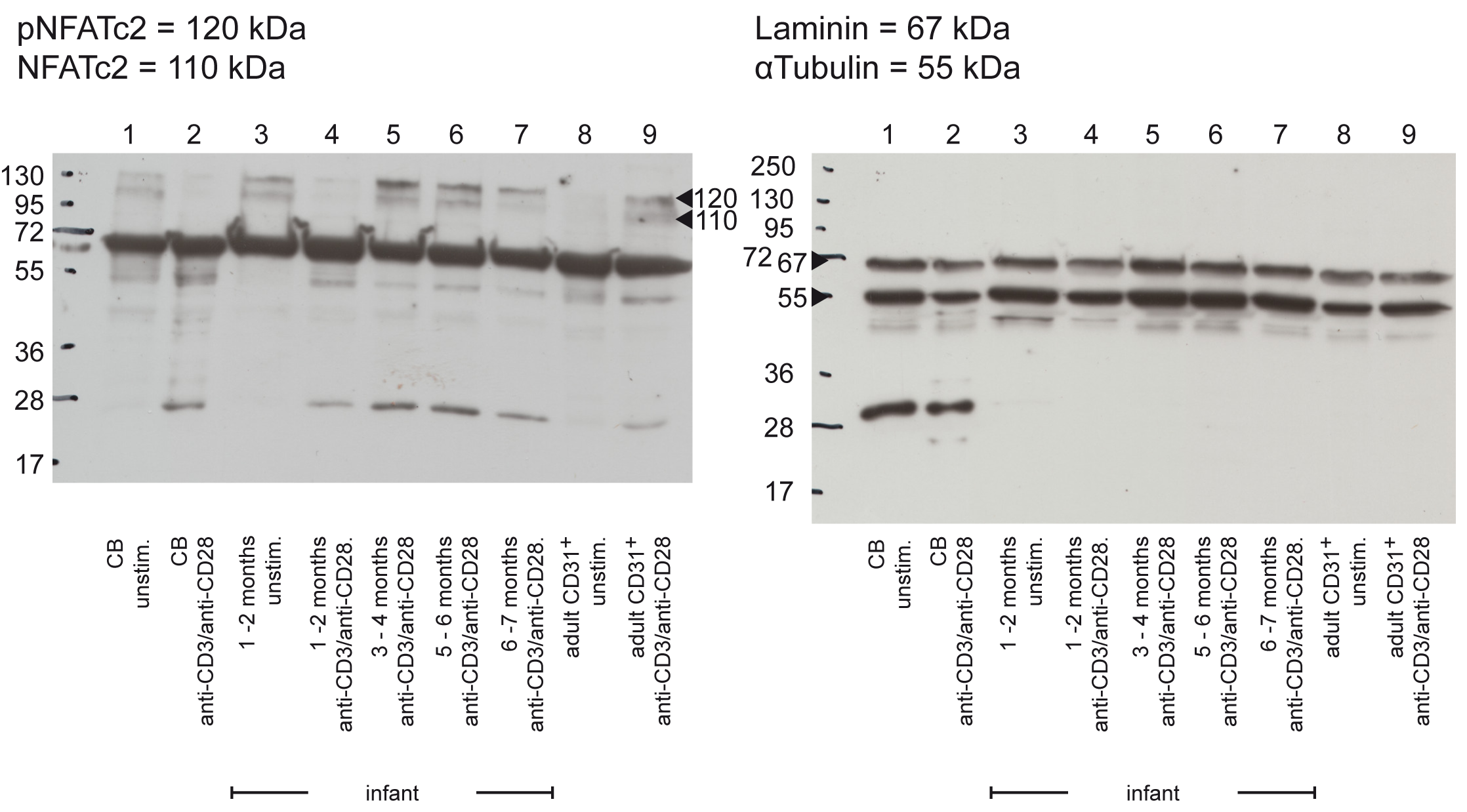

Supplement: S9 Fig — NFATc2 expression after anti-CD3 Ab plus anti-CD28 Ab engagement. The immunoblots are shown of NFATc2 and phosphorylated (pNFATc2) (to the right) and of αTubulin. Laminin and αTubulin was used as a loading control (to the left). Lysates from three different donors were pooled. Results are representative of at least two independent experiments. (TIF) [file pone.0166633.s009.tif]
