# Supplementary material for: T Cells of Infants Are Mature, but Hyporeactive Due to Limited Ca2+ Influx
Source: PLoS One. 2016 Nov 28;11(11):e0166633. doi: 10.1371/journal.pone.0166633 (PMC5125607; doi:10.1371/journal.pone.0166633)
Supplement: S1 Table — (DOCX) [file pone.0166633.s010.docx]

## S1 Table

**Relative** **frequencies of** **lymphocyte subpopulation in dependent on age.**

|  | **n** | **Subset CD4^+^ of lymphocytes (%) ± SD** | **CD45RA^+^ of CD4^+^ T cells (%) ± SD** | **CD31^+^ of CD4^+^CD45RA^+^ T cells (%) ± SD** |
| --- | --- | --- | --- | --- |
| CB | 33 | 53.9 ± 17.4 | 82.9 ± 17.5 | 79.5 ± 9.1 |
| Infant (1-2 mo) | 13 | 65.7 ± 13.6 | 87.0 ± 14.1 | 73.2 ± 10.0 |
| Infant (3-5 mo) | 18 | 58.0 ± 19.5 | 78.5 ± 20.0 | 77.2 ± 11.4 |
| Infant/child (6-66 mo) | 21 | 59.3 ± 17.4 | 69.7 ± 24.3 | 76.8 ± 12.2 |
| Adult | 28 | 57.6 ± 21.3 | 58.4 ± 18.7 | 74.8 ± 18.8 |

Lymphocytes subpopulations were analyzed by three colors flow cytometry using abs specific for CD4, CD45RA, and CD31. Selective gating based on the parameters of forward and side scatter was performed. Number (n) of sample. Table 1 presents a summary of the main parameters in form of mean ± standard deviation. mo = months.
