# Supplementary material for: T Cells of Infants Are Mature, but Hyporeactive Due to Limited Ca2+ Influx
Source: PLoS One. 2016 Nov 28;11(11):e0166633. doi: 10.1371/journal.pone.0166633 (PMC5125607; doi:10.1371/journal.pone.0166633)
Supplement: S2 Table — (DOCX) [file pone.0166633.s011.docx]

## S2 Table

**Summarized Analysis of variance (ANOVA) assessment for frequencies.**

|  | **5 groups of infants**  **(CB, infants 1-2 mo, infants 3-5 mo, infants 6-66 mo, adult)** |
| --- | --- |
| CD4^+^ | 0.4088 |
| CD4^+^CD45RA^+^ | <0.0001 |
| CD4^+^CD45RA^+^CD31^+^ | 0.5552 |

Frequencies of CD4^+^ among lymphocytes, of CD45RA^+^ among CD4^+^ T cells, and CD31^+^ among peripheral CD4^+^CD45^+^ cells are shown.
