# Supplementary material for: T Cells of Infants Are Mature, but Hyporeactive Due to Limited Ca2+ Influx
Source: PLoS One. 2016 Nov 28;11(11):e0166633. doi: 10.1371/journal.pone.0166633 (PMC5125607; doi:10.1371/journal.pone.0166633)
Supplement: S3 Table — (DOCX) [file pone.0166633.s012.docx]

## S3 Table

**Concrete single analysis of ANOVA for frequencies CD45RA^+^ among CD4^+^ T cells data for 5 groups of infants (CB, infants 1-2 mo, infants 3-5 mo, infants 6-66 mo, adult).**

difference

5 groups between Simultaneous 95%

comparison means confidence limits

------------------------------------------------------------------

Infant1_2 - neonatal 4.177 -13.344 21.698

Infant1_2 - Infant3_5 8.559 -10.916 28.034

Infant1_2 - Infant6_66 17.387 -1.496 36.270

Infant1_2 - Adult 28.666 10.708 46.623 ***

neonatal - Infant1_2 -4.177 -21.698 13.344

neonatal - Infant3_5 4.382 -11.297 20.060

neonatal - Infant6_66 13.210 -1.726 28.146

neonatal - Adult 24.489 10.741 38.237 ***

Infant3_5 - Infant1_2 -8.559 -28.034 10.916

Infant3_5 - neonatal -4.382 -20.060 11.297

Infant3_5 - Infant6_66 8.829 -8.358 26.015

Infant3_5 - Adult 20.107 3.943 36.272 ***

Infant6_66 - Infant1_2 -17.387 -36.270 1.496

Infant6_66 - neonatal -13.210 -28.146 1.726

Infant6_66 - Infant3_5 -8.829 -26.015 8.358

Infant6_66 - Adult 11.279 -4.167 26.725

Adult - Infant1_2 -28.666 -46.623 -10.708 ***

Adult - neonatal -24.489 -38.237 -10.741 ***

Adult - Infant3_5 -20.107 -36.272 -3.943 ***

Adult - Infant6_66 -11.279 -26.725 4.167

A mixed-model ANOVA was performed with individual as random effect followed by Tukey-Kramer post-hoc test for pairwise group comparisons (two-tailed). Comparisons are significant at the 0,05 level is indicated by ***.
