# Supplementary material for: T Cells of Infants Are Mature, but Hyporeactive Due to Limited Ca2+ Influx
Source: PLoS One. 2016 Nov 28;11(11):e0166633. doi: 10.1371/journal.pone.0166633 (PMC5125607; doi:10.1371/journal.pone.0166633)
Supplement: S4 Table — (DOCX) [file pone.0166633.s013.docx]

## S4 Table

**Normalized to ionomycin Ca^2+^ influx data with means and SD.**

| **T-cell subset** | **anti-CD3 Ab conc. (μg/ml)** | **anti-CD28 Ab** | **CB**  **(n)** | **Infant**  **1-2 mo**  **(n)** | **Infant**  **3-5 mo**  **(n)** | **Infant/-child**  **6-66 mo**  **(n)** | **Adult**  **(n)** |
| --- | --- | --- | --- | --- | --- | --- | --- |
| CD4^+^CD45RA^+^CD31^+^ | 0,005 | **-** | 0,15 ± 0,07 (6) | 0,08 ± 0,13 (4) | 0,07 ± 0,05 (6) | 0,08 ± 0,06 (6) | 0,17 ± 0,1 (9) |
| CD4^+^CD45RA^+^CD31^+^ | 0,005 | **+** | 0,23 ± 0,1 (9) | 0,11 ± 0,09 (5) | 0,07 ± 0,004 (4) | 0,07 ± 0,03 (4) | 0,31 ± 0,18 (9) |
| CD4^+^CD45RA^+^CD31^-^ | 0,005 | **-** | 0,18 ± 0,12 (5) | 0,09 ± 0,09 (3) | 0,11 ± 0,05 (6) | 0,07 ± 0,06 (6) | 0,22 ± 0,12 (7) |
| CD4^+^CD45RA^+^CD31^-^ | 0,005 | **+** | 0,2 ± 0,08 (9) | 0,1 ± 0,1 (5) | 0,14 ± 0,05 (3) | 0,09 ± 0,07 (4) | 0,35 ± 0,19 (9) |
| CD4^+^CD45RA^+^ | 0,005 | **-** | 0,11 ± 0,07 (4) | 0,12 ± 0,17 (4) | 0,09 ± 0,05 (6) | 0,06 ± 0,06 (6) | 0,21 ± 0,15 (7) |
| CD4^+^CD45RA^+^ | 0,005 | **+** | 0,18 ± 0,12 (7) | 0,08 ± 0,08 (4) | 0,14 ± 0,03 (4) | 0,07 ± 0,03 (4) | 0,36 ± 0,23 (8) |
| CD4^+^CD45RA^-^ | 0,005 | **-** | 0,14 ± 0,04 (3) | 0,04 ± 0,03 (4) | 0,07 ± 0,06 (6) | 0,10 ± 0,08 (6) | 0,12 ± 0,06 (7) |
| CD4^+^CD45RA^-^ | 0,005 | **+** | 0,17 ± 0,1 (6) | 0,09 ± 0,07 (5) | 0,16 ± 0,03 (4) | 0,09 ± 0,06 (4) | 0,22 ± 0,11 (8) |
| CD4^+^ | 0,005 | **-** | 0,18 ± 0,12 (6) | 0,1 ± 0,04 (4) | 0,11 ± 0,04 (6) | 0,06 ± 0,06 (6) | 0,1 ± 0,06 (7) |
| CD4^+^ | 0,005 | **+** | 0,18 ± 0,1 (9) | 0,09 ± 0,07 (5) | 0,14 ± 0,04 (4) | 0,03 ± 0,01 (4) | 0,25 ± 0,11 (9) |
| CD4^-^ | 0,005 | **-** | 0,09 ± 0,09 (6) | 0,03 ± 0,02 (4) | 0,12 ± 0,06 (6) | 0,07 ± 0,05 (6) | 0,03 ± 0,02 (7) |
| CD4^-^ | 0,005 | **+** | 0,07 ± 0,06 (9) | 0,04 ± 0,03 (5) | 0,13 ± 0,09 (4) | 0,04 ± 0,02 (4) | 0,06 ± 0,05 (9) |
| CD4^+^CD45RA^+^CD31^+^ | 0,05 | **-** | 0,66 ± 0,13 (10) | 0,39 ± 0,25 (5) | 0,15 ± 0,09 (6) | 0,37 ± 0,14 (4) | 0,41 ± 0,18 (10) |
| CD4^+^CD45RA^+^CD31^+^ | 0,05 | **+** | 0,67 ± 0,1 (7) | 0,39 ± 0,13 (7) | 0,08 ± 0,02 (6) | 0,3 ± 0,15 (4) | 0,52 ± 0,11 (8) |
| CD4^+^CD45RA^+^CD31^-^ | 0,05 | **-** | 0,59 ± 0,13 (9) | 0,29 ± 0,21 (5) | 0,19 ± 0,07 (6) | 0,25 ± 0,15 (4) | 0,4 ± 0,17 (8) |
| CD4^+^CD45RA^+^CD31^-^ | 0,05 | **+** | 0,54 ± 0,22 (7) | 0,26 ± 0,16 (7) | 0,18 ± 0,08 (5) | 0,28 ± 0,16 (4) | 0,49 ± 0,14 (8) |
| CD4^+^CD45RA^+^ | 0,05 | **-** | 0,67 ± 0,19 (8) | 0,43 ± 0,2 (5) | 0,18 ± 0,1 (6) | 0,28 ± 0,12 (4) | 0,44 ± 0,14 (8) |
| CD4^+^CD45RA^+^ | 0,05 | **+** | 0,58 ± 0,25 (7) | 0,35 ± 0,15 (7) | 0,13 ± 0,04 (6) | 0,28 ± 0,17 (4) | 0,56 ± 0,12 (7) |
| CD4^+^CD45RA^-^ | 0,05 | **-** | 0,61 ± 0,19 (6) | 0,23 ± 0,2 (5) | 0,21 ± 0,1 (6) | 0,34 ± 0,09 (3) | 0,26 ± 0,2 (8) |
| CD4^+^CD45RA^-^ | 0,05 | **+** | 0,52 ± 0,22 (6) | 0,29 ± 0,18 (7) | 0,19 ± 0,09 (6) | 0,32 ± 0,05 (4) | 0,37 ± 0,14 (6) |
| CD4^+^ | 0,05 | **-** | 0,61 ± 0,18 (10) | 0,39 ± 0,19 (5) | 0,27 ± 0,12 (4) | 0,27 ± 0,13 (4) | 0,33 ± 0,19 (8) |
| CD4^+^ | 0,05 | **+** | 0,59 ± 0,21 (7) | 0,33 ± 0,21 (7) | 0,26 ± 0,19 (4) | 0,26 ± 0,19 (4) | 0,46 ± 0,11 (8) |
| CD4^-^ | 0,05 | **-** | 0,1 ± 0,08 (10) | 0,06 ± 0,04 (5) | 0,16 ± 0,08 (6) | 0,11 ± 0,08 (4) | 0,09 ± 0,08 (8) |
| CD4^-^ | 0,05 | **+** | 0,13 ± 0,13 (7) | 0,05 ± 0,06 (7) | 0,12 ± 0,08 (6) | 0,05 ± 0,005 (4) | 0,04 ± 0,03 (8) |
| CD4^+^CD45RA^+^CD31^+^ | 0,5 | **-** | 0,81 ± 0,1 (11) | 0,64 ± 0,11 (6) | 0,45 ± 0,11 (6) | 0,63 ± 0,16 (8) | 0,66 ± 0,09 (9) |
| CD4^+^CD45RA^+^CD31^+^ | 0,5 | **+** | 0,74 ± 0,1 (10) | 0,68 ± 0,13 (7) | 0,36 ± 0,07 (6) | 0,67 ± 0,12 (7) | 0,65 ± 0,14 (10) |
| CD4^+^CD45RA^+^CD31^-^ | 0,5 | **-** | 0,74 ± 0,12 (10) | 0,51 ± 0,18 (5) | 0,44 ± 0,17 (6) | 0,55 ± 0,13 (8) | 0,62 ± 0,18 (8) |
| CD4^+^CD45RA^+^CD31^-^ | 0,5 | **+** | 0,64 ± 0,15 (10) | 0,54 ± 0,05 (7) | 0,37 ± 0,07 (6) | 0,55 ± 0,08 (7) | 0,6 ± 0,11 (9) |
| CD4^+^CD45RA^+^ | 0,5 | **-** | 0,82 ± 0,11 (10) | 0,59 ± 0,13 (6) | 0,45 ± 0,13 (6) | 0,61 ± 0,17 (8) | 0,69 ± 0,23 (8) |
| CD4^+^CD45RA^+^ | 0,5 | **+** | 0,72 ± 0,16 (8) | 0,57 ± 0,1 (7) | 0,43 ± 0,1 (6) | 0,64 ± 0,08 (7) | 0,71 ± 0,14 (8) |
| CD4^+^CD45RA^-^ | 0,5 | **-** | 0,72 ± 0,16 (7) | 0,4 ± 0,22 (6) | 0,39 ± 0,1 (6) | 0,47 ± 0,17 (7) | 0,55 ± 0,15 (8) |
| CD4^+^CD45RA^-^ | 0,5 | **+** | 0,57 ± 0,12 (7) | 0,45 ± 0,1 (7) | 0,34 ± 0,1 (6) | 0,51 ± 0,09 (7) | 0,51 ± 0,1 (7) |
| CD4^+^ | 0,5 | **-** | 0,76 ± 0,15 (11) | 0,62 ± 0,04 (6) | 0,48 ± 0,15 (6) | 0,59 ± 0,17 (8) | 0,6 ± 0,18 (8) |
| CD4^+^ | 0,5 | **+** | 0,67 ± 0,15 (10) | 0,52 ± 0,14 (7) | 0,42 ± 0,1 (6) | 0,62 ± 0,08 (7) | 0,6 ± 0,09 (8) |
| CD4^-^ | 0,5 | **-** | 0,14 ± 0,1 (11) | 0,16 ± 0,13 (6) | 0,17 ± 0,11 (6) | 0,1 ± 0,08 (8) | 0,14 ± 0,19 (8) |
| CD4^-^ | 0,5 | **+** | 0,09 ± 0,09 (10) | 0,06 ± 0,04 (7) | 0,13 ± 0,06 (6) | 0,07 ± 0,06 (7) | 0,09 ± 0,04 (8) |

Max. Ca^2+^ influx signal normalized to ionomycin (Normalization to ionomycin = (Ca^2+^ influx peak of GAMIG – Ca^2+^ influx of baseline) / (Ca^2+^ influx peak of ionomycin – Ca^2+^ influx of baseline)) of different individual sample (CB, infant, children and adult) for different T cell subset in dependence on the stimulation of anti-CD3 Ab for three different concentrations (0,005 μg/ml, 0,05 μg/ml and 0,5 μg/ml) with (+) or without (-) 0,5 μg/ml anti-CD28 Ab costimulation. conc. = concentration; number of samples marked in (n); mo = months.
