# Supplementary material for: T Cells of Infants Are Mature, but Hyporeactive Due to Limited Ca2+ Influx
Source: PLoS One. 2016 Nov 28;11(11):e0166633. doi: 10.1371/journal.pone.0166633 (PMC5125607; doi:10.1371/journal.pone.0166633)
Supplement: S5 Table — (DOCX) [file pone.0166633.s014.docx]

## S5 Table

**Summary of significant differences of two-tailed ANOVA of differences in the Ca^2+^ influx responses for the difference of subset of T cell for 3 different anti-CD3 Ab concentration (0.005 μg/ml, 0.05 μg/ml and 0.5 μg/ml) with or without anti-CD28 Ab stimulation of adult.**

| anti-CD3/anti-CD28 Ab | anti-CD3 Ab concentration 0.005 (μg/ml) | **subset of T cell** | CD31^+^ | CD31^-^ | CD45 RA^+^ | CD45 RA^-^ | CD4^+^ | CD4^-^ |
| --- | --- | --- | --- | --- | --- | --- | --- | --- |
|  |  | CD31^+^ | --- | 0,136 | 0,183 | 0,082 | 0,149 | 0,005 |
|  |  | CD31^-^ | 0,136 | --- | 0,822 | 0,013 | 0,022 | 0,003 |
|  |  | CD45RA^+^ | 0,183 | 0,822 | --- | 0,032 | 0,086 | 0,013 |
|  |  | CD45RA^-^ | 0,082 | 0,013 | 0,032 | --- | 0,004 | 0,007 |
|  |  | CD4^+^ | 0,149 | 0,022 | 0,086 | 0,004 | --- | 0,002 |
|  |  | CD4^-^ | 0,005 | 0,003 | 0,013 | 0,007 | 0,002 | --- |
| anti-CD3 Ab | anti-CD3 Ab concentration 0.005 (μg/ml) | **subset of T cell** | CD31^+^ | CD31^-^ | CD45RA^+^ | CD45RA^-^ | CD4^+^ | CD4^-^ |
|  |  | CD31^+^ | --- | 0,327 | 0,529 | 0,234 | 0,059 | 0,012 |
|  |  | CD31^-^ | 0,327 | --- | 0,871 | 0,150 | 0,046 | 0,012 |
|  |  | CD45RA^+^ | 0,529 | 0,871 | --- | 0,228 | 0,109 | 0,031 |
|  |  | CD45RA^-^ | 0,234 | 0,150 | 0,228 | --- | 0,518 | 0,026 |
|  |  | CD4^+^ | 0,059 | 0,046 | 0,109 | 0,518 | --- | 0,009 |
|  |  | CD4^-^ | 0,012 | 0,012 | 0,031 | 0,026 | 0,009 | --- |
| anti-CD3/anti-CD28 Ab | anti-CD3 Ab concentration 0.05 (μg/ml) | **subset of T cell** | CD31^+^ | CD31^-^ | CD45RA^+^ | CD45RA^-^ | CD4^+^ | CD4^-^ |
|  |  | CD31^+^ | --- | 0,006 | 0,823 | 0,142 | 0,192 | 0,001 |
|  |  | CD31^-^ | 0,006 | --- | 0,208 | 0,030 | 0,553 | <0,001 |
|  |  | CD45RA^+^ | 0,823 | 0,208 | --- | 0,006 | 0,057 | <0,001 |
|  |  | CD45RA^-^ | 0,142 | 0,030 | 0,006 | --- | 0,062 | <0,001 |
|  |  | CD4^+^ | 0,192 | 0,553 | 0,057 | 0,062 | --- | <0,001 |
|  |  | CD4^-^ | 0,001 | <0,001 | <0,001 | <0,001 | <0,001 | --- |
| anti-CD3 Ab | anti-CD3 Ab concentration 0.05 (μg/ml) | **subset of T cell** | CD31^+^ | CD31^-^ | CD45RA^+^ | CD45RA^-^ | CD4^+^ | CD4^-^ |
|  |  | CD31^+^ | --- | 0,224 | 0,107 | 0,697 | 0,511 | 0,006 |
|  |  | CD31^-^ | 0,224 | --- | 0,129 | 0,007 | 0,053 | 0,001 |
|  |  | CD45RA^+^ | 0,107 | 0,129 | --- | 0,002 | 0,024 | <0,001 |
|  |  | CD45RA^-^ | 0,697 | 0,007 | 0,002 | --- | 0,092 | 0,057 |
|  |  | CD4^+^ | 0,511 | 0,053 | 0,024 | 0,092 | --- | 0,005 |
|  |  | CD4^-^ | 0,006 | 0,001 | <0,001 | 0,057 | 0,005 | --- |
| anti-CD3/anti-CD28 Ab | anti-CD3 Ab concentration 0.5 (μg/ml) | **subset of T cell** | CD31^+^ | CD31^-^ | CD45RA^+^ | CD45RA^-^ | CD4^+^ | CD4^-^ |
|  |  | CD31^+^ | --- | 0,007 | 0,272 | 0,001 | 0,008 | <0,001 |
|  |  | CD31^-^ | 0,007 | --- | 0,019 | 0,003 | 0,965 | <0,001 |
|  |  | CD45RA^+^ | 0,272 | 0,019 | --- | <0,001 | 0,039 | <0,001 |
|  |  | CD45RA^-^ | 0,001 | 0,003 | <0,001 | --- | 0,047 | <0,001 |
|  |  | CD4^+^ | 0,008 | 0,965 | 0,039 | 0,047 | --- | <0,001 |
|  |  | CD4^-^ | <0,001 | <0,001 | <0,001 | <0,001 | <0,001 | --- |
| anti-CD3 Ab | anti-CD3 Ab concentration 0.5 (μg/ml) | **subset of T cell** | CD31^+^ | CD31^-^ | CD45RA^+^ | CD45RA^-^ | CD4^+^ | CD4^-^ |
|  |  | CD31^+^ | --- | 0,024 | 0,776 | <0,001 | 0,007 | <0,001 |
|  |  | CD31^-^ | 0,024 | --- | 0,096 | 0,019 | 0,063 | 0,001 |
|  |  | CD45RA^+^ | 0,776 | 0,096 | --- | 0,010 | 0,024 | 0,001 |
|  |  | CD45RA^-^ | <0,001 | 0,019 | 0,010 | --- | 0,057 | 0,002 |
|  |  | CD4^+^ | 0,007 | 0,063 | 0,024 | 0,057 | --- | 0,001 |
|  |  | CD4^-^ | <0,001 | 0,001 | 0,001 | 0,002 | 0,001 | --- |

The red marked numbers indicates pairwise significance (*p<*0.05). CD4^+^CD45RA^+^CD31^+^=CD31^+^; CD4^+^CD45RA^+^CD31^-^= CD31^-^; CD4^+^CD45RA^+^= CD45RA^+^; CD4^+^CD45RA^-^ = CD45RA^-^; CD4^+^ = CD4^+^; CD4^-^ = CD4^-^.
