# Supplementary material for: T Cells of Infants Are Mature, but Hyporeactive Due to Limited Ca2+ Influx
Source: PLoS One. 2016 Nov 28;11(11):e0166633. doi: 10.1371/journal.pone.0166633 (PMC5125607; doi:10.1371/journal.pone.0166633)
Supplement: S6 Table — (DOCX) [file pone.0166633.s015.docx]

## S6 Table

##

**Summary of two-tailed ANOVA of difference the Ca^2+^ influx responses for the difference of anti-CD3/CD28 Ab with anti-CD3 Ab.**

| anti-CD3 Ab concentration | T cell subset | **Difference between of anti-CD3/CD28 Ab and anti-CD3 Ab** | | | | |
| --- | --- | --- | --- | --- | --- | --- |
|  |  | CB | Infant1-2 mo | Infant 3-5  mo | Infant 6-66  mo | Adult |
| 0.005 μg/ml | CD31^+^ | 0,955 | 0,431 | 0,527 | 0,998 | 0,043 |
|  | CD31^-^ | 0,347 | 0,427 | 0,705 | 0,649 | 0,075 |
|  | CD45RA^+^ | 0,256 | 0,756 | 0,259 | 0,452 | 0,050 |
|  | CD45RA^-^ | 0,664 | 0,200 | 0,110 | 0,812 | 0,113 |
|  | CD4^+^ | 0,949 | 0,729 | 0,771 | 0,312 | 0,037 |
|  | CD4^-^ | 0,280 | 0,782 | 0,589 | 0,303 | 0,751 |
| 0.05 μg/ml | CD31^+^ | 0,775 | 0,654 | 0,053 | 0,546 | 0,036 |
|  | CD31^-^ | 0,955 | 0,831 | 0,401 | 0,894 | 0,334 |
|  | CD45RA^+^ | 0,820 | 0,653 | 0,358 | 0,921 | 0,048 |
|  | CD45RA^-^ | 0,650 | 0,475 | 0,636 | 0,859 | 0,072 |
|  | CD4^+^ | 0,993 | 0,827 | 0,924 | 0,840 | 0,157 |
|  | CD4^-^ | 0,888 | 0,571 | 0,250 | 0,533 | 0,141 |
| 0.5 μg/ml | CD31^+^ | 0,314 | 0,454 | 0,086 | 0,533 | 0,795 |
|  | CD31^-^ | 0,310 | 0,643 | 0,433 | 0,776 | 0,863 |
|  | CD45RA^+^ | 0,635 | 0,959 | 0,694 | 0,814 | 0,353 |
|  | CD45RA^-^ | 0,486 | 0,731 | 0,431 | 0,549 | 0,817 |
|  | CD4^+^ | 0,408 | 0,257 | 0,146 | 0,710 | 0,601 |
|  | CD4^-^ | 0,205 | 0,113 | 0,181 | 0,070 | 0,495 |

Normalized Ca^2+^ influx responses for the difference of anti-CD3/CD28 Ab with anti-CD3 Ab for the 6 different subsets of T cell for 5 groups of individuals (CB, infants 1-2 months, infants 3-5 months, infants 6-66 months, adult) for three different anti-CD3 Ab concentrations (0,005 μg/ml, 0,05 μg/ml and 0,5 μg/ml). The red marked numbers indicates pairwise significance (*p*<0.05). CD4^+^CD45RA^+^CD31^+^=CD31^+^; CD4^+^CD45RA^+^CD31^-^= CD31^-^; CD4^+^CD45RA^+^= CD45RA^+^; CD4^+^CD45RA^-^ = CD45RA^-^; CD4^+^ = CD4^+^; CD4^-^ = CD4^-^; mo = months.
