# Supplementary material for: T Cells of Infants Are Mature, but Hyporeactive Due to Limited Ca2+ Influx
Source: PLoS One. 2016 Nov 28;11(11):e0166633. doi: 10.1371/journal.pone.0166633 (PMC5125607; doi:10.1371/journal.pone.0166633)
Supplement: S7 Table — (DOCX) [file pone.0166633.s016.docx]

## S7 Table

**Summary of Analysis of variance (ANOVA) assessment for Ca^2+^ influx data for the different subset of T cell type for 3 different anti-CD3 Ab concentration (0.005 μg/ml, 0.05 μg/ml and 0.5 μg/ml) with (+) or without (-) anti-CD28 Ab stimulation of adult.**

|  | **anti-CD28 Ab** | **anti-CD3 Ab concentration**  **(μg/ml)** | | |
| --- | --- | --- | --- | --- |
|  |  | 0.005 | 0.05 | 0.5 |
| CD4^+^CD45RA^+^CD31^+^ | + | 0.0023 | <0.0001 | <0.0001 |
| CD4^+^CD45RA^+^CD31^+^ | - | 0.0875 | <0.0001 | 0.0001 |
| CD4^+^CD45RA^+^CD31^-^ | + | 0.0051 | 0.0018 | 0.0006 |
| CD4^+^CD45RA^+^CD31^-^ | - | 0.0628 | 0.0002 | 0.0057 |
| CD4^+^CD45RA^+^ | + | 0.0105 | 0.0001 | 0.0005 |
| CD4^+^CD45RA^+^ | - | 0.1948 | 0.0001 | 0.0015 |
| CD4^+^CD45RA^-^ | + | 0.1281 | 0.0124 | 0.0055 |
| CD4^+^CD45RA^-^ | - | 0.2248 | 0.0033 | 0.0038 |
| CD4^+^ | + | 0.0023 | 0.0016 | 0.0032 |
| CD4^+^ | - | 0.0810 | 0.0002 | 0.0113 |
| CD4^-^ | + | 0.1607 | 0.1254 | 0.4153 |
| CD4^-^ | - | 0.0313 | 0.1632 | 0.8868 |

The red marked numbers indicates pairwise significance (*p*<0.05).
