# Supplementary material for: T Cells of Infants Are Mature, but Hyporeactive Due to Limited Ca2+ Influx
Source: PLoS One. 2016 Nov 28;11(11):e0166633. doi: 10.1371/journal.pone.0166633 (PMC5125607; doi:10.1371/journal.pone.0166633)
Supplement: S9 Table — (DOCX) [file pone.0166633.s018.docx]

## S9 Table

**Concrete single analysis of Analysis of variance (ANOVA) assessment for Ca^2+^ influx data for the different subset of T cell type for 5 groups of individuals (CB, infants 1-2 months, infants 3-5 months, infants 6-66 months, adult) and for 3 different Anti-CD3 Ab concentration (0.005 μg/ml, 0.05 μg/ml and 0.5 μg/ml).** Comparisons are significant at the 0,05 level are indicated by ***.

**Anti-CD3 Ab concentration =0.005 μg/ml – ANOVA**

Tukey test of the studentized range (HSD) for **anti-CD3/CD28 Ab** CD4^+^CD45RA^+^**CD31^+^**

difference

5 groups between Simultaneous 95%

comparison means confidence limits

-------------------------------------------------------------------------

adult - neonata 0.08092 -0.08120 0.24303

adult - Kind <=2Monate 0.20238 0.01057 0.39420 ***

adult - Kind>=5Monate 0.24509 0.03843 0.45175 ***

adult - Kind2-<5Monate 0.24948 0.04282 0.45614 ***

neonata - adult -0.08092 -0.24303 0.08120

neonata - Kind <=2Monate 0.12147 -0.07035 0.31329

neonata - Kind>=5Monate 0.16418 -0.04248 0.37084

neonata - Kind2-<5Monate 0.16856 -0.03810 0.37522

Kind <=2Monate - adult -0.20238 -0.39420 -0.01057 ***

Kind <=2Monate - neonata -0.12147 -0.31329 0.07035

Kind <=2Monate - Kind>=5Monate 0.04271 -0.18799 0.27341

Kind <=2Monate - Kind2-<5Monate 0.04709 -0.18361 0.27779

Kind>=5Monate - adult -0.24509 -0.45175 -0.03843 ***

Kind>=5Monate - neonata -0.16418 -0.37084 0.04248

Kind>=5Monate - Kind <=2Monate -0.04271 -0.27341 0.18799

Kind>=5Monate - Kind2-<5Monate 0.00438 -0.23879 0.24756

Kind2-<5Monate - adult -0.24948 -0.45614 -0.04282 ***

Kind2-<5Monate - neonata -0.16856 -0.37522 0.03810

Kind2-<5Monate - Kind <=2Monate -0.04709 -0.27779 0.18361

Kind2-<5Monate - Kind>=5Monate -0.00438 -0.24756 0.23879

Tukey test of the studentized range (HSD) for **anti-CD3/CD28 Ab** CD4^+^CD45RA^+^**CD31^-^**

difference

5 groups between Simultaneous 95%

comparison means confidence limits

------------------------------------------------------------------------

adult - neonata 0.14676 -0.02619 0.31972

adult - Kind2-<5Monate 0.20854 -0.03606 0.45313

adult - Kind <=2Monate 0.25014 0.04550 0.45479 ***

adult - Kind>=5Monate 0.25096 0.03048 0.47143 ***

neonata - adult -0.14676 -0.31972 0.02619

neonata - Kind2-<5Monate 0.06177 -0.18282 0.30637

neonata - Kind <=2Monate 0.10338 -0.10126 0.30802

neonata - Kind>=5Monate 0.10419 -0.11628 0.32467

Kind2-<5Monate - adult -0.20854 -0.45313 0.03606

Kind2-<5Monate - neonata -0.06177 -0.30637 0.18282

Kind2-<5Monate - Kind <=2Monate 0.04161 -0.22633 0.30955

Kind2-<5Monate - Kind>=5Monate 0.04242 -0.23780 0.32264

Kind <=2Monate - adult -0.25014 -0.45479 -0.04550 ***

Kind <=2Monate - neonata -0.10338 -0.30802 0.10126

Kind <=2Monate - Kind2-<5Monate -0.04161 -0.30955 0.22633

Kind <=2Monate - Kind>=5Monate 0.00081 -0.24531 0.24693

Kind>=5Monate - adult -0.25096 -0.47143 -0.03048 ***

Kind>=5Monate - neonata -0.10419 -0.32467 0.11628

Kind>=5Monate - Kind2-<5Monate -0.04242 -0.32264 0.23780

Kind>=5Monate - Kind <=2Monate -0.00081 -0.24693 0.24531

Tukey test of the studentized range (HSD) for **anti-CD3/CD28 Ab** CD4^+^**CD45RA^+^**

difference

5 groups between Simultaneous 95%

comparison means confidence limits

------------------------------------------------------------------------

adult - neonata 0.18502 -0.04040 0.41045

adult - Kind2-<5Monate 0.22398 -0.04275 0.49072

adult - Kind <=2Monate 0.28213 0.03381 0.53044 ***

adult - Kind>=5Monate 0.29144 0.02470 0.55817 ***

neonata - adult -0.18502 -0.41045 0.04040

neonata - Kind2-<5Monate 0.03896 -0.23405 0.31197

neonata - Kind <=2Monate 0.09710 -0.15794 0.35214

neonata - Kind>=5Monate 0.10641 -0.16660 0.37942

Kind2-<5Monate - adult -0.22398 -0.49072 0.04275

Kind2-<5Monate - neonata -0.03896 -0.31197 0.23405

Kind2-<5Monate - Kind <=2Monate 0.05814 -0.23405 0.35033

Kind2-<5Monate - Kind>=5Monate 0.06745 -0.24054 0.37545

Kind <=2Monate - adult -0.28213 -0.53044 -0.03381 ***

Kind <=2Monate - neonata -0.09710 -0.35214 0.15794

Kind <=2Monate - Kind2-<5Monate -0.05814 -0.35033 0.23405

Kind <=2Monate - Kind>=5Monate 0.00931 -0.28288 0.30150

Kind>=5Monate - adult -0.29144 -0.55817 -0.02470 ***

Kind>=5Monate - neonata -0.10641 -0.37942 0.16660

Kind>=5Monate - Kind2-<5Monate -0.06745 -0.37545 0.24054

Kind>=5Monate - Kind <=2Monate -0.00931 -0.30150 0.28288

Tukey test of the studentized range (HSD) for **anti-CD3/CD28 Ab** **CD4^+^**

difference

5 groups between Simultaneous 95%

comparison means confidence limits

-------------------------------------------------------------------------

adult - neonata 0.07106 -0.04933 0.19145

adult - Kind2-<5Monate 0.11071 -0.04276 0.26418

adult - Kind <=2Monate 0.16095 0.01851 0.30340 ***

adult - Kind>=5Monate 0.21832 0.06486 0.37179 ***

neonata - adult -0.07106 -0.19145 0.04933

neonata - Kind2-<5Monate 0.03965 -0.11381 0.19312

neonata - Kind <=2Monate 0.08990 -0.05255 0.23234

neonata - Kind>=5Monate 0.14727 -0.00620 0.30073

Kind2-<5Monate - adult -0.11071 -0.26418 0.04276

Kind2-<5Monate - neonata -0.03965 -0.19312 0.11381

Kind2-<5Monate - Kind <=2Monate 0.05024 -0.12108 0.22156

Kind2-<5Monate - Kind>=5Monate 0.10761 -0.07297 0.28820

Kind <=2Monate - adult -0.16095 -0.30340 -0.01851 ***

Kind <=2Monate - neonata -0.08990 -0.23234 0.05255

Kind <=2Monate - Kind2-<5Monate -0.05024 -0.22156 0.12108

Kind <=2Monate - Kind>=5Monate 0.05737 -0.11395 0.22869

Kind>=5Monate - adult -0.21832 -0.37179 -0.06486 ***

Kind>=5Monate - neonata -0.14727 -0.30073 0.00620

Kind>=5Monate - Kind2-<5Monate -0.10761 -0.28820 0.07297

Kind>=5Monate - Kind <=2Monate -0.05737 -0.22869 0.11395

Tukey test of the studentized range (HSD) for **anti-CD3 Ab** **CD4^+^**

difference

5 groups between Simultaneous 95%

comparison means confidence limits

------------------------------------------------------------------------

neonata - Kind2-<5Monate 0.07506 -0.04244 0.19255

neonata - adult 0.07802 -0.03520 0.19124

neonata - Kind <=2Monate 0.08505 -0.04631 0.21642

neonata - Kind>=5Monate 0.11889 0.00139 0.23638 ***

Kind2-<5Monate - neonata -0.07506 -0.19255 0.04244

Kind2-<5Monate - adult 0.00297 -0.11026 0.11619

Kind2-<5Monate - Kind <=2Monate 0.00999 -0.12137 0.14136

Kind2-<5Monate - Kind>=5Monate 0.04383 -0.07367 0.16133

adult - neonata -0.07802 -0.19124 0.03520

adult - Kind2-<5Monate -0.00297 -0.11619 0.11026

adult - Kind <=2Monate 0.00703 -0.12053 0.13459

adult - Kind>=5Monate 0.04087 -0.07236 0.15409

Kind <=2Monate - neonata -0.08505 -0.21642 0.04631

Kind <=2Monate - Kind2-<5Monate -0.00999 -0.14136 0.12137

Kind <=2Monate - adult -0.00703 -0.13459 0.12053

Kind <=2Monate - Kind>=5Monate 0.03384 -0.09753 0.16520

Kind>=5Monate - neonata -0.11889 -0.23638 -0.00139 ***

Kind>=5Monate - Kind2-<5Monate -0.04383 -0.16133 0.07367

Kind>=5Monate - adult -0.04087 -0.15409 0.07236

Kind>=5Monate - Kind <=2Monate -0.03384 -0.16520 0.09753

Tukey test of the studentized range (HSD) for **anti-CD3 Ab** **CD4^-^**

difference

5 groups between Simultaneous 95%

comparison means confidence limits

-----------------------------------------------------------------------

Kind2-<5Monate - neonata 0.03394 -0.05949 0.12738

Kind2-<5Monate - Kind>=5Monate 0.05484 -0.03859 0.14827

Kind2-<5Monate - adult 0.09482 0.00478 0.18485 ***

Kind2-<5Monate - Kind <=2Monate 0.09530 -0.00916 0.19976

neonata - Kind2-<5Monate -0.03394 -0.12738 0.05949

neonata - Kind>=5Monate 0.02089 -0.07254 0.11433

neonata - adult 0.06087 -0.02916 0.15091

neonata - Kind <=2Monate 0.06135 -0.04311 0.16582

Kind>=5Monate - Kind2-<5Monate -0.05484 -0.14827 0.03859

Kind>=5Monate - neonata -0.02089 -0.11433 0.07254

Kind>=5Monate - adult 0.03998 -0.05005 0.13001

Kind>=5Monate - Kind <=2Monate 0.04046 -0.06400 0.14492

adult - Kind2-<5Monate -0.09482 -0.18485 -0.00478 ***

adult - neonata -0.06087 -0.15091 0.02916

adult - Kind>=5Monate -0.03998 -0.13001 0.05005

adult - Kind <=2Monate 0.00048 -0.10095 0.10191

Kind <=2Monate - Kind2-<5Monate -0.09530 -0.19976 0.00916

Kind <=2Monate - neonata -0.06135 -0.16582 0.04311

Kind <=2Monate - Kind>=5Monate -0.04046 -0.14492 0.06400

Kind <=2Monate - adult -0.00048 -0.10191 0.10095

**Anti-CD3 Ab concentration =0.05 μg/ml – ANOVA**

Tukey test of the studentized range (HSD) for **anti-CD3/CD28 Ab** CD4^+^CD45RA^+^**CD31^+^**

difference

5 groups between Simultaneous 95%

comparison means confidence limits

----------------------------------------------------------------------------

neonata - adult 0.14916 -0.01465 0.31298

neonata - Kind <=2Monate 0.27882 0.10963 0.44801 ***

neonata - Kind>=5Monate 0.37809 0.17970 0.57649 ***

neonata - Kind2-<5Monate 0.59282 0.41673 0.76892 ***

adult - neonata -0.14916 -0.31298 0.01465

adult - Kind <=2Monate 0.12966 -0.03416 0.29347

adult - Kind>=5Monate 0.22893 0.03510 0.42276 ***

adult - Kind2-<5Monate 0.44366 0.27272 0.61460 ***

Kind <=2Monate - neonata -0.27882 -0.44801 -0.10963 ***

Kind <=2Monate - adult -0.12966 -0.29347 0.03416

Kind <=2Monate - Kind>=5Monate 0.09927 -0.09912 0.29767

Kind <=2Monate - Kind2-<5Monate 0.31400 0.13791 0.49010 ***

Kind>=5Monate - neonata -0.37809 -0.57649 -0.17970 ***

Kind>=5Monate - adult -0.22893 -0.42276 -0.03510 ***

Kind>=5Monate - Kind <=2Monate -0.09927 -0.29767 0.09912

Kind>=5Monate - Kind2-<5Monate 0.21473 0.01042 0.41905 ***

Kind2-<5Monate - neonata -0.59282 -0.76892 -0.41673 ***

Kind2-<5Monate - adult -0.44366 -0.61460 -0.27272 ***

Kind2-<5Monate - Kind <=2Monate -0.31400 -0.49010 -0.13791 ***

Kind2-<5Monate - Kind>=5Monate -0.21473 -0.41905 -0.01042 ***

Tukey test of the studentized range (HSD) for **anti-CD3 Ab** CD4^+^CD45RA^+^**CD31^+^**

difference

5 groups between Simultaneous 95%

comparison means confidence limits

-------------------------------------------------------------------------------

neonata - adult 0.25250 0.03885 0.46614 ***

neonata - Kind <=2Monate 0.26915 0.00749 0.53080 ***

neonata - Kind>=5Monate 0.29071 0.00809 0.57334 ***

neonata - Kind2-<5Monate 0.51484 0.26815 0.76153 ***

adult - neonata -0.25250 -0.46614 -0.03885 ***

adult - Kind <=2Monate 0.01665 -0.24501 0.27831

adult - Kind>=5Monate 0.03822 -0.24440 0.32084

adult - Kind2-<5Monate 0.26234 0.01565 0.50904 ***

Kind <=2Monate - neonata -0.26915 -0.53080 -0.00749 ***

Kind <=2Monate - adult -0.01665 -0.27831 0.24501

Kind <=2Monate - Kind>=5Monate 0.02157 -0.29890 0.34203

Kind <=2Monate - Kind2-<5Monate 0.24569 -0.04358 0.53497

Kind>=5Monate - neonata -0.29071 -0.57334 -0.00809 ***

Kind>=5Monate - adult -0.03822 -0.32084 0.24440

Kind>=5Monate - Kind <=2Monate -0.02157 -0.34203 0.29890

Kind>=5Monate - Kind2-<5Monate 0.22413 -0.08424 0.53249

Kind2-<5Monate - neonata -0.51484 -0.76153 -0.26815 ***

Kind2-<5Monate - adult -0.26234 -0.50904 -0.01565 ***

Kind2-<5Monate - Kind <=2Monate -0.24569 -0.53497 0.04358

Kind2-<5Monate - Kind>=5Monate -0.22413 -0.53249 0.08424

Tukey test of the studentized range (HSD) for **anti-CD3/CD28 Ab** CD4^+^CD45RA^+^**CD31^-^**

difference

5 groups between Simultaneous 95%

comparison means confidence limits

-------------------------------------------------------------------------

neonata - adult 0.05139 -0.19640 0.29919

neonata - Kind>=5Monate 0.25673 -0.04336 0.55682

neonata - Kind <=2Monate 0.27730 0.02138 0.53321 ***

neonata - Kind2-<5Monate 0.36615 0.08581 0.64649 ***

adult - neonata -0.05139 -0.29919 0.19640

adult - Kind>=5Monate 0.20534 -0.08785 0.49853

adult - Kind <=2Monate 0.22590 -0.02189 0.47369

adult - Kind2-<5Monate 0.31476 0.04181 0.58770 ***

Kind>=5Monate - neonata -0.25673 -0.55682 0.04336

Kind>=5Monate - adult -0.20534 -0.49853 0.08785

Kind>=5Monate - Kind <=2Monate 0.02056 -0.27953 0.32065

Kind>=5Monate - Kind2-<5Monate 0.10942 -0.21176 0.43059

Kind <=2Monate - neonata -0.27730 -0.53321 -0.02138 ***

Kind <=2Monate - adult -0.22590 -0.47369 0.02189

Kind <=2Monate - Kind>=5Monate -0.02056 -0.32065 0.27953

Kind <=2Monate - Kind2-<5Monate 0.08886 -0.19149 0.36920

Kind2-<5Monate - neonata -0.36615 -0.64649 -0.08581 ***

Kind2-<5Monate - adult -0.31476 -0.58770 -0.04181 ***

Kind2-<5Monate - Kind>=5Monate -0.10942 -0.43059 0.21176

Kind2-<5Monate - Kind <=2Monate -0.08886 -0.36920 0.19149

Tukey test of the studentized range (HSD) for **anti-CD3 Ab** CD4^+^CD45RA^+^**CD31^-^**

difference

5 groups between Simultaneous 95%

comparison means confidence limits

---------------------------------------------------------------------------

neonata - adult 0.18513 -0.02762 0.39788

neonata - Kind <=2Monate 0.30386 0.05965 0.54807 ***

neonata - Kind>=5Monate 0.33460 0.07150 0.59770 ***

neonata - Kind2-<5Monate 0.40339 0.17264 0.63415 ***

adult - neonata -0.18513 -0.39788 0.02762

adult - Kind <=2Monate 0.11873 -0.13087 0.36833

adult - Kind>=5Monate 0.14947 -0.11864 0.41759

adult - Kind2-<5Monate 0.21826 -0.01819 0.45472

Kind <=2Monate - neonata -0.30386 -0.54807 -0.05965 ***

Kind <=2Monate - adult -0.11873 -0.36833 0.13087

Kind <=2Monate - Kind>=5Monate 0.03074 -0.26296 0.32444

Kind <=2Monate - Kind2-<5Monate 0.09953 -0.16559 0.36465

Kind>=5Monate - neonata -0.33460 -0.59770 -0.07150 ***

Kind>=5Monate - adult -0.14947 -0.41759 0.11864

Kind>=5Monate - Kind <=2Monate -0.03074 -0.32444 0.26296

Kind>=5Monate - Kind2-<5Monate 0.06879 -0.21383 0.35141

Kind2-<5Monate - neonata -0.40339 -0.63415 -0.17264 ***

Kind2-<5Monate - adult -0.21826 -0.45472 0.01819

Kind2-<5Monate - Kind <=2Monate -0.09953 -0.36465 0.16559

Kind2-<5Monate - Kind>=5Monate -0.06879 -0.35141 0.21383

Tukey test of the studentized range (HSD) for **anti-CD3/CD28 Ab** CD4^+^**CD45RA^+^**

difference

5 groups between Simultaneous 95%

comparison means confidence limits

----------------------------------------------------------------------

neonata - adult 0.02649 -0.22704 0.28002

neonata - Kind <=2Monate 0.23927 -0.01427 0.49280

neonata - Kind>=5Monate 0.30204 0.00475 0.59934 ***

neonata - Kind2-<5Monate 0.45243 0.18855 0.71632 ***

adult - neonata -0.02649 -0.28002 0.22704

adult - Kind <=2Monate 0.21278 -0.04076 0.46631

adult - Kind>=5Monate 0.27555 -0.02174 0.57285

adult - Kind2-<5Monate 0.42594 0.16206 0.68983 ***

Kind <=2Monate - neonata -0.23927 -0.49280 0.01427

Kind <=2Monate - adult -0.21278 -0.46631 0.04076

Kind <=2Monate - Kind>=5Monate 0.06277 -0.23452 0.36007

Kind <=2Monate - Kind2-<5Monate 0.21317 -0.05072 0.47705

Kind>=5Monate - neonata -0.30204 -0.59934 -0.00475 ***

Kind>=5Monate - adult -0.27555 -0.57285 0.02174

Kind>=5Monate - Kind <=2Monate -0.06277 -0.36007 0.23452

Kind>=5Monate - Kind2-<5Monate 0.15039 -0.15578 0.45656

Kind2-<5Monate - neonata -0.45243 -0.71632 -0.18855 ***

Kind2-<5Monate - adult -0.42594 -0.68983 -0.16206 ***

Kind2-<5Monate - Kind <=2Monate -0.21317 -0.47705 0.05072

Kind2-<5Monate - Kind>=5Monate -0.15039 -0.45656 0.15578

Tukey test of the studentized range (HSD) for **anti-CD3 Ab** CD4^+^**CD45RA^+^**

difference

5 groups between Simultaneous 95%

comparison means confidence limits

-----------------------------------------------------------------------

neonata - adult 0.22297 -0.01139 0.45732

neonata - Kind <=2Monate 0.23828 -0.02893 0.50548

neonata - Kind>=5Monate 0.38475 0.09773 0.67177 ***

neonata - Kind2-<5Monate 0.49032 0.23719 0.74345 ***

adult - neonata -0.22297 -0.45732 0.01139

adult - Kind <=2Monate 0.01531 -0.25189 0.28251

adult - Kind>=5Monate 0.16179 -0.12523 0.44881

adult - Kind2-<5Monate 0.26736 0.01423 0.52049 ***

Kind <=2Monate - neonata -0.23828 -0.50548 0.02893

Kind <=2Monate - adult -0.01531 -0.28251 0.25189

Kind <=2Monate - Kind>=5Monate 0.14648 -0.16794 0.46089

Kind <=2Monate - Kind2-<5Monate 0.25205 -0.03177 0.53586

Kind>=5Monate - neonata -0.38475 -0.67177 -0.09773 ***

Kind>=5Monate - adult -0.16179 -0.44881 0.12523

Kind>=5Monate - Kind <=2Monate -0.14648 -0.46089 0.16794

Kind>=5Monate - Kind2-<5Monate 0.10557 -0.19698 0.40812

Kind2-<5Monate - neonata -0.49032 -0.74345 -0.23719 ***

Kind2-<5Monate - adult -0.26736 -0.52049 -0.01423 ***

Kind2-<5Monate - Kind <=2Monate -0.25205 -0.53586 0.03177

Kind2-<5Monate - Kind>=5Monate -0.10557 -0.40812 0.19698

Tukey test of the studentized range (HSD) for **anti-CD3/CD28 Ab** CD4^+^**CD45RA^-^**

difference

5 groups between Simultaneous 95%

comparison means confidence limits

--------------------------------------------------------------------------

neonata - adult 0.15459 -0.10000 0.40918

neonata - Kind>=5Monate 0.20216 -0.08249 0.48680

neonata - Kind <=2Monate 0.23117 -0.01416 0.47650

neonata - Kind2-<5Monate 0.33392 0.07933 0.58851 ***

adult - neonata -0.15459 -0.40918 0.10000

adult - Kind>=5Monate 0.04757 -0.23708 0.33221

adult - Kind <=2Monate 0.07658 -0.16875 0.32191

adult - Kind2-<5Monate 0.17933 -0.07526 0.43392

Kind>=5Monate - neonata -0.20216 -0.48680 0.08249

Kind>=5Monate - adult -0.04757 -0.33221 0.23708

Kind>=5Monate - Kind <=2Monate 0.02902 -0.24738 0.30541

Kind>=5Monate - Kind2-<5Monate 0.13176 -0.15288 0.41641

Kind <=2Monate - neonata -0.23117 -0.47650 0.01416

Kind <=2Monate - adult -0.07658 -0.32191 0.16875

Kind <=2Monate - Kind>=5Monate -0.02902 -0.30541 0.24738

Kind <=2Monate - Kind2-<5Monate 0.10275 -0.14258 0.34808

Kind2-<5Monate - neonata -0.33392 -0.58851 -0.07933 ***

Kind2-<5Monate - adult -0.17933 -0.43392 0.07526

Kind2-<5Monate - Kind>=5Monate -0.13176 -0.41641 0.15288

Kind2-<5Monate - Kind <=2Monate -0.10275 -0.34808 0.14258

Tukey test of the studentized range (HSD) for **anti-CD3 Ab** CD4^+^**CD45RA^-^**

difference

5 groups between Simultaneous 95%

comparison means confidence limits

----------------------------------------------------------------------------

neonata - Kind>=5Monate 0.27462 -0.09131 0.64054

neonata - adult 0.35405 0.07457 0.63353 ***

neonata - Kind <=2Monate 0.38221 0.06885 0.69557 ***

neonata - Kind2-<5Monate 0.40564 0.10687 0.70442 ***

Kind>=5Monate - neonata -0.27462 -0.64054 0.09131

Kind>=5Monate - adult 0.07943 -0.27091 0.42978

Kind>=5Monate - Kind <=2Monate 0.10759 -0.27033 0.48552

Kind>=5Monate - Kind2-<5Monate 0.13103 -0.23490 0.49695

adult - neonata -0.35405 -0.63353 -0.07457 ***

adult - Kind>=5Monate -0.07943 -0.42978 0.27091

adult - Kind <=2Monate 0.02816 -0.26686 0.32318

adult - Kind2-<5Monate 0.05159 -0.22788 0.33107

Kind <=2Monate - neonata -0.38221 -0.69557 -0.06885 ***

Kind <=2Monate - Kind>=5Monate -0.10759 -0.48552 0.27033

Kind <=2Monate - adult -0.02816 -0.32318 0.26686

Kind <=2Monate - Kind2-<5Monate 0.02343 -0.28992 0.33679

Kind2-<5Monate - neonata -0.40564 -0.70442 -0.10687 ***

Kind2-<5Monate - Kind>=5Monate -0.13103 -0.49695 0.23490

Kind2-<5Monate - adult -0.05159 -0.33107 0.22788

Kind2-<5Monate - Kind <=2Monate -0.02343 -0.33679 0.28992

Tukey test of the studentized range (HSD) for **anti-CD3/CD28 Ab CD4^+^**

difference

5 groups between Simultaneous 95%

comparison means confidence limits

-------------------------------------------------------------------------

neonata - adult 0.12598 -0.12562 0.37758

neonata - Kind <=2Monate 0.25534 -0.00451 0.51519

neonata - Kind>=5Monate 0.32895 0.02425 0.63366 ***

neonata - Kind2-<5Monate 0.39984 0.12938 0.67031 ***

adult - neonata -0.12598 -0.37758 0.12562

adult - Kind <=2Monate 0.12936 -0.12224 0.38097

adult - Kind>=5Monate 0.20297 -0.09472 0.50067

adult - Kind2-<5Monate 0.27387 0.01132 0.53641 ***

Kind <=2Monate - neonata -0.25534 -0.51519 0.00451

Kind <=2Monate - adult -0.12936 -0.38097 0.12224

Kind <=2Monate - Kind>=5Monate 0.07361 -0.23109 0.37831

Kind <=2Monate - Kind2-<5Monate 0.14450 -0.12596 0.41497

Kind>=5Monate - neonata -0.32895 -0.63366 -0.02425 ***

Kind>=5Monate - adult -0.20297 -0.50067 0.09472

Kind>=5Monate - Kind <=2Monate -0.07361 -0.37831 0.23109

Kind>=5Monate - Kind2-<5Monate 0.07089 -0.24291 0.38470

Kind2-<5Monate - neonata -0.39984 -0.67031 -0.12938 ***

Kind2-<5Monate - adult -0.27387 -0.53641 -0.01132 ***

Kind2-<5Monate - Kind <=2Monate -0.14450 -0.41497 0.12596

Kind2-<5Monate - Kind>=5Monate -0.07089 -0.38470 0.24291

Tukey test of the studentized range (HSD) for **anti-CD3 Ab** **CD4^+^**

difference

5 groups between Simultaneous 95%

comparison means confidence limits

---------------------------------------------------------------------

neonata - Kind <=2Monate 0.21978 -0.04354 0.48309

neonata - adult 0.28228 0.05424 0.51032 ***

neonata - Kind>=5Monate 0.34576 0.06135 0.63017 ***

neonata - Kind2-<5Monate 0.43171 0.18345 0.67997 ***

Kind <=2Monate - neonata -0.21978 -0.48309 0.04354

Kind <=2Monate - adult 0.06250 -0.21157 0.33657

Kind <=2Monate - Kind>=5Monate 0.12598 -0.19651 0.44848

Kind <=2Monate - Kind2-<5Monate 0.21193 -0.07917 0.50304

adult - neonata -0.28228 -0.51032 -0.05424 ***

adult - Kind <=2Monate -0.06250 -0.33657 0.21157

adult - Kind>=5Monate 0.06348 -0.23092 0.35788

adult - Kind2-<5Monate 0.14943 -0.11020 0.40907

Kind>=5Monate - neonata -0.34576 -0.63017 -0.06135 ***

Kind>=5Monate - Kind <=2Monate -0.12598 -0.44848 0.19651

Kind>=5Monate - adult -0.06348 -0.35788 0.23092

Kind>=5Monate - Kind2-<5Monate 0.08595 -0.22437 0.39627

Kind2-<5Monate - neonata -0.43171 -0.67997 -0.18345 ***

Kind2-<5Monate - Kind <=2Monate -0.21193 -0.50304 0.07917

Kind2-<5Monate - adult -0.14943 -0.40907 0.11020

Kind2-<5Monate - Kind>=5Monate -0.08595 -0.39627 0.22437

**Anti-CD3 Ab concentration =0.5 μg/ml – ANOVA**

Tukey test of the studentized range (HSD) for **anti-CD3/CD28 Ab** CD4^+^CD45RA^+^**CD31^+^**

difference

5 groups between Simultaneous 95%

comparison means confidence limits

--------------------------------------------------------------------------

neonata - Kind <=2Monate 0.06602 -0.08268 0.21472

neonata - Kind>=5Monate 0.06638 -0.08232 0.21508

neonata - adult 0.07900 -0.05964 0.21764

neonata - Kind2-<5Monate 0.37716 0.22134 0.53298 ***

Kind <=2Monate - neonata -0.06602 -0.21472 0.08268

Kind <=2Monate - Kind>=5Monate 0.00036 -0.16093 0.16165

Kind <=2Monate - adult 0.01298 -0.13909 0.16504

Kind <=2Monate - Kind2-<5Monate 0.31114 0.14326 0.47901 ***

Kind>=5Monate - neonata -0.06638 -0.21508 0.08232

Kind>=5Monate - Kind <=2Monate -0.00036 -0.16165 0.16093

Kind>=5Monate - adult 0.01262 -0.13945 0.16469

Kind>=5Monate - Kind2-<5Monate 0.31078 0.14290 0.47866 ***

adult - neonata -0.07900 -0.21764 0.05964

adult - Kind <=2Monate -0.01298 -0.16504 0.13909

adult - Kind>=5Monate -0.01262 -0.16469 0.13945

adult - Kind2-<5Monate 0.29816 0.13913 0.45719 ***

Kind2-<5Monate - neonata -0.37716 -0.53298 -0.22134 ***

Kind2-<5Monate - Kind <=2Monate -0.31114 -0.47901 -0.14326 ***

Kind2-<5Monate - Kind>=5Monate -0.31078 -0.47866 -0.14290 ***

Kind2-<5Monate - adult -0.29816 -0.45719 -0.13913 ***

Tukey test of the studentized range (HSD) for **anti-CD3 Ab** CD4^+^CD45RA^+^**CD31^+^**

difference

5 groups between Simultaneous 95%

comparison means confidence limits

-------------------------------------------------------------------------

neonate - adult 0.15426 -0.00544 0.31397

neonata - Kind <=2Monate 0.16684 -0.01866 0.35234

neonata - Kind>=5Monate 0.17549 0.00565 0.34532 ***

neonata - Kind2-<5Monate 0.35968 0.17418 0.54519 ***

adult - neonata -0.15426 -0.31397 0.00544

adult - Kind <=2Monate 0.01258 -0.17617 0.20133

adult - Kind>=5Monate 0.02122 -0.15215 0.19460

adult - Kind2-<5Monate 0.20542 0.01667 0.39417 ***

Kind <=2Monate - neonata -0.16684 -0.35234 0.01866

Kind <=2Monate - adult -0.01258 -0.20133 0.17617

Kind <=2Monate - Kind>=5Monate 0.00865 -0.18875 0.20604

Kind <=2Monate - Kind2-<5Monate 0.19284 -0.01818 0.40387

Kind>=5Monate - neonata -0.17549 -0.34532 -0.00565 ***

Kind>=5Monate - adult -0.02122 -0.19460 0.15215

Kind>=5Monate - Kind <=2Monate -0.00865 -0.20604 0.18875

Kind>=5Monate - Kind2-<5Monate 0.18420 -0.01320 0.38160

Kind2-<5Monate - neonata -0.35968 -0.54519 -0.17418 ***

Kind2-<5Monate - adult -0.20542 -0.39417 -0.01667 ***

Kind2-<5Monate - Kind <=2Monate -0.19284 -0.40387 0.01818

Kind2-<5Monate - Kind>=5Monate -0.18420 -0.38160 0.01320

Tukey test of the studentized range (HSD) for **anti-CD3/CD28 Ab** CD4^+^CD45RA^+^**CD31^-^**

difference

5 groups between Simultaneous 95%

comparison means confidence limits

-----------------------------------------------------------------------------

neonata - adult 0.03973 -0.10004 0.17950

neonata - Kind>=5Monate 0.08010 -0.06981 0.23001

neonata - Kind <=2Monate 0.09129 -0.05862 0.24120

neonata - Kind2-<5Monate 0.26503 0.10794 0.42211 ***

adult - neonata -0.03973 -0.17950 0.10004

adult - Kind>=5Monate 0.04037 -0.11293 0.19367

adult - Kind <=2Monate 0.05156 -0.10174 0.20486

adult - Kind2-<5Monate 0.22530 0.06497 0.38562 ***

Kind>=5Monate - neonata -0.08010 -0.23001 0.06981

Kind>=5Monate - adult -0.04037 -0.19367 0.11293

Kind>=5Monate - Kind <=2Monate 0.01119 -0.15141 0.17378

Kind>=5Monate - Kind2-<5Monate 0.18492 0.01569 0.35416 ***

Kind <=2Monate - neonata -0.09129 -0.24120 0.05862

Kind <=2Monate - adult -0.05156 -0.20486 0.10174

Kind <=2Monate - Kind>=5Monate -0.01119 -0.17378 0.15141

Kind <=2Monate - Kind2-<5Monate 0.17374 0.00450 0.34298 ***

Kind2-<5Monate - neonata -0.26503 -0.42211 -0.10794 ***

Kind2-<5Monate - adult -0.22530 -0.38562 -0.06497 ***

Kind2-<5Monate - Kind>=5Monate -0.18492 -0.35416 -0.01569 ***

Kind2-<5Monate - Kind <=2Monate -0.17374 -0.34298 -0.00450 ***

Tukey test of the studentized range (HSD) for **anti-CD3 Ab** CD4^+^CD45RA^+^**CD31^-^**

difference

5 groups between Simultaneous 95%

comparison means confidence limits

------------------------------------------------------------------------

neonata - adult 0.11460 -0.09202 0.32122

neonata - Kind>=5Monate 0.19162 -0.01500 0.39824

neonata - Kind <=2Monate 0.22275 -0.01584 0.46133

neonata - Kind2-<5Monate 0.29752 0.07258 0.52246 ***

adult - neonata -0.11460 -0.32122 0.09202

adult - Kind>=5Monate 0.07702 -0.14078 0.29482

adult - Kind <=2Monate 0.10815 -0.14018 0.35648

adult - Kind2-<5Monate 0.18292 -0.05233 0.41817

Kind>=5Monate - neonata -0.19162 -0.39824 0.01500

Kind>=5Monate - adult -0.07702 -0.29482 0.14078

Kind>=5Monate - Kind <=2Monate 0.03113 -0.21720 0.27946

Kind>=5Monate - Kind2-<5Monate 0.10590 -0.12935 0.34115

Kind <=2Monate - neonata -0.22275 -0.46133 0.01584

Kind <=2Monate - adult -0.10815 -0.35648 0.14018

Kind <=2Monate - Kind>=5Monate -0.03113 -0.27946 0.21720

Kind <=2Monate - Kind2-<5Monate 0.07477 -0.18899 0.33854

Kind2-<5Monate - neonata -0.29752 -0.52246 -0.07258 ***

Kind2-<5Monate - adult -0.18292 -0.41817 0.05233

Kind2-<5Monate - Kind>=5Monate -0.10590 -0.34115 0.12935

Kind2-<5Monate - Kind <=2Monate -0.07477 -0.33854 0.18899

Tukey test of the studentized range (HSD) for **anti-CD3/CD28 Ab** CD4^+^**CD45RA^+^**

difference

5 groups between Simultaneous 95%

comparison means confidence limits

------------------------------------------------------------------------

neonata - adult 0.01018 -0.16050 0.18086

neonata - Kind>=5Monate 0.08321 -0.09346 0.25988

neonata - Kind <=2Monate 0.14944 -0.02723 0.32611

neonata - Kind2-<5Monate 0.29028 0.10593 0.47463 ***

adult - neonata -0.01018 -0.18086 0.16050

adult - Kind>=5Monate 0.07303 -0.10364 0.24970

adult - Kind <=2Monate 0.13926 -0.03741 0.31593

adult - Kind2-<5Monate 0.28010 0.09574 0.46445 ***

Kind>=5Monate - neonata -0.08321 -0.25988 0.09346

Kind>=5Monate - adult -0.07303 -0.24970 0.10364

Kind>=5Monate - Kind <=2Monate 0.06623 -0.11623 0.24869

Kind>=5Monate - Kind2-<5Monate 0.20707 0.01715 0.39698 ***

Kind <=2Monate - neonata -0.14944 -0.32611 0.02723

Kind <=2Monate - adult -0.13926 -0.31593 0.03741

Kind <=2Monate - Kind>=5Monate -0.06623 -0.24869 0.11623

Kind <=2Monate - Kind2-<5Monate 0.14084 -0.04907 0.33075

Kind2-<5Monate - neonata -0.29028 -0.47463 -0.10593 ***

Kind2-<5Monate - adult -0.28010 -0.46445 -0.09574 ***

Kind2-<5Monate - Kind>=5Monate -0.20707 -0.39698 -0.01715 ***

Kind2-<5Monate - Kind <=2Monate -0.14084 -0.33075 0.04907

Tukey test of the studentized range (HSD) for **anti-CD3** CD4^+^**CD45RA^+^**

difference

5 groups between Simultaneous 95%

comparison means confidence limits

--------------------------------------------------------------------

neonata - adult 0.12605 -0.08952 0.34161

neonata - Kind>=5Monate 0.20189 -0.01367 0.41746

neonata - Kind <=2Monate 0.22675 -0.00793 0.46143

neonata - Kind2-<5Monate 0.36507 0.13040 0.59975 ***

adult - neonata -0.12605 -0.34161 0.08952

adult - Kind>=5Monate 0.07584 -0.15138 0.30307

adult - Kind <=2Monate 0.10070 -0.14473 0.34614

adult - Kind2-<5Monate 0.23903 -0.00640 0.48446

Kind>=5Monate - neonata -0.20189 -0.41746 0.01367

Kind>=5Monate - adult -0.07584 -0.30307 0.15138

Kind>=5Monate - Kind <=2Monate 0.02486 -0.22057 0.27029

Kind>=5Monate - Kind2-<5Monate 0.16318 -0.08225 0.40861

Kind <=2Monate - neonata -0.22675 -0.46143 0.00793

Kind <=2Monate - adult -0.10070 -0.34614 0.14473

Kind <=2Monate - Kind>=5Monate -0.02486 -0.27029 0.22057

Kind <=2Monate - Kind2-<5Monate 0.13832 -0.12406 0.40070

Kind2-<5Monate - neonata -0.36507 -0.59975 -0.13040 ***

Kind2-<5Monate - adult -0.23903 -0.48446 0.00640

Kind2-<5Monate - Kind>=5Monate -0.16318 -0.40861 0.08225

Kind2-<5Monate - Kind <=2Monate -0.13832 -0.40070 0.12406

Tukey test of the studentized range (HSD) for **anti-CD3/CD28 Ab** CD4^+^**CD45RA^-^**

difference

5 groups between Simultaneous 95%

comparison means confidence limits

----------------------------------------------------------------------

neonata - Kind>=5Monate 0.05427 -0.10379 0.21232

neonata - adult 0.06100 -0.09706 0.21905

neonata - Kind <=2Monate 0.12116 -0.03689 0.27922

neonata - Kind2-<5Monate 0.22598 0.06147 0.39049 ***

Kind>=5Monate - neonata -0.05427 -0.21232 0.10379

Kind>=5Monate - adult 0.00673 -0.15133 0.16478

Kind>=5Monate - Kind <=2Monate 0.06690 -0.09116 0.22495

Kind>=5Monate - Kind2-<5Monate 0.17172 0.00721 0.33623 ***

adult - neonata -0.06100 -0.21905 0.09706

adult - Kind>=5Monate -0.00673 -0.16478 0.15133

adult - Kind <=2Monate 0.06017 -0.09789 0.21822

adult - Kind2-<5Monate 0.16499 0.00048 0.32950 ***

Kind <=2Monate - neonata -0.12116 -0.27922 0.03689

Kind <=2Monate - Kind>=5Monate -0.06690 -0.22495 0.09116

Kind <=2Monate - adult -0.06017 -0.21822 0.09789

Kind <=2Monate - Kind2-<5Monate 0.10482 -0.05969 0.26933

Kind2-<5Monate - neonata -0.22598 -0.39049 -0.06147 ***

Kind2-<5Monate - Kind>=5Monate -0.17172 -0.33623 -0.00721 ***

Kind2-<5Monate - adult -0.16499 -0.32950 -0.00048 ***

Kind2-<5Monate - Kind <=2Monate -0.10482 -0.26933 0.05969

Tukey test of the studentized range (HSD) for **anti-CD3** CD4^+^**CD45RA^-^**

difference

5 groups between Simultaneous 95%

comparison means confidence limits

----------------------------------------------------------------------------------

neonata - adult 0.16639 -0.07168 0.40446

neonata - Kind>=5Monate 0.24962 0.00374 0.49550 ***

neonata - Kind <=2Monate 0.32091 0.06499 0.57683 ***

neonata - Kind2-<5Monate 0.32904 0.07312 0.58496 ***

adult - neonata -0.16639 -0.40446 0.07168

adult - Kind>=5Monate 0.08323 -0.15484 0.32130

adult - Kind <=2Monate 0.15453 -0.09390 0.40295

adult - Kind2-<5Monate 0.16265 -0.08578 0.41108

Kind>=5Monate - neonata -0.24962 -0.49550 -0.00374 ***

Kind>=5Monate - adult -0.08323 -0.32130 0.15484

Kind>=5Monate - Kind <=2Monate 0.07130 -0.18462 0.32722

Kind>=5Monate - Kind2-<5Monate 0.07942 -0.17650 0.33534

Kind <=2Monate - neonata -0.32091 -0.57683 -0.06499 ***

Kind <=2Monate - adult -0.15453 -0.40295 0.09390

Kind <=2Monate - Kind>=5Monate -0.07130 -0.32722 0.18462

Kind <=2Monate - Kind2-<5Monate 0.00812 -0.25746 0.27370

Kind2-<5Monate - neonata -0.32904 -0.58496 -0.07312 ***

Kind2-<5Monate - adult -0.16265 -0.41108 0.08578

Kind2-<5Monate - Kind>=5Monate -0.07942 -0.33534 0.17650

Kind2-<5Monate - Kind <=2Monate -0.00812 -0.27370 0.25746

Tukey test of the studentized range (HSD) for **anti-CD3/CD28 Ab** **CD4^+^**

difference

5 groups between Simultaneous 95%

comparison means confidence limits

---------------------------------------------------------------------

neonata - Kind>=5Monate 0.05253 -0.11653 0.22159

neonata - adult 0.07646 -0.08116 0.23408

neonata - Kind <=2Monate 0.15259 -0.01647 0.32165

neonata - Kind2-<5Monate 0.25191 0.07475 0.42906 ***

Kind>=5Monate - neonata -0.05253 -0.22159 0.11653

Kind>=5Monate - adult 0.02393 -0.14895 0.19682

Kind>=5Monate - Kind <=2Monate 0.10006 -0.08331 0.28343

Kind>=5Monate - Kind2-<5Monate 0.19938 0.00852 0.39024 ***

adult - neonata -0.07646 -0.23408 0.08116

adult - Kind>=5Monate -0.02393 -0.19682 0.14895

adult - Kind <=2Monate 0.07613 -0.09676 0.24901

adult - Kind2-<5Monate 0.17545 -0.00536 0.35625

Kind <=2Monate - neonata -0.15259 -0.32165 0.01647

Kind <=2Monate - Kind>=5Monate -0.10006 -0.28343 0.08331

Kind <=2Monate - adult -0.07613 -0.24901 0.09676

Kind <=2Monate - Kind2-<5Monate 0.09932 -0.09154 0.29018

Kind2-<5Monate - neonata -0.25191 -0.42906 -0.07475 ***

Kind2-<5Monate - Kind>=5Monate -0.19938 -0.39024 -0.00852 ***

Kind2-<5Monate - adult -0.17545 -0.35625 0.00536

Kind2-<5Monate - Kind <=2Monate -0.09932 -0.29018 0.09154

Tukey test of the studentized range (HSD) for **anti-CD3 Ab** **CD4^+^**

difference

5 groups between Simultaneous 95%

comparison means confidence limits

-------------------------------------------------------------------------

neonata - Kind <=2Monate 0.14414 -0.07538 0.36366

neonata - adult 0.16323 -0.03776 0.36421

neonata - Kind>=5Monate 0.16710 -0.03389 0.36808

neonata - Kind2-<5Monate 0.28212 0.06260 0.50164 ***

Kind <=2Monate - neonata -0.14414 -0.36366 0.07538

Kind <=2Monate - adult 0.01909 -0.21451 0.25269

Kind <=2Monate - Kind>=5Monate 0.02296 -0.21064 0.25655

Kind <=2Monate - Kind2-<5Monate 0.13798 -0.11175 0.38771

adult - neonata -0.16323 -0.36421 0.03776

adult - Kind <=2Monate -0.01909 -0.25269 0.21451

adult - Kind>=5Monate 0.00387 -0.21240 0.22014

adult - Kind2-<5Monate 0.11889 -0.11471 0.35249

Kind>=5Monate - neonata -0.16710 -0.36808 0.03389

Kind>=5Monate - Kind <=2Monate -0.02296 -0.25655 0.21064

Kind>=5Monate - adult -0.00387 -0.22014 0.21240

Kind>=5Monate - Kind2-<5Monate 0.11502 -0.11858 0.34862

Kind2-<5Monate - neonata -0.28212 -0.50164 -0.06260 ***

Kind2-<5Monate - Kind <=2Monate -0.13798 -0.38771 0.11175

Kind2-<5Monate - adult -0.11889 -0.35249 0.11471

Kind2-<5Monate - Kind>=5Monate -0.11502 -0.34862 0.11858

Tukey test of the studentized range (HSD) for **anti-CD3/CD28 Ab** **CD4^-^**

difference

5 groups between Simultaneous 95%

comparison means confidence limits

-----------------------------------------------------------------------

Kind2-<5Monate - adult 0.04131 -0.05389 0.13651

Kind2-<5Monate - neonata 0.04158 -0.05170 0.13485

Kind2-<5Monate - Kind>=5Monate 0.05748 -0.04302 0.15797

Kind2-<5Monate - Kind <=2Monate 0.06484 -0.03565 0.16533

adult - Kind2-<5Monate -0.04131 -0.13651 0.05389

adult - neonata 0.00027 -0.08272 0.08326

adult - Kind>=5Monate 0.01617 -0.07486 0.10720

adult - Kind <=2Monate 0.02353 -0.06750 0.11456

neonata - Kind2-<5Monate -0.04158 -0.13485 0.05170

neonata - adult -0.00027 -0.08326 0.08272

neonata - Kind>=5Monate 0.01590 -0.07312 0.10491

neonata - Kind <=2Monate 0.02326 -0.06575 0.11227

Kind>=5Monate - Kind2-<5Monate -0.05748 -0.15797 0.04302

Kind>=5Monate - adult -0.01617 -0.10720 0.07486

Kind>=5Monate - neonata -0.01590 -0.10491 0.07312

Kind>=5Monate - Kind <=2Monate 0.00736 -0.08919 0.10391

Kind <=2Monate - Kind2-<5Monate -0.06484 -0.16533 0.03565

Kind <=2Monate - adult -0.02353 -0.11456 0.06750

Kind <=2Monate - neonata -0.02326 -0.11227 0.06575

Kind <=2Monate - Kind>=5Monate -0.00736 -0.10391 0.08919
