# Supplementary material for: T Cells of Infants Are Mature, but Hyporeactive Due to Limited Ca2+ Influx
Source: PLoS One. 2016 Nov 28;11(11):e0166633. doi: 10.1371/journal.pone.0166633 (PMC5125607; doi:10.1371/journal.pone.0166633)
Supplement: S10 Table — (DOCX) [file pone.0166633.s019.docx]

## S10 Table

**Cytokine productions of naive T cells after TCR-ligation**.

|  | **Condition** | **CB**  **(n = 9)** | **Infant**  **1-2 mo**  **(n = 13)** | **Infant**  **3-5 mo**  **(n = 18)** | **Infant/**  **Child**  **6-66 mo**  **(n = 17)** | **Adult**  **CD31^+^**  **(n = 9)** | **Adult**  **CD31^-^**  **(n = 4)** |
| --- | --- | --- | --- | --- | --- | --- | --- |
| **IFNγ (pg/ml)** | unstim. | 33 ± 25 | 66 ± 28 | 58 ± 8,9 | 55 ± 29 | 34 ± 23 | 36 ± 1.8 |
|  | anti-CD3 | 1082 ± 595 | 1167 ± 528 | 749 ± 106 | 813 ± 255 | 1429 ± 536 | 1976 ± 244 |
|  | anti-CD3/CD28 | 1387 ± 800 | 1300 ± 620 | 856 ± 88 | 815 ± 82 | 1499 ± 551 | 2116 ± 166 |
| **IL-2 (pg/ml)** | unstim. | 8.5 ± 6.3 | 3.8 ± 2.2 | 3.9 ± 1.5 | 3.5 ± 1.3 | 14.1 ± 9.6 | 5.8 ± 3.4 |
|  | anti-CD3 | 199 ± 313 | 548 ± 665 | 30 ± 8,6 | 32 ± 3,1 | 610 ± 494 | 1087 ± 301 |
|  | anti-CD3/CD28 | 243 ± 392 | 913 ± 741 | 200 ± 208 | 218 ± 283 | 1366 ± 1163 | 1674 ± 492 |
| **TNFα (pg/ml)** | unstim. | 8.5 ± 7.5 | 20 ± 32 | 9.3 ± 9.5 | 6.4 ± 7.4 | 9.4 ± 8.9 | 5.8 ± 3.6 |
|  | anti-CD3 | 505 ± 594 | 1496 ± 1657 | 90 ± 172 | 395 ± 1027 | 2038 ± 1893 | 881 ± 272 |
|  | anti-CD3/CD28 | 701 ± 759 | 1173 ± 1019 | 414 ± 596 | 1169 ± 1943 | 3113 ± 1680 | 2103 ± 810 |

Cytokine productions of naive T cells after TCR-ligation in five age groups, supernatant after 48 h stimulation with 0.5 µg/ml anti-CD3- and anti-CD28 ab (CD28) respectively only anti-CD3 ab (CD3) after cross-linking with 10 µg/ml polyclonal-Goat-Anti-Mouse IgG (GAMIg) determinate by using Bio-Plex cytokine assay with the human single Plex Panel (IFNγ, IL-2, TNFα) (Bio-Rad). CD31^+^ = CD4^+^CD45RA^+^CD31^+^; CD31^-^ = CD4^+^CD45RA^+^CD31^-^; number (n) of sample and the main parameters in form of mean ± standard deviation. mo = months.
