# Supplementary material for: T Cells of Infants Are Mature, but Hyporeactive Due to Limited Ca2+ Influx
Source: PLoS One. 2016 Nov 28;11(11):e0166633. doi: 10.1371/journal.pone.0166633 (PMC5125607; doi:10.1371/journal.pone.0166633)
Supplement: S11 Table — (DOCX) [file pone.0166633.s020.docx]

## S11 Table

**Summary of ANOVA assessment for cytokine production for differences of stimulation (unstimulated, anti-CD3/CD28 and anti-CD3) for 5 groups of individual.**

| **Cytokine**  **(pg/ml)** | **CB** | **Infant**  **1-2 mo** | **Infant**  **3-5 mo** | **Infant**  **/Child**  **6-66 mo** | **Adult**  **CD31^+^** |
| --- | --- | --- | --- | --- | --- |
| **IFNγ** | 0.1778 | 0.5444 | 0.0019 | 0.9272 | 0.3000 |
| **IL-2** | 0.1686 | 0.2146 | 0.0138 | 0.1371 | 0.0113* |
| **TNFα** | 0.1763 | 0.3050 | 0.0350 | 0.0904 | 0.0285 |

* At unadjusted pair comparison P = 0.0729 (n = 8) was chosen with extremely oblique distribution logarithm P = 0.0113; mo = months.
