# Supplementary material for: T Cells of Infants Are Mature, but Hyporeactive Due to Limited Ca2+ Influx
Source: PLoS One. 2016 Nov 28;11(11):e0166633. doi: 10.1371/journal.pone.0166633 (PMC5125607; doi:10.1371/journal.pone.0166633)
Supplement: S12 Table — (DOCX) [file pone.0166633.s021.docx]

## S12 Table

**Summary of significant differences of the two-tailed ANOVA of differences for cytokine production of stimulation (unstimulated, anti-CD3/CD28 and anti-CD3) for 5 groups with infants compared to CB and adults of the CD4^+^ CD45RA^+^ CD31^+^ T cells.**

| **Cyto-kine** | **Difference of condition** | **CB** | **Infant**  **1-2 mo** | **Infant**  **3-5 mo** | **Infant/ Child**  **6-66 mo** | **Adult**  **CD31^+^** | **Adult**  **CD31^-^** |
| --- | --- | --- | --- | --- | --- | --- | --- |
| **IFNγ** | unstim. **–** anti-CD3/CD28 | 0,002 | 0,001 | <0,0001 | <0,0001 | <0,0001 | <0,0001 |
|  | unstim. **–**  anti-CD3 | 0,003 | 0,003 | <0,0001 | <0,0001 | <0,0001 | 0,005 |
|  | anti-CD3/CD28 – anti-CD3 | 0,178 | 0,544 | 0,008 | 0,675 | 0,974 | 0,734 |
| **IL-2** | unstim. – anti-CD3/CD28 | 0,107 | 0,015 | 0,010 | 0,037 | 0,001 | 0,006 |
|  | unstim. – anti-CD3 | 0,101 | 0,026 | <0,0001 | <0,0001 | 0,032 | 0,006 |
|  | anti-CD3/CD28 – anti-CD3 | 0,169 | 0,215 | 0,017 | 0,112 | 0,041 | 0,133 |
| **TNFα** | unstim. – anti-CD3/CD28 | 0,051 | 0,069 | 0,039 | 0,056 | 0,001 | 0,014 |
|  | unstim. – anti-CD3 | 0,067 | 0,064 | 0,091 | 0,178 | 0,023 | 0,007 |
|  | anti-CD3/CD28 – anti-CD3 | 0,176 | 0,305 | 0,041 | 0,086 | 0,011 | 0,105 |
