# Supplementary material for: T Cells of Infants Are Mature, but Hyporeactive Due to Limited Ca2+ Influx
Source: PLoS One. 2016 Nov 28;11(11):e0166633. doi: 10.1371/journal.pone.0166633 (PMC5125607; doi:10.1371/journal.pone.0166633)
Supplement: S13 Table — (DOCX) [file pone.0166633.s022.docx]

## S13 Table

**Concrete single analysis of ANOVA assessment for cytokine for 5 groups of individual (CB, infants 1-2 mo, infants 3-5 mo, infants 6-66 mo, adult) differences between stimulation of anti-CD3/CD28 Ab group with Anti-CD3 Ab group.**

5 groups = CB

standart

Variable N mean deviation Minimum Maximum t-value Pr > |t|

------------------------------------------------------------------------------------------

DIFN_CD28_CD3_31p 7 416.864 722.620 -227.268 1804.020 1.53 0.1778

DIL2_CD28_CD3_31p 9 44.016 87.234 -30.890 260.280 1.51 0.1686

DTNF_CD28_CD3_31p 7 196.356 339.002 -74.000 875.500 1.53 0.1763

5 groups = Infant1-2 months

standart

Variable N mean deviation Minimum Maximum t-value Pr > |t|

------------------------------------------------------------------------------------------

DIFN_CD28_CD3_31p 7 -160.544 661.246 -1160.445 875.533 -0.64 0.5444

DIL2_CD28_CD3_31p 7 327.429 624.321 -164.380 1692.350 1.39 0.2146

DTNF_CD28_CD3_31p 6 -907.470 1945.569 -3408.500 713.780 -1.14 0.3050

5 groups = Infant3-5 months

standart

Variable N mean deviation Minimum Maximum t-value Pr > |t||

------------------------------------------------------------------------------------------

DIFN_CD28_CD3_31p 12 142.227 121.174 0.000 379.840 4.07 0.0019

DIL2_CD28_CD3_31p 12 181.428 214.950 16.030 613.860 2.92 0.0138

DTNF_CD28_CD3_31p 12 325.936 469.682 32.780 1387.500 2.40 0.0350

5 groups = Infant6-66 months

standart

Variable N mean deviation Minimum Maximum t-value Pr > |t|

------------------------------------------------------------------------------------------

DIFN_CD28_CD3_31p 11 7.609 269.292 -764.196 210.690 0.09 0.9272

DIL2_CD28_CD3_31p 9 157.779 286.500 15.280 906.000 1.65 0.1371

DTNF_CD28_CD3_31p 11 846.136 1497.270 23.140 5164.890 1.87 0.0904

5 groups = Adult

standart<

Variable N mean deviation Minimum Maximum t-value Pr > |t|

------------------------------------------------------------------------------------------

DIFN_CD28_CD3_31p 9 69.957 189.377 -121.115 504.959 1.11 0.3000

DIL2_CD28_CD3_31p 8 755.636 1013.415 -186.800 3136.965 2.11 0.0113*

DTNF_CD28_CD3_31p 7 1335.216 1232.079 -727.935 3117.000 2.87 0.0285

* Logarithm was elected with oblique distribution

IFN

Stimulation without CD3 <.0001 Bonferroni <.0001

Stimulation without CD3CD28 <.0001 Bonferroni <.0001

Stimulation CD3 CD3CD28 0.0612 Bonferroni 0.1837

Log(IL-2)*

Stimulation without CD3 <.0001 Bonferroni <.0001

Stimulation without CD3CD28 <.0001 Bonferroni <.0001

Stimulation CD3 CD3CD28 <.0001 Bonferroni <.0001

IL-2

Stimulation without CD3 0.0004 Bonferroni 0.0011

Stimulation without CD3CD28 <.0001 Bonferroni <.0001

Stimulation CD3 CD3CD28 <.0001 Bonferroni <.0001

TNF

Stimulation without CD3 <.0001 Bonferroni <.0001

Stimulation without CD3CD28 <.0001 Bonferroni <.0001

Stimulation CD3 CD3CD28 0.0316 Bonferroni 0.0949
