# Supplementary material for: T Cells of Infants Are Mature, but Hyporeactive Due to Limited Ca2+ Influx
Source: PLoS One. 2016 Nov 28;11(11):e0166633. doi: 10.1371/journal.pone.0166633 (PMC5125607; doi:10.1371/journal.pone.0166633)
Supplement: S14 Table — (DOCX) [file pone.0166633.s023.docx]

## S14 Table

**Summary of ANOVA assessment for cytokine production for 5 groups of individual (CB, infants 1-2 mo, infants 3-5 mo, infants 6-66 mo, adult)**

|  | **IFNγ**  **(pg/ml)** | **IL-2**  **(pg/ml)** | **TNFα**  **(pg/ml)** |
| --- | --- | --- | --- |
| unstimulated | 0.0136 | 0.0002 | 0.3655 |
| anti-CD3 Ab | 0.0007 | 0.0004 | 0.0013 |
| anti-CD3/anti-CD28 Ab | 0.0016 | <0.0001 | 0.0020 |

mo = months
