# Supplementary material for: T Cells of Infants Are Mature, but Hyporeactive Due to Limited Ca2+ Influx
Source: PLoS One. 2016 Nov 28;11(11):e0166633. doi: 10.1371/journal.pone.0166633 (PMC5125607; doi:10.1371/journal.pone.0166633)
Supplement: S15 Table — (DOCX) [file pone.0166633.s024.docx]

## S15 Table

**Summary of significant differences of the Tukey test in the cytokine production (*) of infants (ages in months: 1-2, 3-5 or 6-66) compared to CB and adults of the CD4^+^CD45RA^+^CD31^+^ T cells on stimulation (unstimulated, anti-CD3/CD28 Ab, anti-CD3 Ab).**

|  | Cytokine production | anti-CD3 Ab concentration 0.5 μg/ml | | | | | | | | | |
| --- | --- | --- | --- | --- | --- | --- | --- | --- | --- | --- | --- |
|  | (pg/ml) |  | CB | | | Adult | | | Infant | | |
|  |  | Adult | Infant1-2 | Infant 3-5 | Infant 6-66 | Infant 1-2 | Infant 3-5 | Infant 6-66 | 1-2  Infant 3-5 | 3-5  Infant  6-66 | 1-2  Infant  6-66 |
| unstim. | IFNγ | **-** | ***** | **-** | **-** | **-** | **-** | **-** | **-** | **-** | **-** |
|  | IL-2 | **-** | **-** | **-** | **-** | ***** | ***** | ***** | **-** | **-** | **-** |
|  | TNFα | **-** | **-** | **-** | **-** | **-** | **-** | **-** | **-** | **-** | **-** |
| anti-CD3/CD28 | IFNγ | **-** | **-** | **-** | **-** | **-** | ***** | ***** | **-** | **-** | **-** |
|  | IL-2 | **-** | **-** | **-** | **-** | ***** | ***** | ***** | **-** | **-** | **-** |
|  | TNFα | ***** | **-** | **-** | **-** | ***** | ***** | ***** | **-** | **-** | **-** |
| anti-CD3 | IFNγ | **-** | **-** | **-** | **-** | **-** | ***** | ***** | **-** | **-** | **-** |
|  | IL-2 | **-** | **-** | **-** | **-** | **-** | ***** | ***** | ***** | **-** | ***** |
|  | TNFα | **-** | **-** | **-** | **-** | **-** | ***** | ***** | ***** | **-** | **-** |

Stochastic comparisons of the data were performed with ANOVA, which was performed as two-tailed tests followed by the Tukey-Kramer post-hoc test. The star (*) indicates pairwise significance (*p*≤0.05). infant age in months.
