# Supplementary material for: T Cells of Infants Are Mature, but Hyporeactive Due to Limited Ca2+ Influx
Source: PLoS One. 2016 Nov 28;11(11):e0166633. doi: 10.1371/journal.pone.0166633 (PMC5125607; doi:10.1371/journal.pone.0166633)
Supplement: S16 Table — (DOCX) [file pone.0166633.s025.docx]

## S16 Table

**Concrete single analysis of ANOVA assessment for cytokine between 5 groups of individual (CB, infants 1-2 mo, infants 3-5 mo, infants 6-66 mo, adult) under three different stimulation (unstimulated, anti-CD3/CD28 Ab, Anti-CD3 Ab group).** Comparisons are significant at the 0,05 level are indicated by ***.

Dependent variable: **IFNγ of unstimulated**

Summe der Mittleres

Quelle DF Quadrate Quadrat F-Statistik Pr > F

------------------------------------------------------------------------------------------

Modell 4 7685.37484 1921.34371 3.56 **0.0136**

Fehler 43 23223.02315 540.07031

Korrigierte Summe 47 30908.39799

Tukey test of the studentized range (HSD) for **IFNγ of unstimulated** of CD4^+^CD45RA^+^CD31^+^

difference

5 groups between Simultaneous 95%

comparison means confidence limits

---------------------------------------------------------------

Infant1_2 - Infant3_5 8.490 -21.708 38.688

Infant1_2 - Infant6_66 11.083 -19.659 41.825

Infant1_2 - ZAdult 32.389 -0.691 65.469

Infant1_2 - neonatal 32.826 0.678 64.974 ***

Infant3_5 - Infant1_2 -8.490 -38.688 21.708

Infant3_5 - Infant6_66 2.593 -25.024 30.209

Infant3_5 - ZAdult 23.899 -6.299 54.097

Infant3_5 - neonatal 24.336 -4.838 53.510

Infant6_66 - Infant1_2 -11.083 -41.825 19.659

Infant6_66 - Infant3_5 -2.593 -30.209 25.024

Infant6_66 - ZAdult 21.306 -9.436 52.048

Infant6_66 - neonatal 21.743 -7.993 51.480

ZAdult - Infant1_2 -32.389 -65.469 0.691

ZAdult - Infant3_5 -23.899 -54.097 6.299

ZAdult - Infant6_66 -21.306 -52.048 9.436

ZAdult - neonatal 0.437 -31.711 32.585

neonatal - Infant1_2 -32.826 -64.974 -0.678 ***

neonatal - Infant3_5 -24.336 -53.510 4.838

neonatal - Infant6_66 -21.743 -51.480 7.993

neonatal - ZAdult -0.437 -32.585 31.711

Dependent variable: **IFNγ of anti-CD3/CD28 Ab**

Summe der Mittleres

Quelle DF Quadrate Quadrat F-Statistik Pr > F

------------------------------------------------------------------------------------------

Modell 4 4330943.22 1082735.81 5.11 **0.0016**

Fehler 48 10172870.81 211934.81

Korrigierte Summe 52 14503814.04

Tukey test of the studentized range (HSD) for **IFNγ of anti-CD3/CD28 Ab** of CD4^+^CD45RA^+^CD31^+^

difference

5 groups between Simultaneous 95%

comparison means confidence limits

--------------------------------------------------------------

ZAdult - neonatal 111.9 -522.1 745.9

ZAdult - Infant1_2 198.9 -416.1 814.0

ZAdult - Infant3_5 643.0 85.5 1200.4 ***

ZAdult - Infant6_66 683.4 117.6 1249.2 ***

neonatal - ZAdult -111.9 -745.9 522.1

neonatal - Infant1_2 87.0 -547.0 721.0

neonatal - Infant3_5 531.0 -47.2 1109.3

neonatal - Infant6_66 571.5 -14.8 1157.8

Infant1_2 - ZAdult -198.9 -814.0 416.1

Infant1_2 - neonatal -87.0 -721.0 547.0

Infant1_2 - Infant3_5 444.1 -113.4 1001.5

Infant1_2 - Infant6_66 484.5 -81.3 1050.3

Infant3_5 - ZAdult -643.0 -1200.4 -85.5 ***

Infant3_5 - neonatal -531.0 -1109.3 47.2

Infant3_5 - Infant1_2 -444.1 -1001.5 113.4

Infant3_5 - Infant6_66 40.4 -462.1 543.0

Infant6_66 - ZAdult -683.4 -1249.2 -117.6 ***

Infant6_66 - neonatal -571.5 -1157.8 14.8

Infant6_66 - Infant1_2 -484.5 -1050.3 81.3

Infant6_66 - Infant3_5 -40.4 -543.0 462.1

Dependent variable: **IFNγ of anti-CD3 Ab**

Summe der Mittleres

Quelle DF Quadrate Quadrat F-Statistik Pr > F

------------------------------------------------------------------------------------------

Modell 4 3534051.40 883512.85 5.65 **0.0007**

Fehler 53 8285316.11 156326.72

Korrigierte Summe 57 11819367.51

Tukey test of the studentized range (HSD) for **IFNγ of anti-CD3 Ab** of CD4^+^CD45RA^+^CD31^+^

difference

5 groups between Simultaneous 95%

comparison means confidence limits

-----------------------------------------------------------

ZAdult - Infant1_2 261.6 -240.2 763.5

ZAdult - neonatal 347.0 -215.6 909.7

ZAdult - Infant6_66 615.2 144.4 1086.0 ***

ZAdult - Infant3_5 679.9 214.7 1145.1 ***

Infant1_2 - ZAdult -261.6 -763.5 240.2

Infant1_2 - neonatal 85.4 -454.4 625.3

Infant1_2 - Infant6_66 353.6 -89.6 796.8

Infant1_2 - Infant3_5 418.3 -19.0 855.6

neonatal - ZAdult -347.0 -909.7 215.6

neonatal - Infant1_2 -85.4 -625.3 454.4

neonatal - Infant6_66 268.2 -242.9 779.2

neonatal - Infant3_5 332.9 -173.1 838.8

Infant6_66 - ZAdult -615.2 -1086.0 -144.4 ***

Infant6_66 - Infant1_2 -353.6 -796.8 89.6

Infant6_66 - neonatal -268.2 -779.2 242.9

Infant6_66 - Infant3_5 64.7 -336.6 466.0

Infant3_5 - ZAdult -679.9 -1145.1 -214.7 ***

Infant3_5 - Infant1_2 -418.3 -855.6 19.0

Infant3_5 - neonatal -332.9 -838.8 173.1

Infant3_5 - Infant6_66 -64.7 -466.0 336.6

Dependent variable: **IL-2 of unstimulated**

Summe der Mittleres

Quelle DF Quadrate Quadrat F-Statistik Pr > F

------------------------------------------------------------------------------------------

Modell 4 688.847005 172.211751 7.30 **0.0002**

Fehler 41 967.677764 23.601897

Korrigierte Summe 45 1656.524769

Tukey test of the studentized range (HSD) for **IL-2 of unstimulated** of CD4^+^CD45RA^+^CD31^+^

difference

5 groups between Simultaneous 95%

comparison means confidence limits

----------------------------------------------------------------

ZAdult - neonatal 5.576 -1.409 12.560

ZAdult - Infant3_5 10.258 3.667 16.850 ***

ZAdult - Infant1_2 10.567 3.394 17.740 ***

ZAdult - Infant6_66 10.724 3.894 17.555 ***

neonatal - ZAdult -5.576 -12.560 1.409

neonatal - Infant3_5 4.683 -1.429 10.794

neonatal - Infant1_2 4.991 -1.743 11.726

neonatal - Infant6_66 5.149 -1.219 11.517

Infant3_5 - ZAdult -10.258 -16.850 -3.667 ***

Infant3_5 - neonatal -4.683 -10.794 1.429

Infant3_5 - Infant1_2 0.309 -6.017 6.635

Infant3_5 - Infant6_66 0.466 -5.468 6.401

Infant1_2 - ZAdult -10.567 -17.740 -3.394 ***

Infant1_2 - neonatal -4.991 -11.726 1.743

Infant1_2 - Infant3_5 -0.309 -6.635 6.017

Infant1_2 - Infant6_66 0.158 -6.417 6.732

Infant6_66 - ZAdult -10.724 -17.555 -3.894 ***

Infant6_66 - neonatal -5.149 -11.517 1.219

Infant6_66 - Infant3_5 -0.466 -6.401 5.468

Infant6_66 - Infant1_2 -0.158 -6.732 6.417

Dependent variable: **IL-2 of anti-CD3/CD28 Ab**

Summe der Mittleres

Quelle DF Quadrate Quadrat F-Statistik Pr > F

-----------------------------------------------------------------------------------------

Modell 4 10410823.37 2602705.84 7.52 **<.0001**

Fehler 48 16617713.29 346202.36

Korrigierte Summe 52 27028536.66

Tukey test of the studentized range (HSD) for **IL-2 of anti-CD3/CD28 Ab** of CD4^+^CD45RA^+^CD31^+^

difference

5 groups between Simultaneous 95%

comparison means confidence limits

--------------------------------------------------------------------

ZAdult - Infant1_2 453.3 -357.0 1263.6

ZAdult - neonatal 1123.1 312.8 1933.4 ***

ZAdult - Infant6_66 1147.6 398.3 1897.0 ***

ZAdult - Infant3_5 1165.6 426.5 1904.7 ***

Infant1_2 - ZAdult -453.3 -1263.6 357.0

Infant1_2 - neonatal 669.8 -116.3 1455.9

Infant1_2 - Infant6_66 694.3 -28.8 1417.4

Infant1_2 - Infant3_5 712.3 -0.2 1424.8

neonatal - ZAdult -1123.1 -1933.4 -312.8 ***

neonatal - Infant1_2 -669.8 -1455.9 116.3

neonatal - Infant6_66 24.5 -698.6 747.6

neonatal - Infant3_5 42.5 -670.0 754.9

Infant6_66 - ZAdult -1147.6 -1897.0 -398.3 ***

Infant6_66 - Infant1_2 -694.3 -1417.4 28.8

Infant6_66 - neonatal -24.5 -747.6 698.6

Infant6_66 - Infant3_5 18.0 -624.3 660.3

Infant3_5 - ZAdult -1165.6 -1904.7 -426.5 ***

Infant3_5 - Infant1_2 -712.3 -1424.8 0.2

Infant3_5 - neonatal -42.5 -754.9 670.0

Infant3_5 - Infant6_66 -18.0 -660.3 624.3

Dependent variable: **IL-2 of anti-CD3 Ab**

Summe der Mittleres

Quelle DF Quadrate Quadrat F-Statistik Pr > F

------------------------------------------------------------------------------------------

Modell 4 3377340.73 844335.18 6.22 **0.0004**

Fehler 51 6917999.53 135647.05

Korrigierte Summe 55 10295340.26

Tukey test of the studentized range (HSD) for **IL-2 of anti-CD3 Ab** of CD4^+^CD45RA^+^CD31^+^

difference

5 groups between Simultaneous 95%

comparison means confidence limits

-----------------------------------------------------------------

ZAdult - Infant1_2 62.3 -421.7 546.2

ZAdult - neonatal 411.5 -94.5 917.6

ZAdult - Infant6_66 578.5 110.5 1046.5 ***

ZAdult - Infant3_5 579.8 123.8 1035.7 ***

Infant1_2 - ZAdult -62.3 -546.2 421.7

Infant1_2 - neonatal 349.2 -118.9 817.3

Infant1_2 - Infant6_66 516.2 89.6 942.9 ***

Infant1_2 - Infant3_5 517.5 104.1 930.9 ***

neonatal - ZAdult -411.5 -917.6 94.5

neonatal - Infant1_2 -349.2 -817.3 118.9

neonatal - Infant6_66 167.0 -284.6 618.6

neonatal - Infant3_5 168.3 -270.9 607.4

Infant6_66 - ZAdult -578.5 -1046.5 -110.5 ***

Infant6_66 - Infant1_2 -516.2 -942.9 -89.6 ***

Infant6_66 - neonatal -167.0 -618.6 284.6

Infant6_66 - Infant3_5 1.3 -393.4 395.9

Infant3_5 - ZAdult -579.8 -1035.7 -123.8 ***

Infant3_5 - Infant1_2 -517.5 -930.9 -104.1 ***

Infant3_5 - neonatal -168.3 -607.4 270.9

Infant3_5 - Infant6_66 -1.3 -395.9 393.4

Dependent variable: **TNFα of unstimulated**

Summe der Mittleres

Quelle DF Quadrate Quadrat F-Statistik Pr > F

-------------------------------------------------------------------------------------------

Modell 4 889.906391 222.476598 1.11 0.3655

Fehler 43 8639.773307 200.924961

Korrigierte Summe 47 9529.679698

Dependent variable: **TNFα of anti-CD3/CD28 Ab**

Summe der Mittleres

Quelle DF Quadrate Quadrat F-Statistik Pr > F

------------------------------------------------------------------------------------------

Modell 4 39053196.5 9763299.1 5.36 **0.0013**

Fehler 44 80083259.4 1820074.1

Korrigierte Summe 48 119136455.9

Tukey test of the studentized range (HSD) for **TNFα of anti-CD3/CD28 Ab** of CD4^+^CD45RA^+^CD31^+^

difference

5 groups between Simultaneous 95%

comparison means confidence limits

-------------------------------------------------------------------

ZAdult - Infant1_2 1940.2 21.7 3858.7 ***

ZAdult - Infant6_66 1944.5 220.3 3668.7 ***

ZAdult - neonatal 2412.1 426.3 4397.9 ***

ZAdult - Infant3_5 2699.1 974.9 4423.3 ***

Infant1_2 - ZAdult -1940.2 -3858.7 -21.7 ***

Infant1_2 - Infant6_66 4.3 -1719.9 1728.5

Infant1_2 - neonatal 471.9 -1513.9 2457.7

Infant1_2 - Infant3_5 758.9 -965.3 2483.1

Infant6_66 - ZAdult -1944.5 -3668.7 -220.3 ***

Infant6_66 - Infant1_2 -4.3 -1728.5 1719.9

Infant6_66 - neonatal 467.6 -1331.2 2266.4

Infant6_66 - Infant3_5 754.6 -750.4 2259.6

neonatal - ZAdult -2412.1 -4397.9 -426.3 ***

neonatal - Infant1_2 -471.9 -2457.7 1513.9

neonatal - Infant6_66 -467.6 -2266.4 1331.2

neonatal - Infant3_5 287.0 -1511.8 2085.8

Infant3_5 - ZAdult -2699.1 -4423.3 -974.9 ***

Infant3_5 - Infant1_2 -758.9 -2483.1 965.3

Infant3_5 - Infant6_66 -754.6 -2259.6 750.4

Infant3_5 - neonatal -287.0 -2085.8 1511.8

Dependent variable: **TNFα of anti-CD3 Ab**

Summe der Mittleres

Quelle DF Quadrate Quadrat F-Statistik Pr > F

--------------------------------------------------------------------------------------------

Modell 4 25338267.23 6334566.81 4.99 **0.0020**

Fehler 47 59695665.02 1270120.53

Korrigierte Summe 51 85033932.25

Tukey test of the studentized range (HSD) for **TNFα of anti-CD3 Ab** of CD4^+^CD45RA^+^CD31^+^

difference

5 groups between Simultaneous 95%

comparison means confidence limits

-----------------------------------------------------------------

ZAdult - Infant1_2 542.0 -1069.0 2153.0

ZAdult - neonatal 1533.0 -175.7 3241.7

ZAdult - Infant6_66 1643.3 163.5 3123.1 ***

ZAdult - Infant3_5 1948.5 485.2 3411.8 ***

Infant1_2 - ZAdult -542.0 -2153.0 1069.0

Infant1_2 - neonatal 991.0 -620.0 2602.0

Infant1_2 - Infant6_66 1101.3 -264.5 2467.1

Infant1_2 - Infant3_5 1406.5 58.6 2754.3 ***

neonatal - ZAdult -1533.0 -3241.7 175.7

neonatal - Infant1_2 -991.0 -2602.0 620.0

neonatal - Infant6_66 110.3 -1369.5 1590.1

neonatal - Infant3_5 415.5 -1047.8 1878.8

Infant6_66 - ZAdult -1643.3 -3123.1 -163.5 ***

Infant6_66 - Infant1_2 -1101.3 -2467.1 264.5

Infant6_66 - neonatal -110.3 -1590.1 1369.5

Infant6_66 - Infant3_5 305.2 -882.7 1493.1

Infant3_5 - ZAdult -1948.5 -3411.8 -485.2 ***

Infant3_5 - Infant1_2 -1406.5 -2754.3 -58.6 ***

Infant3_5 - neonatal -415.5 -1878.8 1047.8

Infant3_5 - Infant6_66 -305.2 -1493.1 882.7
