# Supplementary material for: T Cells of Infants Are Mature, but Hyporeactive Due to Limited Ca2+ Influx
Source: PLoS One. 2016 Nov 28;11(11):e0166633. doi: 10.1371/journal.pone.0166633 (PMC5125607; doi:10.1371/journal.pone.0166633)
Supplement: S17 Table — (DOCX) [file pone.0166633.s026.docx]

## S17 Table

**Summary of significant differences of the two-tailed ANOVA of differences for cytokine production of stimulation for adults of the CD4^+^CD45RA^+^CD31^+^ T cells (CD31^+^) subgroups compared to adult the CD4^+^CD45RA^+^CD31^-^ T cells (CD31^-^).**

|  | **Cytokine production (pg/ml)** | **Adult CD31^+^**  **compared to**  **Adult CD31^-^** |
| --- | --- | --- |
| unstim. | IFNγ | 0,847 |
|  | IL-2 | 0,134 |
|  | TNFα | 0,516 |
| anti-CD3/  anti-CD28 | IFNγ | 0,032 |
|  | IL-2 | 0,629 |
|  | TNFα | 0,210 |
| anti-CD3 | IFNγ | 0,316 |
|  | IL-2 | 0,111 |
|  | TNFα | 0,295 |
